# Supplementary material for: A fragment-based approach identifies an allosteric pocket that impacts malate dehydrogenase activity
Source: Commun Biol. 2021 Aug 10;4:949. doi: 10.1038/s42003-021-02442-1 (PMC8355244; doi:10.1038/s42003-021-02442-1)
Supplement: Supplementary file 2 — Supplementary Information [file 42003_2021_2442_MOESM2_ESM.pdf]

Supplementary Information

for

A Fragment-Based Approach Identifies an Allosteric Pocket that Impacts Malate Dehydrogenase Activity

Table of Contents

Supplementary Table 1 .... S3
Supplementary Table 2 .... S4
Supplementary Fig. 1 .... S7
Supplementary Fig. 2 .... S8
Supplementary Table 3 .... S9
Supplementary Table 4 .... S9
Supplementary Table 5 .... S9
Supplementary Table 6 .... S9
Supplementary Fig. 3 .... S9
Supplementary Table 7 .... S10
Supplementary Fig. 4 .... S10
Supplementary Table 8 .... S10
Supplementary Methods 1: Docking of 4DT in *Hs*MDH1 and *Hs*MDH2 .... S11
Supplementary Fig. 5 .... S12
Supplementary Methods 2: Homology model: validation and evaluation S13
Supplementary Fig. 6 .... S14
Supplementary References S17

Supplementary Table 1. Top ten MDH antagonists with anticancer activity sorted according to their increasing potency.

Name	Structure	IC_{50} (μM)	Target name	Reference
Inhibitor **4k**	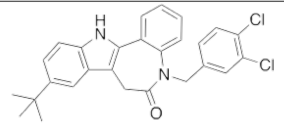	0.770 ± 0.02	*Hs*MDH2	10.1016/j.ejmech.2009.10.018
Inhibitor **8e**	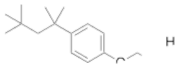	0.92± 0.28	*Hs*MDH1	10.1021/acs.jmedchem.7b01231
Inhibitor **16c**	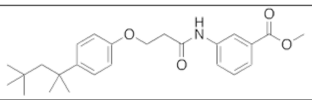	1.06± 0.03	*Hs*MDH2	10.1021/acs.jmedchem.7b01231
Inhibitor **16**c	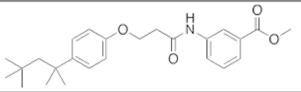	1.07± 0.07	*Hs*MDH1	10.1021/acs.jmedchem.7b01231
Inhibitor **4h**	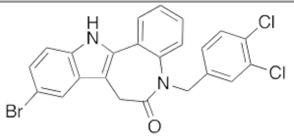	1.10± 0.10	*Hs*MDH2	10.1016/j.ejmech.2009.10.018
Inhibitor **1**	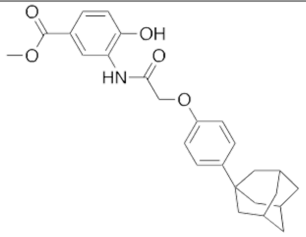	1.10± 0.10	*Hs*MDH1	10.1021/acs.jmedchem.7b01231
Inhibitor **14c**	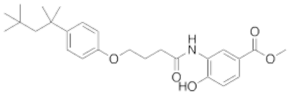	1.11 ± 0.01	*Hs*MDH2	10.1021/acs.jmedchem.7b01231
Inhibitor **14b**	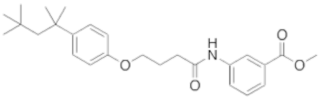	1.11 ± 0.01	*Hs*MDH2	10.1021/acs.jmedchem.7b01231
Inhibitor **6**	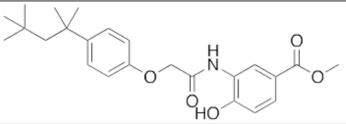	1.14± 0.09	*Hs*MDH1	10.1021/acs.jmedchem.7b01231
Inhibitor **14b**	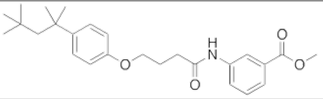	1.18± 0.08	*Hs*MDH1	10.1021/acs.jmedchem.7b01231

Supplementary Table 2 Structure of bioactive compounds against *Pf*MDH sorted according to increasing potency.

Name	Structure	IC_{50} (μM)	DOI
Oxamate	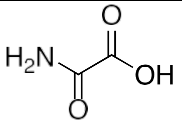	> 2000	10.1021/jm070336k
Inhibitor **2**	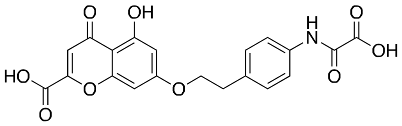	> 241	10.1021/jm070336k
Inhibitor **4**	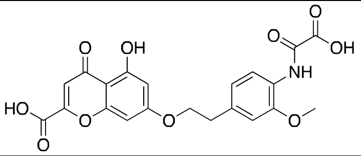	> 225	10.1021/jm070336k
Inhibitor **6**	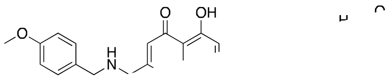	86.90± 0.46	10.1021/jm070336k
Inhibitor **1**	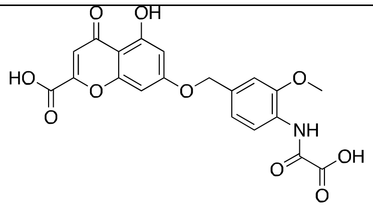	48.30± 0.30	10.1021/jm070336k
Inhibitor **5**	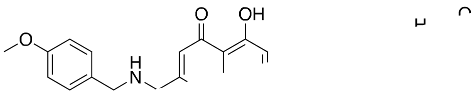	6.76± 0.39	10.1021/jm070336k
Inhibitor **7**	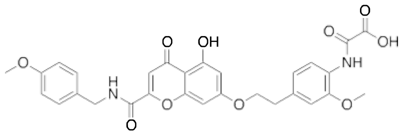	2.66± 0.24	10.1021/jm070336k
Gossypol	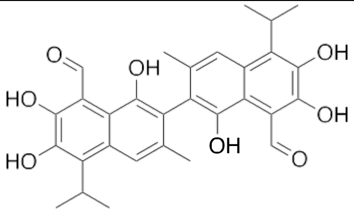	2.03 ± 0.80	10.1021/jm070336k

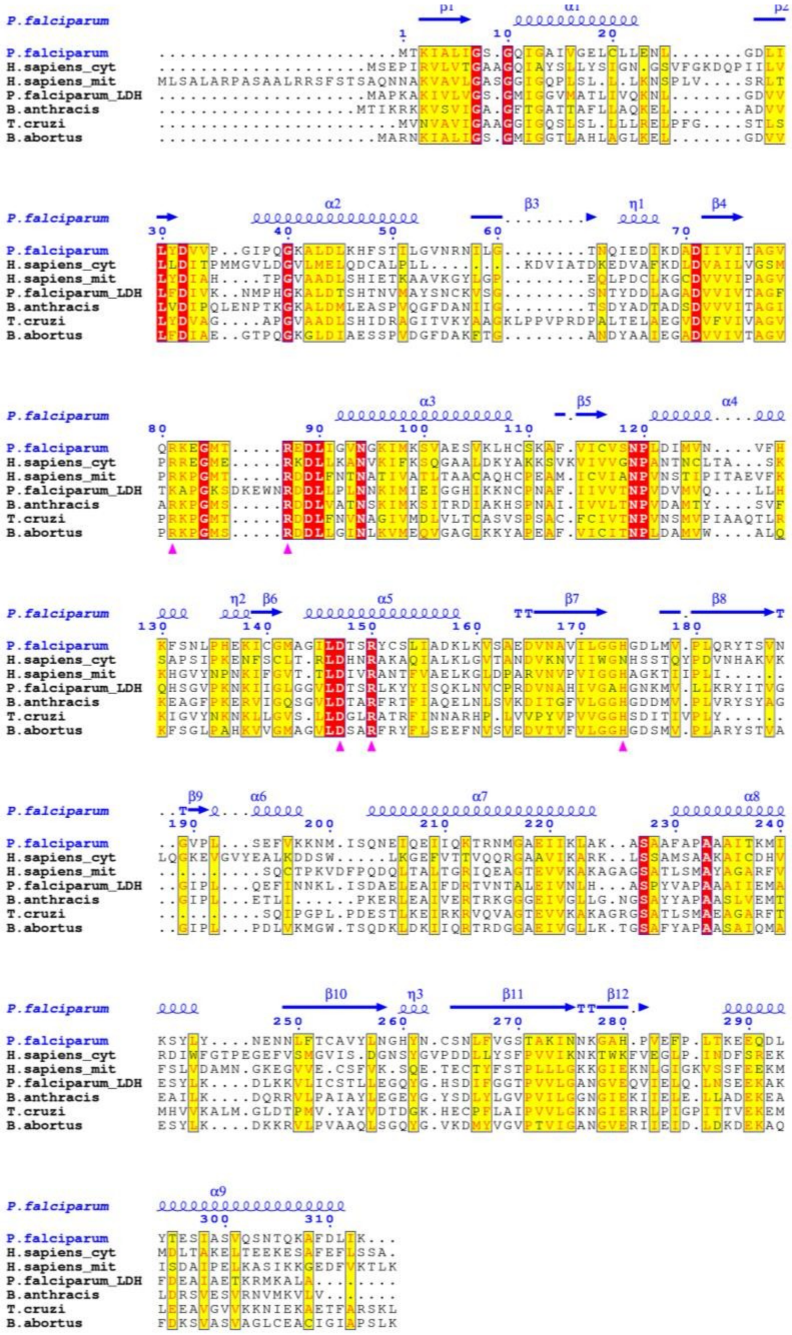

Supplementary Fig. 1. ClustalW multiple sequence alignment of four pathogenic microorganisms and human MDHs with *P. falciparum* set as reference (blue colored). The aminoacids involved in the catalysis are indicated with a purple triangle. Strictly and slightly conserved residues are respectively boxed in red and yellow. Characters of residues with similar physiochemical properties are colored in red characters. Secondary structures were created with ESPript[1]. Primary sequences were fetched from UniProtKB database under the accession IDs Q6VVP7 (*Plasmodium falciparum*), Q27743 (*Plasmodium falciparum*, LDH), P40925 (*Homo sapiens*, cytoplasmic), P40926 (*Homo sapiens*, mitochondrial), C3PAI1 (*Bacillus anthracis*), A0A2V2WGI8 (*Trypanosoma cruzi*) and Q2YLR9 (*Brucella abortus*).

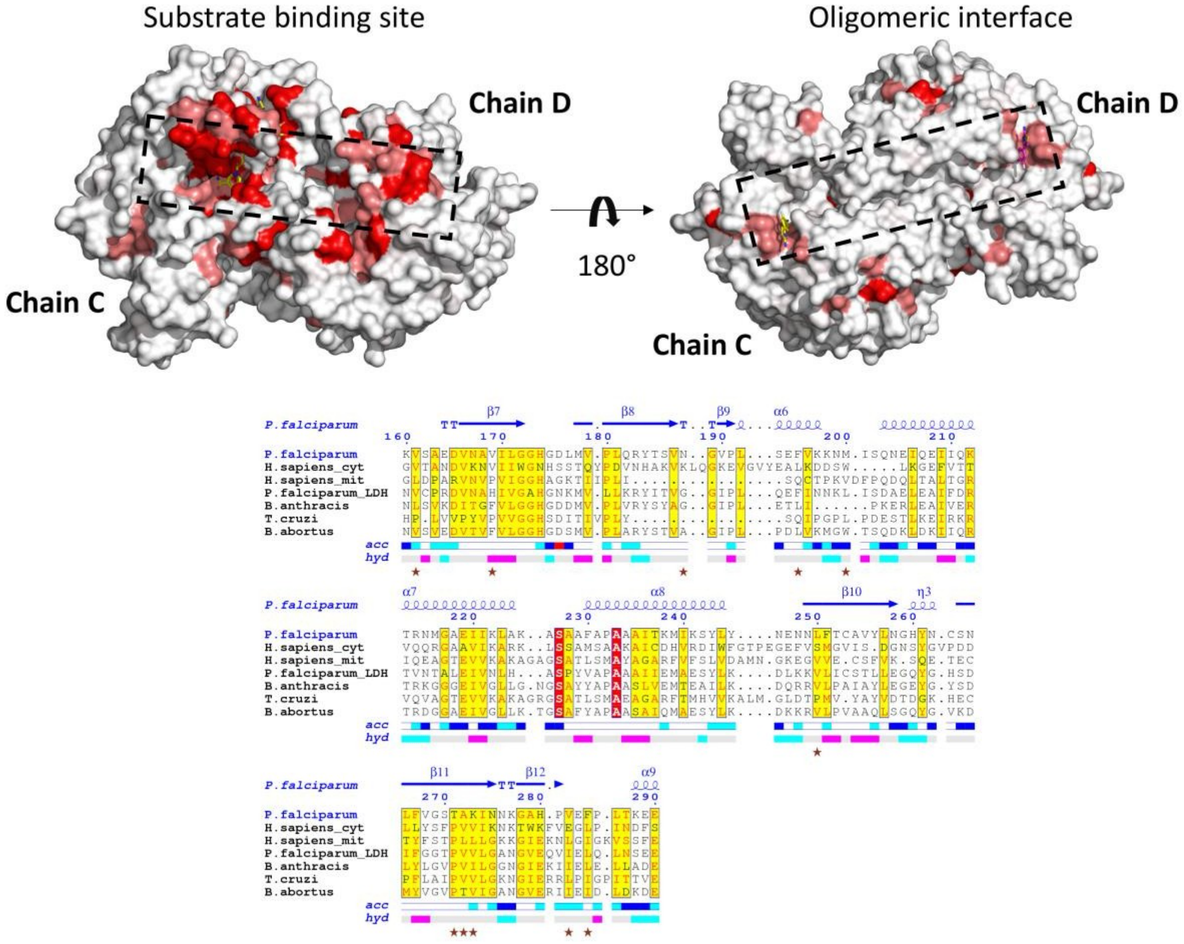

Supplementary Fig. 2. Upper part: surface representation of the *Pf*MDH with NADH and 4-(3,4-difluorophenyl) thiazol-2-amine displayed as sticks. Residues with similarity global score lower than 0.7 are white colored while those in the range from 0.7 to 1.0 (1=strictly conserved residues) in salmon or red. Lower part: ClustalW Multiple sequence alignment with same representation as described in **Supplementary Fig. 1**. Brown stars indicate the aminoacids interacting with 4-(3,4-difluorophenyl) thiazol-2-amine through Van der Walls or π-stacking contacts. “acc”: solvent accessibility of each residue (blue=accessible, white=buried, cyan=intermediate); “hyd”: hydropathic character of a sequence (pink=hydrophobic, grey=intermediate, cyan=hydrophilic).

Supplementary Table 3. ANOVA summary for the activity screening of the 4DT derivatives.

F	10.40
P value	<0.001
P value summary	***
Significant diff. among means (P < 0.05)?	Yes
R squared	0.9044
ANOVA summary	
F	10.40
P value	<0.001

Supplementary Table 4. Pairwise p-value calculation between the treated group with the molecule and the positive control (DMSO).

**Dunnett's multiple comparisons test**	**Adjusted p-value**
Positive control vs. **1a**	0.02
Positive control vs. **4a**	0.03
Positive control vs. **5a**	0.003
Positive control vs. **7a**	< 0.001
Positive control vs. **9a**	0.003
Positive control vs. **1b**	0.01
Positive control vs. **2b**	0.03
Positive control vs. **4b**	< 0.001
Positive control vs. **5b**	0.004
Positive control vs. **6b**	0.33

Supplementary Table 5. Table of results of 4PA dose response experiments.

**log(inhibitor) vs. normalized response -- Variable slope**	
**Best-fit values**	
LogIC50	-2.934
HillSlope	-3.652
IC50	0.001165
**95% CI (profile likelihood)**	
LogIC50	-2.997 to -2.876
HillSlope	-6.563 to -2.314
IC50	0.001007 to 0.001331
**Goodness of Fit**	
Degrees of Freedom	22
R squared	0.8525
Sum of Squares	5010
Sy.x	15.09
**Number of points**	
# of X values	24
# Y values analyzed	24

Supplementary Table 6. Parameters for the substrate inhibition model. Vmax, Km and Ki are presented as mean ± SEM of three independent experiments.

	**Control**	**63 μM**	**125 μM**
Best-fit values (pH 10.2)			
Vmax	967.8 ± 99.0	645.7 ± 29.0	630.2 ± 28.5
Km	0.4 ± 0.1	0.2 ± 0.003	0.2 ± 0.03
Ki	12.5 ± 4.2	63.00 ± 31.8	72.93 ± 41.9
95% CI (profile likelihood)			
Vmax	790.8 to 1255	588.4 to 713.5	574.1 to 696.8
Km	0.2438 to 0.7037	0.1395 to 0.2614	0.1378 to 0.2595
Ki	6.209 to 29.59	28.66 to 1374	30.76 to +infinity
Goodness of Fit			
Degrees of Freedom	20	20	20
R squared	0.8779	0.9290	0.9291
Sum of Squares	108093	31909	31624
Sy.x	73.52	39.94	39.76
Constraints			
Km	Km > 0	Km > 0	Km > 0
Ki	Ki > 0	Ki > 0	Ki > 0
Number of points			
# of X values	24	24	24
# Y values analyzed	23	23	23

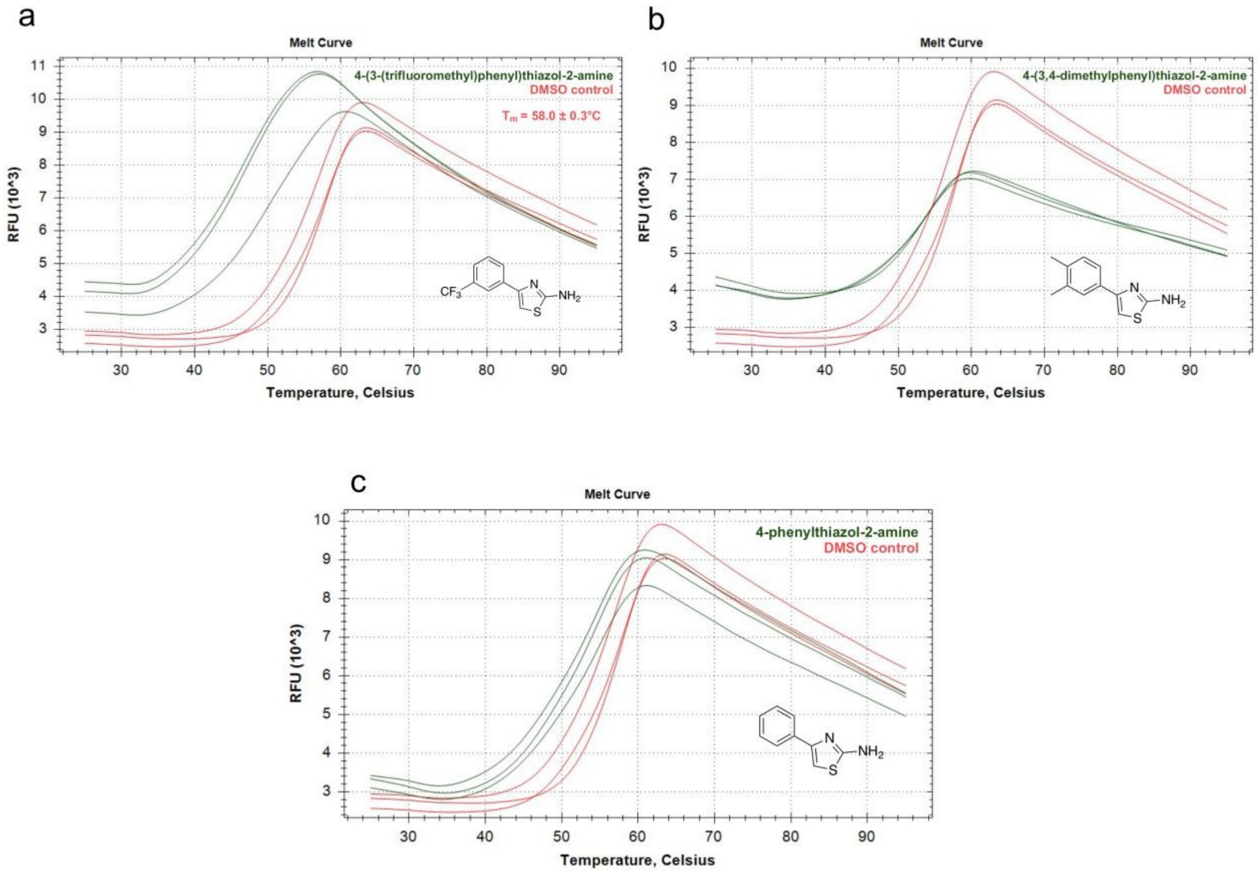

Supplementary Fig. 3. First derivative curves of TSA experiments. Samples wells treated with (**A**) 4-(3-(trifluoromethyl)phenyl)thiazol-2-amine (**B**) 4-(3,4 dimethyl phenylthiazol-2-amine (compound **5a**), (**C**) 4-phenylthiazole-2-amine are colored in green while the DMSO control group in red.

Supplementary Table 7. Statistics summary of TSA experiments.

Dunn's multiple comparisons test	Mean rank diff.	Significant?	Summary	Adjusted P Value
Control vs. 4-phenylthiazole-2-amine	6.333	No	ns	0.32
Control vs. Column B	8.667	No	ns	0.07
Control vs. Column C	12	Yes	**	0.004
Control vs. Column E	3	No	ns	>0.99

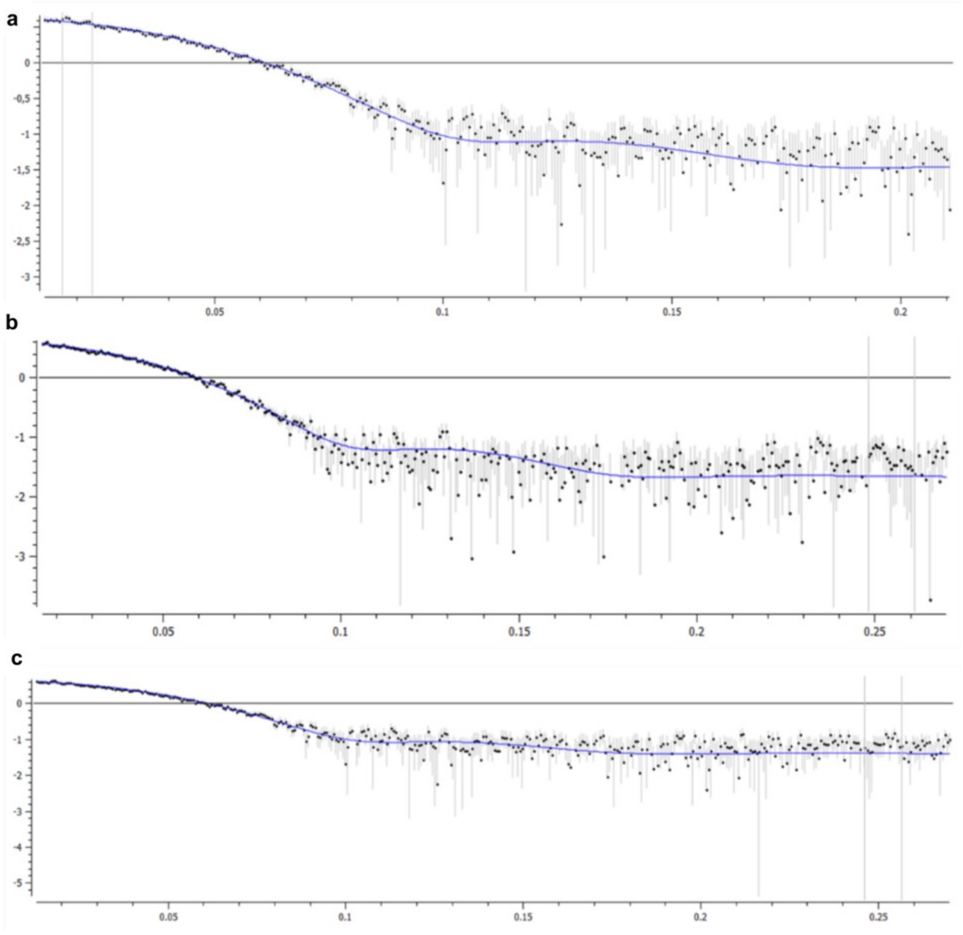

Supplementary Fig. 4. Comparison of goodness-of-fit between experimental SAXS curve of (**a**) apo ***Pf*MDH**, (**b**) in complex with inhibitor **2a**, (**c**) in complex with inhibitor **6a** with putative scattering curves from crystal structures generated using Crysol software.

Supplementary Table 8. Overview of SAXS parameters for apo *Pf*MDH dehydrogenase and with complex with compound 2a and compound 6a**.**

	R_g [Å]	R_g from D_{max} analysis [Å]	D_{max} [Å]	MW estimate [kDa]	credibility interval [kDa]
**apo**	33.78 ± 5.51	33.93 ± 4	104.75	113.65	106.9 - 121.45
**2a**	33.78 ± 0.83	33.93 ± 4	104.75	113.65	106.9 - 121.45
**6a**	36.4 ± 3.15	34.52 ± 4.3	113.74	91.175	81.3 - 95.8

Supplementary Methods 1: Docking of 4DT in *Hs*MDH1 and *Hs*MDH2

In order to better understand the potential binding of 4DT to different malate dehydrogenases, the molecule was docked on human *Hs*MDH1 and *Hs*MDH2. Since no crystal structure of *Hs*MDH1 has been deposited in PDB, we have built a homology model from its primary sequence. Based on our results, 4DT does not bind to the oligomeric interface in either of the two receptors studied. Besides, the calculated cooperativity scores of 3.6 (*Hs*MDH1, lower part of **Supplementary Fig. 6**) and 5.2 (*Hs*MDH2, upper part of **Supplementary Fig. 6**) indicate less opportunity to form interactions significant a binding affinity stronger than that of the allosteric pocket of *Pf*MDH (9.5). As for *Hs*MDH2, these interactions consist of two hydrogen bonds with the CO group side chain of Q38 and the OH group of T248 from chain B. The benzene ring forms a dipolar contact with G35 from chain A. Finally, several Van der Waals interactions between the two fluoride atoms and the hydrophobic residues L249, P39 of each chain were observed and, similarly, the thiazole ring forms two van Der Waals contacts with G35. As for *Hs*MDH1, 4DT forms nine Van der Waals contacts with hydrophobic residues A61 from chain B and A165, A168 and L169 from chain A. Furthermore, Q58 and G172 interact with the fluoride atom in meta position of the benzene and the thiazole rings through two Van Der Walls contacts. In addition, G172 forms one hydrogen bond – π stacking interaction with the amino group. Finally, one π stacking interaction was observed between the backbone NH group of L169 and the benzene. In conclusion, these data suggest that in both human isoforms 4DT binds in a binding pocket vicinal to the NAD cofactor-binding domain. While no 4DT docking to the determined allosteric site could be observed through our modeling, this could also imply that further experiments could be required to investigate a potential off-target effect for *Hs*MDH2 rather than for *Hs*MDH1.

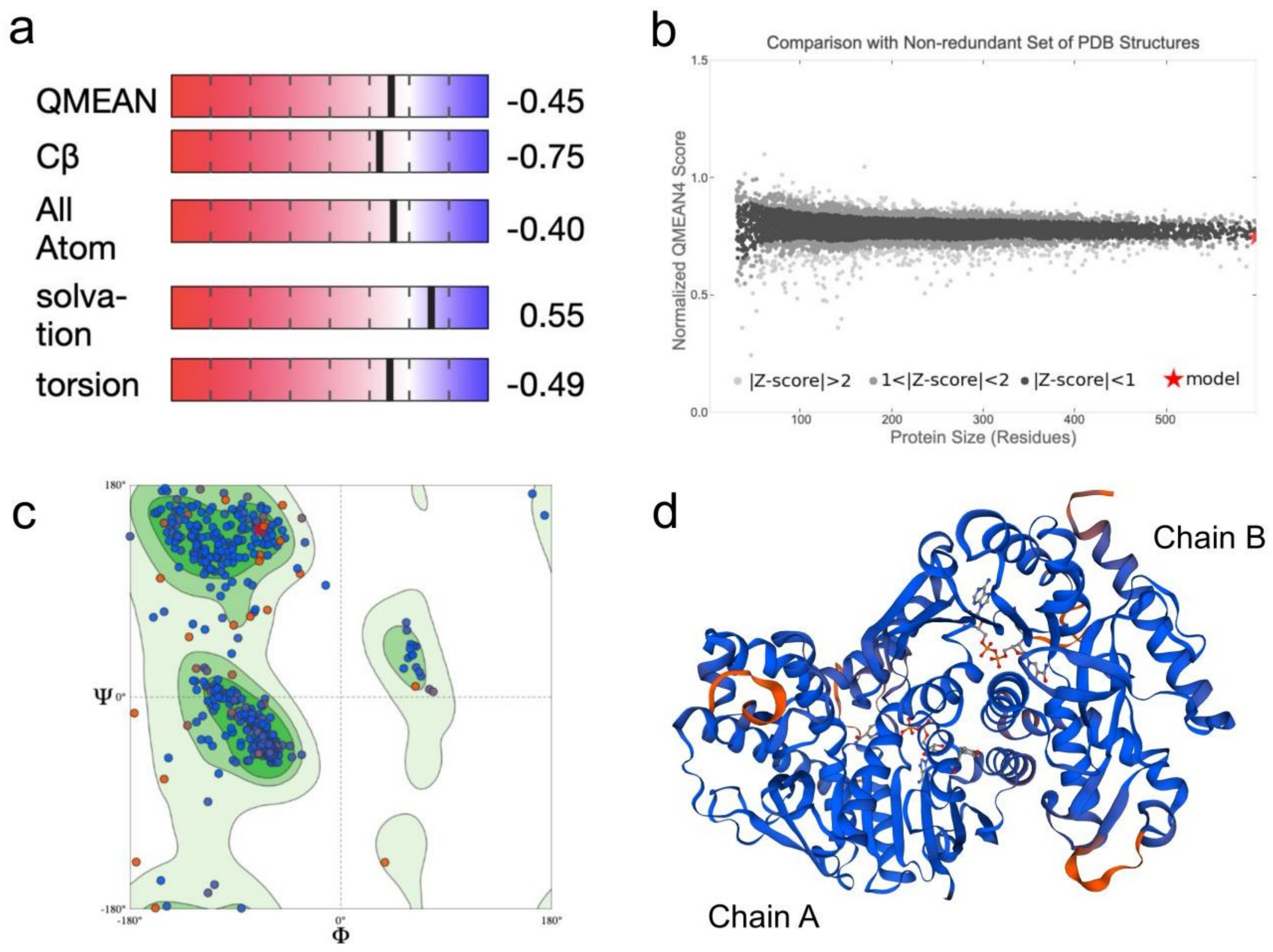

Supplementary Fig. 5. Homology modeling report for *Hs*MDH1. (**A**) The four principal individual terms QMEAN scoring function. White, blue and red areas indicate properties being on average equal, better or worse than the reference structure. (**B**) Comparison plot of *Hs*MDH1 model quality scores (red star) versus proteins of similar sizes. Black dots are crystal structures with normalized QMEAN score (0<Z-score<1) while those ones with Z-score even further are grey colored. (**C**) Ramachandran plot showing the energetically favored regions where 80% (third contour), 95% (second contour) and 99.7% (first contour) of dihedral angle combinations fall. (**D**) Cartoon representation of the homomeric form of *Hs*MDH1. Poorly modelled structures are coloured red and those with positive QMEAN in blue.

Supplementary Methods 2: Homology model: validation and evaluation

Templates were searched with BLAST [2] across the SWISS-MODEL template library using the primary sequence of MDH1 (Uniprot ID: P40925). Templates to build up the models were chosen based on a sequence identity cut-off of 30% and the reported oligomeric state of *Hs*MDH1 in presence of NAD. Next, the model was built with ProMod3 algorithm[3] which replaces missing sides chains atoms with a fragment library while loops modelling has been performed with PROMOD-II. The global and per-residue model quality has been assessed using the QMEAN scoring function.[4] Finally, the model was validated using Ramachandran plot which quantifies the number of outliers and clashing contacts. With a sequence identity of 94.26% with the template (pig heart MDH1, PDB: 4MDH), a global QMEAN of -0.45 and a *Z*-score <1, the model was judged to be suitable for being used as a receptor (**Supplementary Fig. 5A** and **Supplementary Fig. 5B**). However, the regions comprising the amino acids from K201 to V207 and active site loop R92 – L102 presented poor-quality scores due to the lack of electron density in the template (**Supplementary Fig. 5D**). Finally, as can be seen in **Supplementary Fig. 5C**, with all Ramachandran outliers located outside the 4DT binding site and 0.13 Å RMSD between the experimental crystal structure and the crude model itself, we felt confident to proceed with the docking analysis.

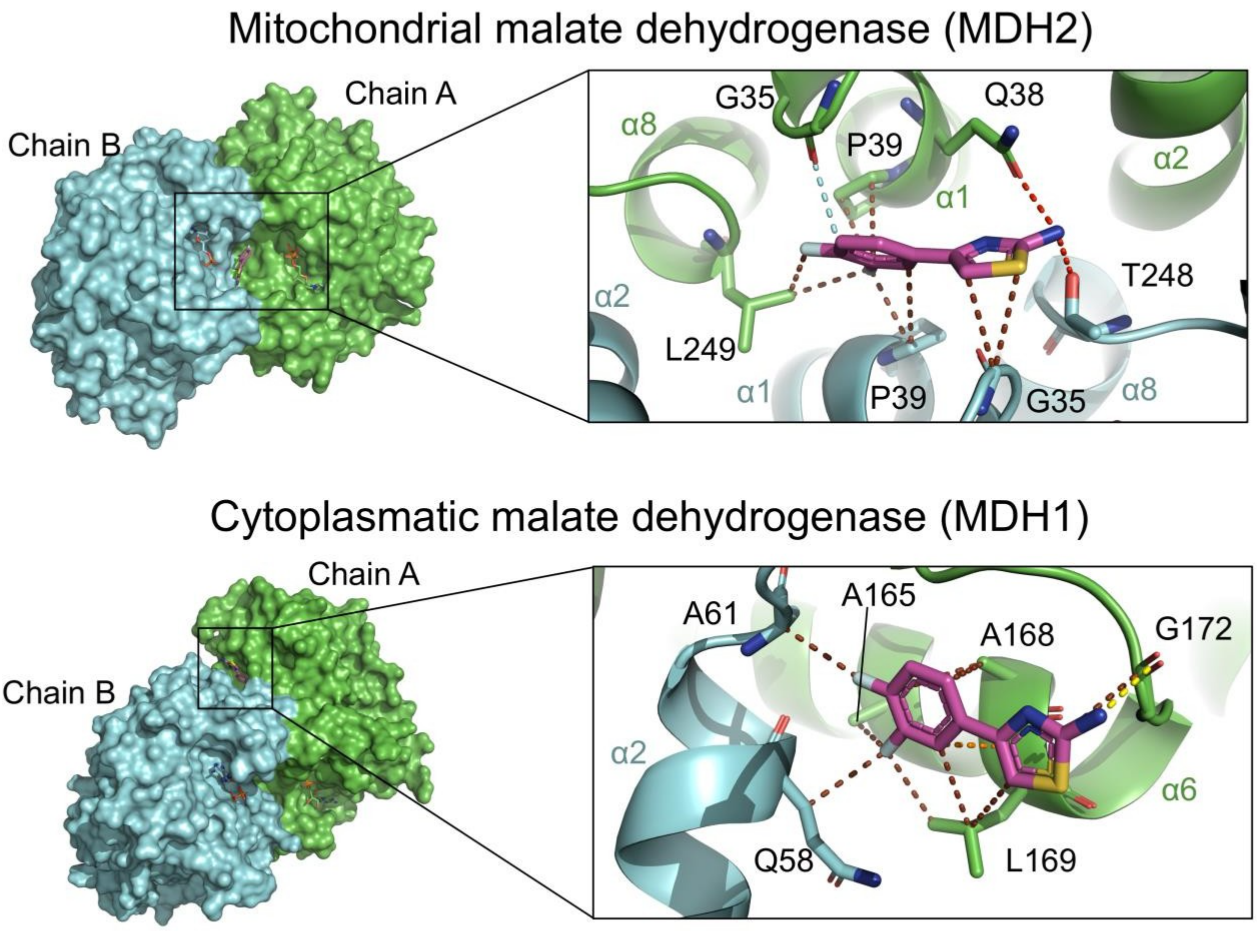

Supplementary Fig. 6. Surface representation of *Hs*MDH2 and *Hs*MDH1 and close-up views of the docked 4DT (stick representation) as well as its molecular interactions. Hydrogen bond – π, van der Waals, hydrogen bonding, π stacking and dipolar interactions are shown in yellow, brown, red, orange and cyan dotted lines respectively.

Supplementary References

1. Gouet, P., Robert, X. & Courcelle, E. ESPript/ENDscript: Extracting and rendering sequence and 3D information from atomic structures of proteins. *Nucleic Acids Res.* **31**, 3320–3323 (2003).
2. Biasini, M., T. Schmidt, S. Bienert, V. Mariani, G. Studer, J. Haas, N. Johner, A. D. Schenk, A. Philippsen, e T. Schwede. OpenStructure: An Integrated Software Framework for Computational Structural Biology. *Acta Crystallographica. Section D, Biological Crystallography* **69**, 701–709 (2013).
3. Camacho, Christiam, George Coulouris, Vahram Avagyan, Ning Ma, Jason Papadopoulos, Kevin Bealer, e Thomas L. Madden. BLAST+: Architecture and Applications. *BMC Bioinformatics* **10**, 1–9 (2009).
4. Studer, Gabriel, Christine Rempfer, Andrew M. Waterhouse, Rafal Gumienny, Juergen Haas, e Torsten Schwede. QMEANDisCo—Distance Constraints Applied on Model Quality Estimation. *Bioinformatics* **36**, 1765–1771 (2020).
