# Supplementary material for: A fragment-based approach identifies an allosteric pocket that impacts malate dehydrogenase activity
Source: Commun Biol. 2021 Aug 10;4:949. doi: 10.1038/s42003-021-02442-1 (PMC8355244; doi:10.1038/s42003-021-02442-1)
Supplement: Supplementary file 3 — Description of Additional Supplementary Files [file 42003_2021_2442_MOESM3_ESM.pdf]

Description of Additional Supplementary Files

File name: Supplementary Data 1

Description: MT data processing

File name: Supplementary Data 2

Description: Raw Data used for Figure 1

File name: Supplementary Data 3

Description: PISA alignment and interfaces analysis

File name: Supplementary Data 4

Description: Thermal shift assay raw data

File name: Supplementary Data 5

Description: Validation report for Crystal structure of malate dehydrogenase from Plasmodium Falciparum incomplex with 4-(3,4-difluorophenyl)thiazol-2-amine (PDB ID: 6R8G).

File name: Supplementary Data 6

Description: Validation report for Crystal structure of malate dehydrogenase from Plasmodium Falciparum in complex with NADH (PDB ID: 6Y91).

File name: Supplementary Data 7

Description: Statistics and data processing for 4DT derivatives.

File name: Supplementary Data 8

Description: Statistics and data processing for 4PA dose response experiment.

File name: Supplementary Data 9

Description: Statistics and data processing for 4PA kinetics experiments.

File name: Supplementary Data 10

Description: C-terminal His-tagged PfMDH WT sequencing data and primary sequence.
