# Supplementary material for: A fragment-based approach identifies an allosteric pocket that impacts malate dehydrogenase activity
Source: Commun Biol. 2021 Aug 10;4:949. doi: 10.1038/s42003-021-02442-1 (PMC8355244; doi:10.1038/s42003-021-02442-1)
Supplement: Supplementary file 4 — Supplementary Data 1 [file 42003_2021_2442_MOESM4_ESM.pdf]

Analysis Report

Analysis Name:	HMGU21_MST triplciate
Type of Analysis:	MST
Evaluation strategy:	Manual
Cold Region Start:	-1 s
Cold Region End:	0 s
Hot Region Start:	0.5 s
Hot Region End:	1.5 s
Exported on:	Tue, 17 Sep 2019 14:08:41 GMT
File:	Atilio_Holak group.nta
Software Version:	MO.Affinity Analysis v2.3

Compare Kd-Fit: HMGU21_MST triplciate

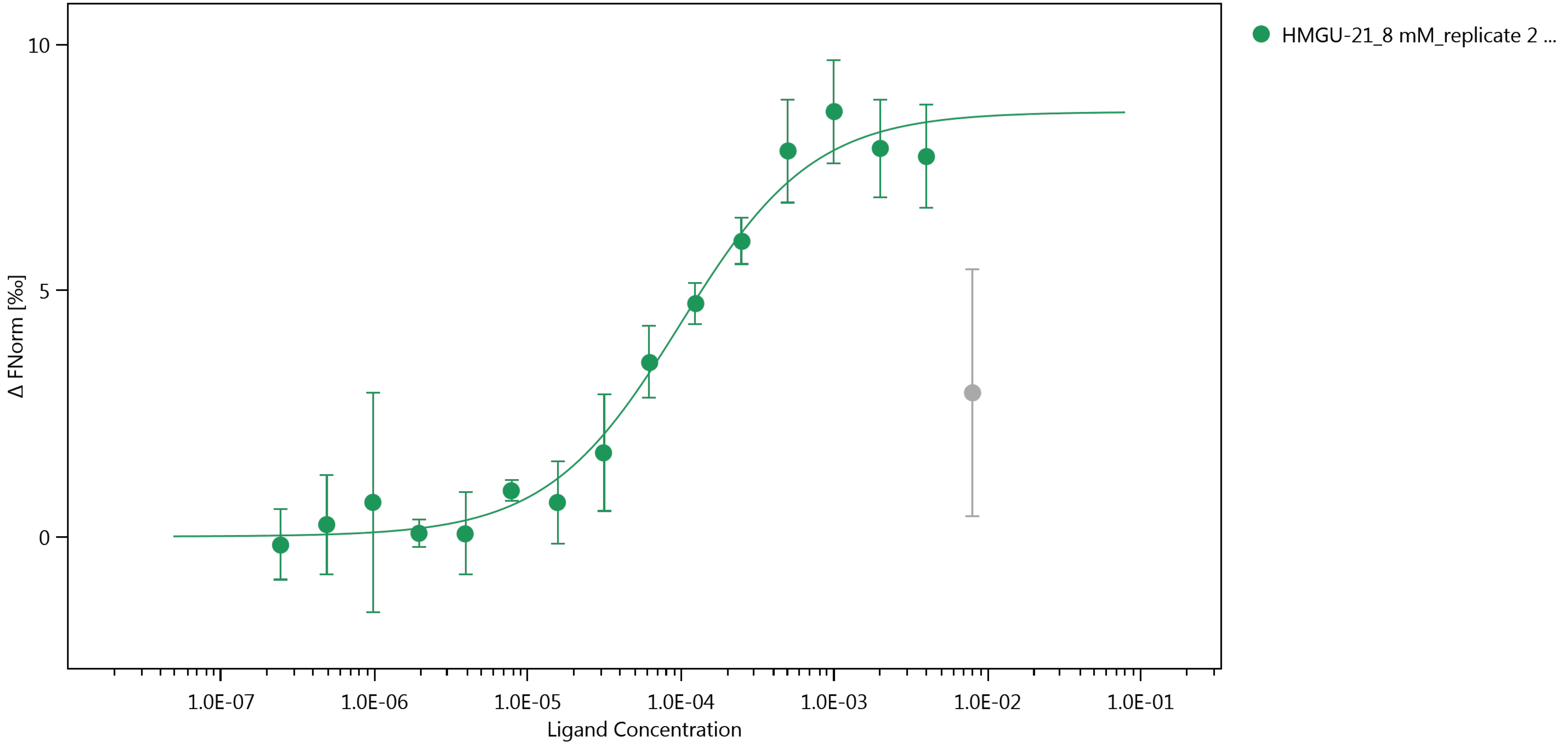

Dataset Overview

Name:	HMGU-21_8 mM_replicate 2 (#15)
Graph Color:	●
Target Name:	PfMDH
Target Concentration:	50 nM
Ligand Name:	hmgu21
Ligand Concentration:	4 mM to 0.000244 mM
n:	3
Comments:	
Excitation Power:	40%
MST Power:	40%
Temperature:	25.1°C
Kd:	9.9016E-05
Kd Confidence:	± 1.7444E-05
Response Amplitude:	8.6229515
TargetConc:	5E-08[Fixed]
Unbound:	951.63
Bound:	960.25
Std. Error of Regression:	0.47292197
Reduced χ^2:	0.39078245
Signal to Noise:	19.694268

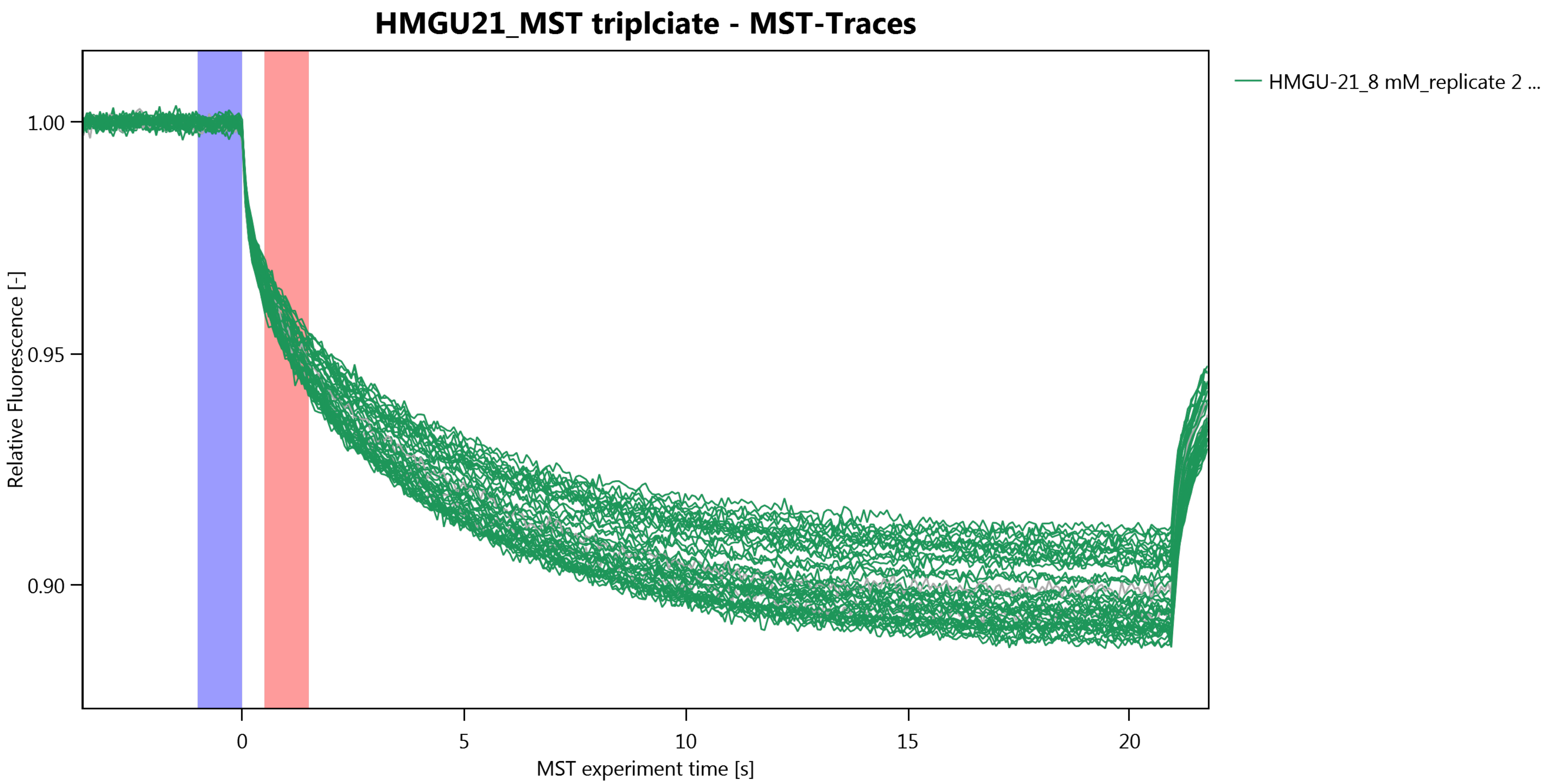
HMGU21_MST triplciate - MST-Traces
Relative Fluorescence [-]
1.00
0.95
0.90
0
5
10
15
20
MST experiment time [s]
HMGU-21_8 mM_replicate 2 ...

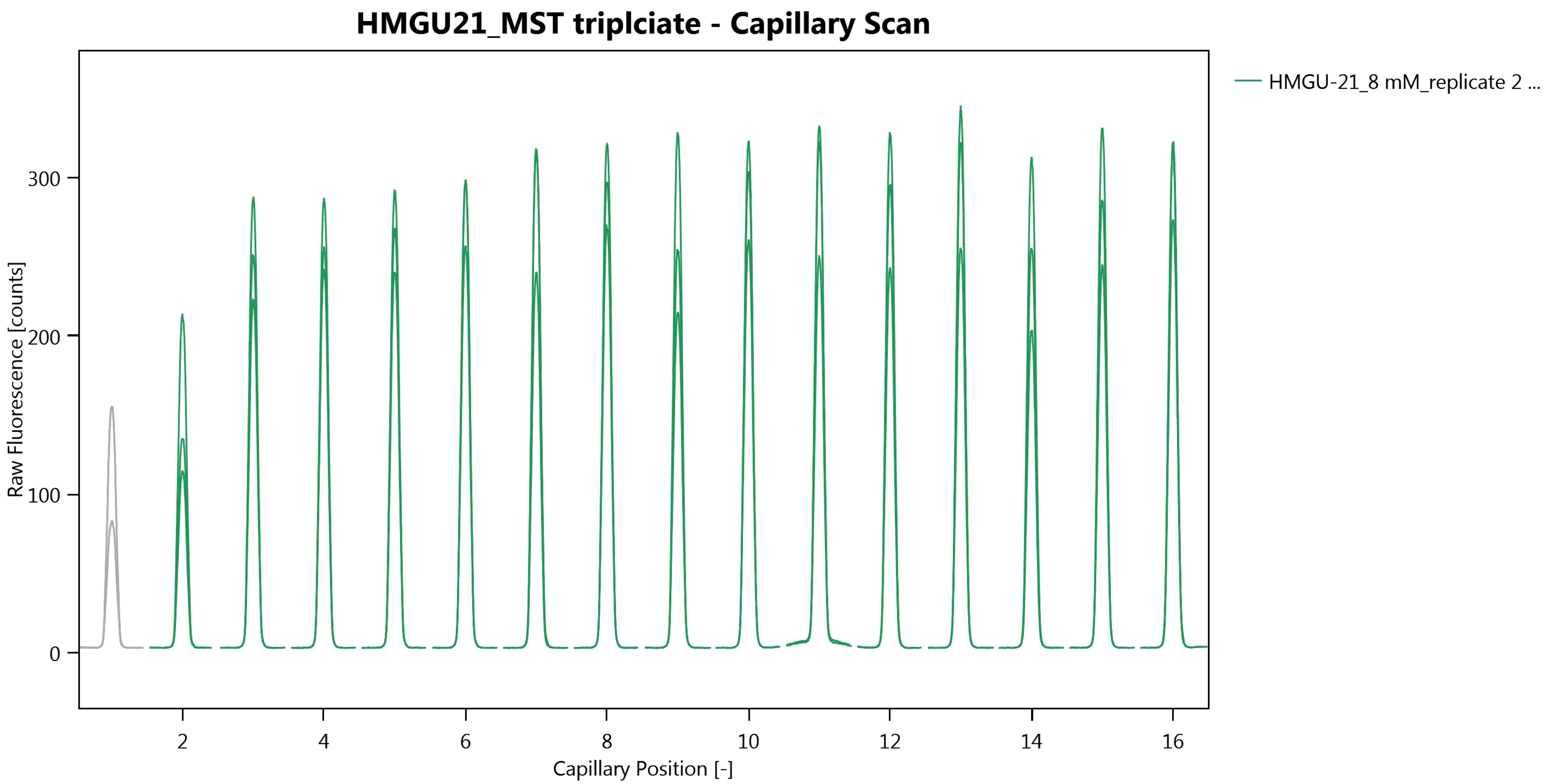
HMGU21_MST triplciate - Capillary Scan
HMGU-21_8 mM_replicate 2 ...
Raw Fluorescence [counts]
300
200
100
0
2
4
6
8
10
12
14
16
Capillary Position [-]

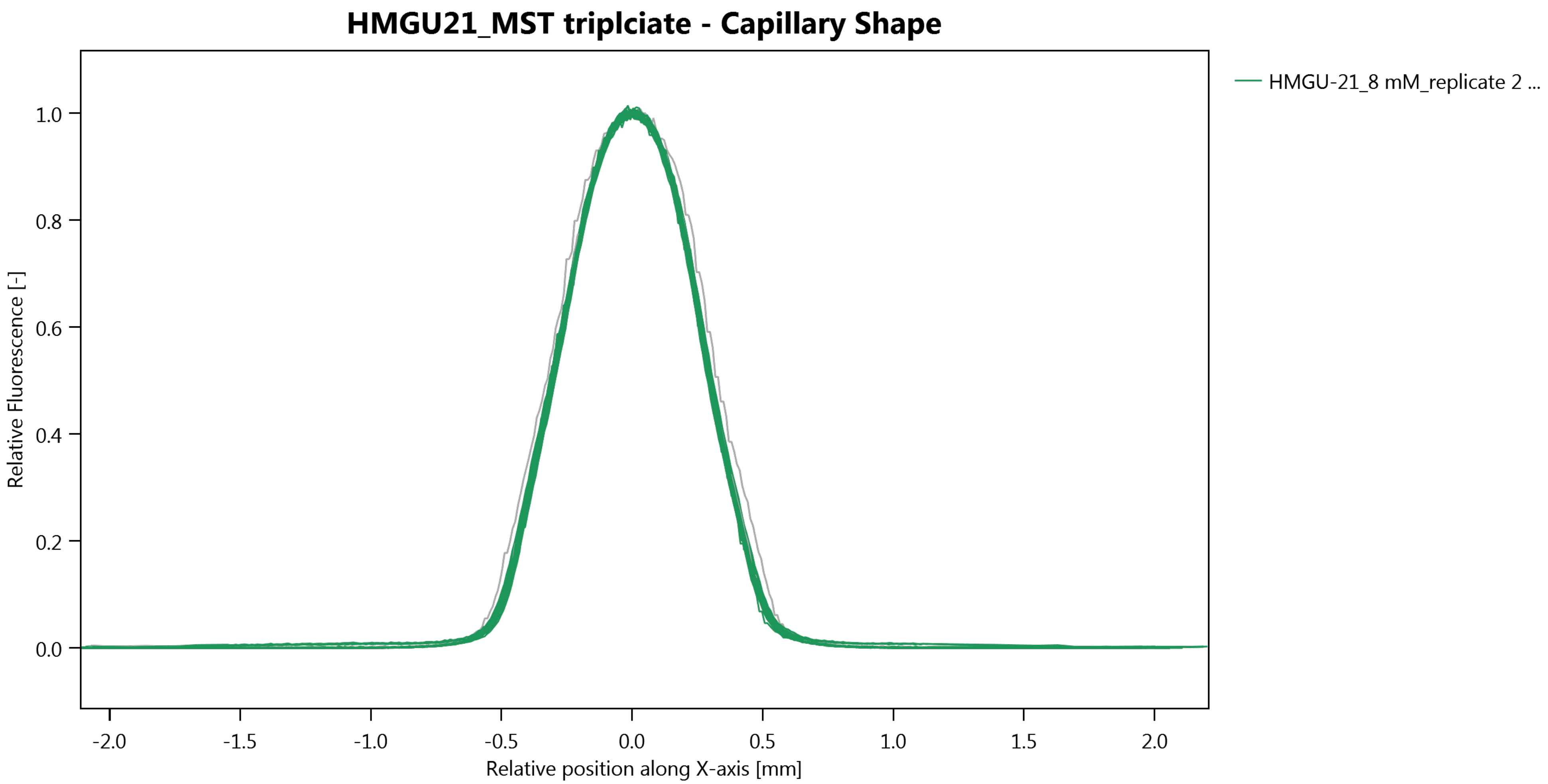
HMGU21_MST triplciate - Capillary Shape
HMGU-21_8 mM_replicate 2 ...
Relative Fluorescence [-]
1.0
0.8
0.6
0.4
0.2
0.0
-2.0
-1.5
-1.0
-0.5
0.0
0.5
1.0
1.5
2.0
Relative position along X-axis [mm]

Raw data of merged Dose-Response #1

HMGU-21_8 mM_replicate 2 (#15)

Dose	Response (Average)	Std. Dev.	n
0.004	959.34626	1.04824	3
0.002	959.51179	0.98371	3
0.001	960.25959	1.05606	3
0.0005	959.46053	1.032	3
0.00025	957.62435	0.4631	3
0.000125	956.36081	0.40345	3
6.25E-05	955.16319	0.73228	3
3.125E-05	953.33037	1.18821	3
1.5625E-05	952.32182	0.84449	3
7.8125E-06	952.55983	0.2075	3
3.90625E-06	951.68518	0.83507	3
1.953125E-06	951.69583	0.2672	3
9.765625E-07	952.32357	2.24233	3
4.8828125E-07	951.87281	1.01078	3
2.4414063E-07	951.45794	0.71092	3

Outlier

Dose	Response	Std. Dev.	Number of merged Points
0.008	954.54927	2.4918077	0
