# Supplementary material for: A fragment-based approach identifies an allosteric pocket that impacts malate dehydrogenase activity
Source: Commun Biol. 2021 Aug 10;4:949. doi: 10.1038/s42003-021-02442-1 (PMC8355244; doi:10.1038/s42003-021-02442-1)
Supplement: Supplementary file 8 — Supplementary Data 5 [file 42003_2021_2442_MOESM8_ESM.pdf]

Full wwPDB X-ray Structure Validation Report (i)

May 25, 2020 – 11:57 am BST

PDB ID	:	6R8G
Title	:	Crystal structure of malate dehydrogenase from Plasmodium Falciparum in complex with 4-(3,4-difluorophenyl)thiazol-2-amine
Authors	:	Romero, A.R.; Calderone, V.; Gentili, M.; Lunev, S.; Groves, M.; Popowicz, G.; Domling, A.; Sattler, M.
Deposited on	:	2019-04-01
Resolution	:	2.00 Å(reported)

This is a Full wwPDB X-ray Structure Validation Report for a publicly released PDB entry.

We welcome your comments at *validation@mail.wwpdb.org*
A user guide is available at
https://www.wwpdb.org/validation/2017/XrayValidationReportHelp
with specific help available everywhere you see the (i) symbol.

The following versions of software and data (see references (i)) were used in the production of this report:

MolProbity	:	4.02b-467
Mogul	:	1.8.5 (274361), CSD as541be (2020)
Xtriage (Phenix)	:	1.13
EDS	:	2.11
buster-report	:	1.1.7 (2018)
Percentile statistics	:	20191225.v01 (using entries in the PDB archive December 25th 2019)
Refmac	:	5.8.0158
CCP4	:	7.0.044 (Gargrove)
Ideal geometry (proteins)	:	Engh & Huber (2001)
Ideal geometry (DNA, RNA)	:	Parkinson et al. (1996)
Validation Pipeline (wwPDB-VP)	:	2.11

1 Overall quality at a glance (i)

The following experimental techniques were used to determine the structure:
X-RAY DIFFRACTION

The reported resolution of this entry is 2.00 Å.

Percentile scores (ranging between 0-100) for global validation metrics of the entry are shown in the following graphic. The table shows the number of entries on which the scores are based.

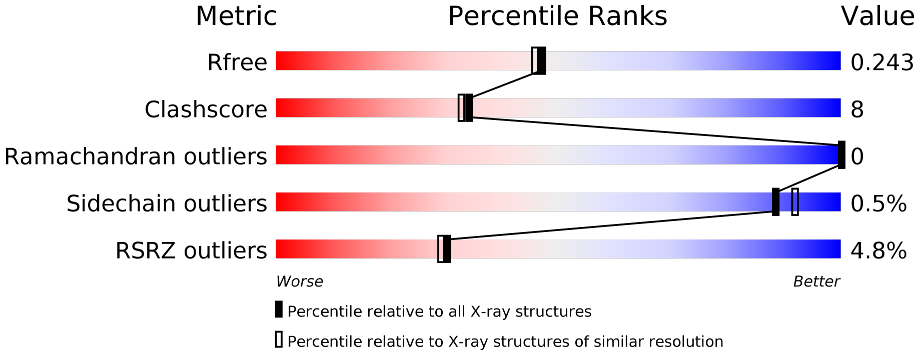

Metric	Whole archive (#Entries)	Similar resolution (#Entries, resolution range(Å))
R_{free}	130704	8085 (2.00-2.00)
Clashscore	141614	9178 (2.00-2.00)
Ramachandran outliers	138981	9054 (2.00-2.00)
Sidechain outliers	138945	9053 (2.00-2.00)
RSRZ outliers	127900	7900 (2.00-2.00)

The table below summarises the geometric issues observed across the polymeric chains and their fit to the electron density. The red, orange, yellow and green segments on the lower bar indicate the fraction of residues that contain outliers for >=3, 2, 1 and 0 types of geometric quality criteria respectively. A grey segment represents the fraction of residues that are not modelled. The numeric value for each fraction is indicated below the corresponding segment, with a dot representing fractions <=5% The upper red bar (where present) indicates the fraction of residues that have poor fit to the electron density. The numeric value is given above the bar.

Mol	Chain	Length	Quality of chain
1	A	324	9% / 81% 15% • •
1	B	324	6% / 84% 11% • •
1	C	324	2% / 86% 10% •
1	D	324	% / 86% 9% • •

The following table lists non-polymeric compounds, carbohydrate monomers and non-standard residues in protein, DNA, RNA chains that are outliers for geometric or electron-density-fit crite-

ria:

Mol	Type	Chain	Res	Chirality	Geometry	Clashes	Electron density
2	JUT	C	402	-	X	X	-
2	JUT	D	402	-	X	-	-

2 Entry composition (i)

There are 5 unique types of molecules in this entry. The entry contains 9848 atoms, of which 0 are hydrogens and 0 are deuteriums.

In the tables below, the ZeroOcc column contains the number of atoms modelled with zero occupancy, the AltConf column contains the number of residues with at least one atom in alternate conformation and the Trace column contains the number of residues modelled with at most 2 atoms.

- Molecule 1 is a protein called Malate dehydrogenase.

Mol	Chain	Residues	Atoms					ZeroOcc	AltConf	Trace
			Total	C	N	O	S			
1	A	311	2366	1503	400	448	15	0	0	0
1	B	312	2375	1509	402	449	15	0	0	0
1	C	312	2375	1509	402	449	15	0	0	0
1	D	311	2366	1503	400	448	15	0	0	0

There are 44 discrepancies between the modelled and reference sequences:

Chain	Residue	Modelled	Actual	Comment	Reference
A	314	ALA	-	expression tag	UNP Q6VVP7
A	315	ALA	-	expression tag	UNP Q6VVP7
A	316	ALA	-	expression tag	UNP Q6VVP7
A	317	LEU	-	expression tag	UNP Q6VVP7
A	318	GLU	-	expression tag	UNP Q6VVP7
A	319	HIS	-	expression tag	UNP Q6VVP7
A	320	HIS	-	expression tag	UNP Q6VVP7
A	321	HIS	-	expression tag	UNP Q6VVP7
A	322	HIS	-	expression tag	UNP Q6VVP7
A	323	HIS	-	expression tag	UNP Q6VVP7
A	324	HIS	-	expression tag	UNP Q6VVP7
B	314	ALA	-	expression tag	UNP Q6VVP7
B	315	ALA	-	expression tag	UNP Q6VVP7
B	316	ALA	-	expression tag	UNP Q6VVP7
B	317	LEU	-	expression tag	UNP Q6VVP7
B	318	GLU	-	expression tag	UNP Q6VVP7
B	319	HIS	-	expression tag	UNP Q6VVP7
B	320	HIS	-	expression tag	UNP Q6VVP7
B	321	HIS	-	expression tag	UNP Q6VVP7
B	322	HIS	-	expression tag	UNP Q6VVP7
B	323	HIS	-	expression tag	UNP Q6VVP7

Continued on next page...

Continued from previous page...

Chain	Residue	Modelled	Actual	Comment	Reference
B	324	HIS	-	expression tag	UNP Q6VVP7
C	314	ALA	-	expression tag	UNP Q6VVP7
C	315	ALA	-	expression tag	UNP Q6VVP7
C	316	ALA	-	expression tag	UNP Q6VVP7
C	317	LEU	-	expression tag	UNP Q6VVP7
C	318	GLU	-	expression tag	UNP Q6VVP7
C	319	HIS	-	expression tag	UNP Q6VVP7
C	320	HIS	-	expression tag	UNP Q6VVP7
C	321	HIS	-	expression tag	UNP Q6VVP7
C	322	HIS	-	expression tag	UNP Q6VVP7
C	323	HIS	-	expression tag	UNP Q6VVP7
C	324	HIS	-	expression tag	UNP Q6VVP7
D	314	ALA	-	expression tag	UNP Q6VVP7
D	315	ALA	-	expression tag	UNP Q6VVP7
D	316	ALA	-	expression tag	UNP Q6VVP7
D	317	LEU	-	expression tag	UNP Q6VVP7
D	318	GLU	-	expression tag	UNP Q6VVP7
D	319	HIS	-	expression tag	UNP Q6VVP7
D	320	HIS	-	expression tag	UNP Q6VVP7
D	321	HIS	-	expression tag	UNP Q6VVP7
D	322	HIS	-	expression tag	UNP Q6VVP7
D	323	HIS	-	expression tag	UNP Q6VVP7
D	324	HIS	-	expression tag	UNP Q6VVP7

- Molecule 2 is 4-[3,4-bis(fluoranyl)phenyl]-1,3-thiazol-2-amine (three-letter code: JUT) (formula: $C_9H_6F_2N_2S$).

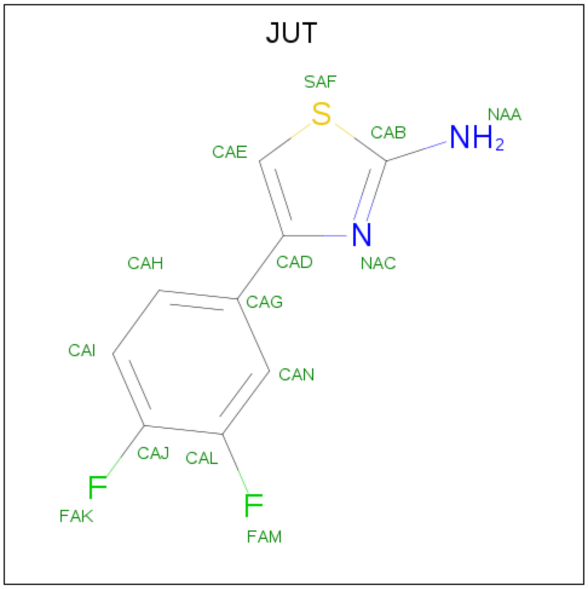

Mol	Chain	Residues	Atoms					ZeroOcc	AltConf
			Total	C	F	N	S		
2	A	1	14	9	2	2	1	0	0
2	C	1	14	9	2	2	1	0	0
2	D	1	14	9	2	2	1	0	0
2	D	1	14	9	2	2	1	0	0

- Molecule 3 is SODIUM ION (three-letter code: NA) (formula: Na).

Mol	Chain	Residues	Atoms		ZeroOcc	AltConf
			Total	Na		
3	A	1	1	1	0	0

- Molecule 4 is NICOTINAMIDE-ADENINE-DINUCLEOTIDE (three-letter code: NAD) (formula: $C_{21}H_{27}N_7O_{14}P_2$).

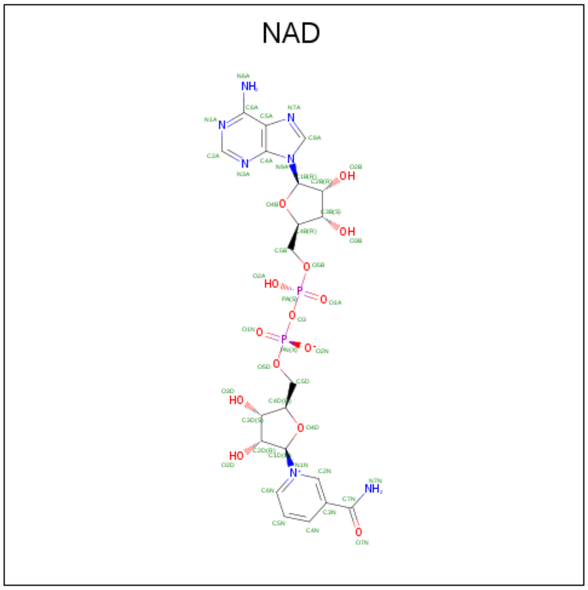

Mol	Chain	Residues	Atoms					ZeroOcc	AltConf
			Total	C	N	O	P		
4	C	1	44	21	7	14	2	0	0

- Molecule 5 is water.

Mol	Chain	Residues	Atoms		ZeroOcc	AltConf
			Total	O		
5	A	69	69	69	0	0

Continued on next page...

Continued from previous page...

Mol	Chain	Residues	Atoms	ZeroOcc	AltConf
5	B	56	Total O<br>56 56	0	0
5	C	59	Total O<br>59 59	0	0
5	D	81	Total O<br>81 81	0	0

3 Residue-property plots

These plots are drawn for all protein, RNA and DNA chains in the entry. The first graphic for a chain summarises the proportions of the various outlier classes displayed in the second graphic. The second graphic shows the sequence view annotated by issues in geometry and electron density. Residues are color-coded according to the number of geometric quality criteria for which they contain at least one outlier: green = 0, yellow = 1, orange = 2 and red = 3 or more. A red dot above a residue indicates a poor fit to the electron density (RSRZ > 2). Stretches of 2 or more consecutive residues without any outlier are shown as a green connector. Residues present in the sample, but not in the model, are shown in grey.

- Molecule 1: Malate dehydrogenase

Chain A:

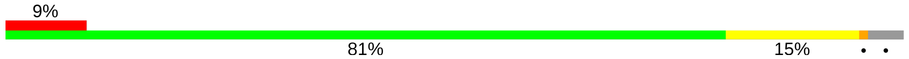

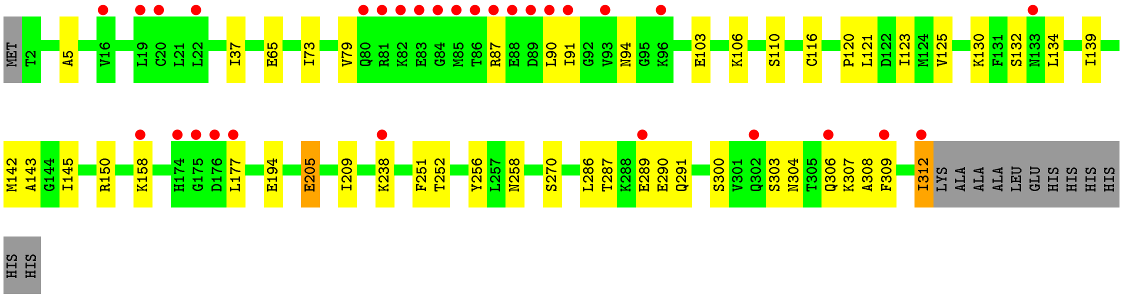

- Molecule 1: Malate dehydrogenase

Chain B:

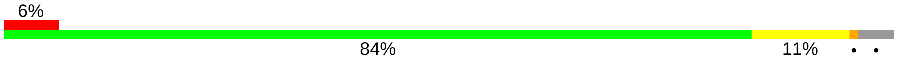

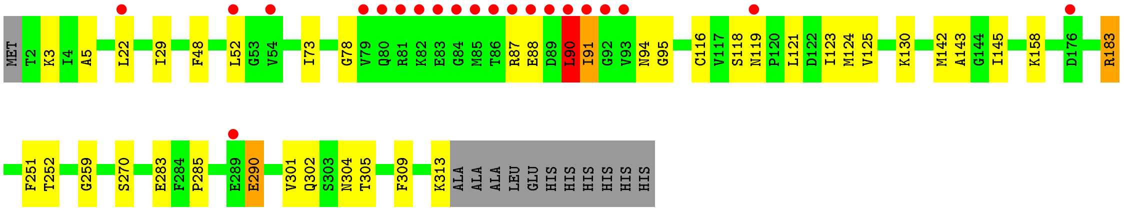

- Molecule 1: Malate dehydrogenase

Chain C:

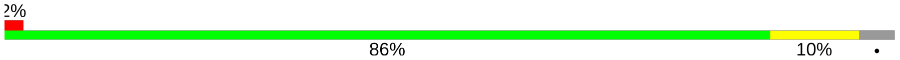

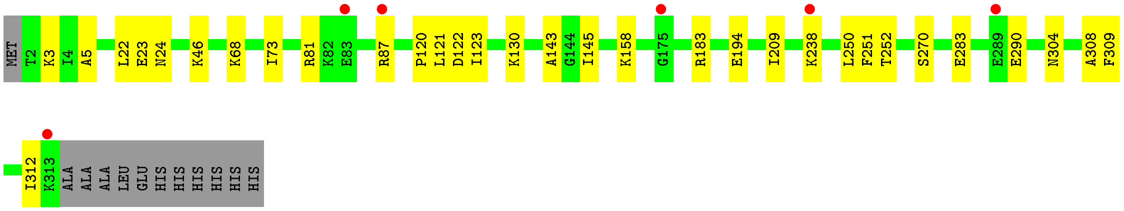

- Molecule 1: Malate dehydrogenase

Chain D:

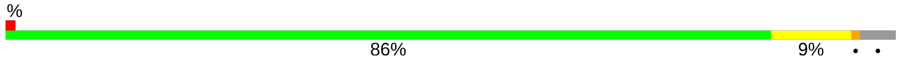

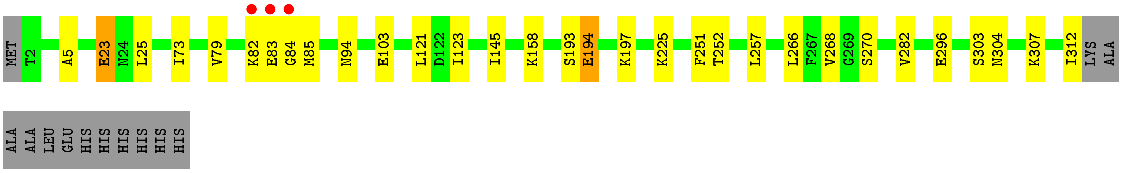
MET
T2
A5
E23
N24
L25
I73
V79
K82
E83
G84
M85
N94
E103
L121
D122
I123
I145
K158
S193
E194
K197
K225
F251
T252
L257
L266
F267
V268
G269
S270
V282
E296
S303
N304
K307
I312
LYS
ALA
ALA
ALA
LEU
GLU
HIS
HIS
HIS
HIS
HIS
HIS

4 Data and refinement statistics (i)

Property	Value	Source
Space group	P 21 21 21	Depositor
Cell constants<br>a, b, c, α, β, γ	84.76Å 106.89Å 145.01Å<br>90.00° 90.00° 90.00°	Depositor
Resolution (Å)	48.97 – 2.00<br>48.97 – 2.00	Depositor<br>EDS
% Data completeness<br>(in resolution range)	99.9 (48.97-2.00)<br>99.9 (48.97-2.00)	Depositor<br>EDS
R_{merge}	(Not available)	Depositor
R_{sym}	0.20	Depositor
$< I/\sigma(I) >$ [1]	1.02 (at 2.00Å)	Xtriage
Refinement program	REFMAC 5.8.0238	Depositor
R, R_{free}	0.210 , 0.238<br>0.217 , 0.243	Depositor<br>DCC
R_{free} test set	4463 reflections (5.00%)	wwPDB-VP
Wilson B-factor (Å^2)	28.2	Xtriage
Anisotropy	0.106	Xtriage
Bulk solvent k_{sol}(e/Å^3), B_{sol}(Å^2)	0.37 , 39.2	EDS
L-test for twinning[2]	$< \|L\| > = 0.52$, $< L^2 > = 0.36$	Xtriage
Estimated twinning fraction	No twinning to report.	Xtriage
F_o,F_c correlation	0.95	EDS
Total number of atoms	9848	wwPDB-VP
Average B, all atoms (Å^2)	36.0	wwPDB-VP

Xtriage's analysis on translational NCS is as follows: *The largest off-origin peak in the Patterson function is 4.74% of the height of the origin peak. No significant pseudotranslation is detected.*

[1]Intensities estimated from amplitudes.

[2]Theoretical values of $< |L| >$, $< L^2 >$ for acentric reflections are 0.5, 0.333 respectively for untwinned datasets, and 0.375, 0.2 for perfectly twinned datasets.

5 Model quality (i)

5.1 Standard geometry (i)

Bond lengths and bond angles in the following residue types are not validated in this section: NA, JUT, NAD

The Z score for a bond length (or angle) is the number of standard deviations the observed value is removed from the expected value. A bond length (or angle) with $|Z| > 5$ is considered an outlier worth inspection. RMSZ is the root-mean-square of all Z scores of the bond lengths (or angles).

Mol	Chain	Bond lengths		Bond angles	
		RMSZ	#|Z| >5	RMSZ	#|Z| >5
1	A	0.85	4/2399 (0.2%)	0.90	2/3242 (0.1%)
1	B	0.83	2/2408 (0.1%)	0.91	1/3253 (0.0%)
1	C	0.92	4/2408 (0.2%)	0.97	4/3253 (0.1%)
1	D	0.88	4/2399 (0.2%)	0.92	1/3242 (0.0%)
All	All	0.87	14/9614 (0.1%)	0.93	8/12990 (0.1%)

Chiral center outliers are detected by calculating the chiral volume of a chiral center and verifying if the center is modelled as a planar moiety or with the opposite hand.A planarity outlier is detected by checking planarity of atoms in a peptide group, atoms in a mainchain group or atoms of a sidechain that are expected to be planar.

Mol	Chain	#Chirality outliers	#Planarity outliers
1	B	0	3

All (14) bond length outliers are listed below:

Mol	Chain	Res	Type	Atoms	Z	Observed(Å)	Ideal(Å)
1	C	22	LEU	C-N	-15.16	0.99	1.34
1	B	283	GLU	CD-OE2	-8.63	1.16	1.25
1	D	194	GLU	CD-OE2	-6.78	1.18	1.25
1	C	23	GLU	C-N	-6.39	1.19	1.34
1	D	23	GLU	CD-OE1	-6.30	1.18	1.25
1	A	205	GLU	CD-OE2	-5.74	1.19	1.25
1	C	283	GLU	CD-OE2	-5.62	1.19	1.25
1	C	122	ASP	C-N	-5.55	1.21	1.34
1	A	103	GLU	CD-OE2	-5.34	1.19	1.25
1	B	290	GLU	CD-OE1	5.29	1.31	1.25
1	A	290	GLU	CD-OE1	5.23	1.31	1.25
1	D	296	GLU	CD-OE1	5.20	1.31	1.25
1	D	103	GLU	CD-OE2	-5.15	1.20	1.25
1	A	194	GLU	CD-OE2	-5.02	1.20	1.25

All (8) bond angle outliers are listed below:

Mol	Chain	Res	Type	Atoms	Z	Observed(^{o})	Ideal(^{o})
1	C	22	LEU	O-C-N	9.28	137.55	122.70
1	D	312	ILE	CA-C-O	-6.83	105.75	120.10
1	A	150	ARG	NE-CZ-NH1	-6.17	117.22	120.30
1	B	183	ARG	NE-CZ-NH2	6.06	123.33	120.30
1	C	23	GLU	O-C-N	-5.28	114.26	122.70
1	A	312	ILE	CA-C-O	-5.24	109.09	120.10
1	C	22	LEU	CA-C-N	-5.24	105.68	117.20
1	C	23	GLU	C-N-CA	5.17	134.62	121.70

There are no chirality outliers.

All (3) planarity outliers are listed below:

Mol	Chain	Res	Type	Group
1	B	118	SER	Peptide
1	B	78	GLY	Peptide
1	B	90	LEU	Peptide

5.2 Too-close contacts (i)

In the following table, the Non-H and H(model) columns list the number of non-hydrogen atoms and hydrogen atoms in the chain respectively. The H(added) column lists the number of hydrogen atoms added and optimized by MolProbity. The Clashes column lists the number of clashes within the asymmetric unit, whereas Symm-Clashes lists symmetry related clashes.

Mol	Chain	Non-H	H(model)	H(added)	Clashes	Symm-Clashes
1	A	2366	0	2438	58	0
1	B	2375	0	2451	40	1
1	C	2375	0	2449	42	0
1	D	2366	0	2438	23	1
2	A	14	0	0	0	0
2	C	14	0	0	7	0
2	D	28	0	0	1	0
3	A	1	0	0	0	0
4	C	44	0	26	1	1
5	A	69	0	0	19	1
5	B	56	0	0	7	0
5	C	59	0	0	2	0
5	D	81	0	0	5	0
All	All	9848	0	9802	162	2

The all-atom clashscore is defined as the number of clashes found per 1000 atoms (including hydrogen atoms). The all-atom clashscore for this structure is 8.

All (162) close contacts within the same asymmetric unit are listed below, sorted by their clash magnitude.

Atom-1	Atom-2	Interatomic distance (Å)	Clash overlap (Å)
1:A:158:LYS:CD	1:A:209:ILE:HD11	1.55	1.34
1:A:306:GLN:O	5:A:502:HOH:O	1.53	1.21
1:A:158:LYS:HE2	1:A:205:GLU:HB3	1.30	1.12
1:C:238:LYS:HE2	1:C:251:PHE:CE1	1.87	1.10
1:D:82:LYS:O	5:D:501:HOH:O	1.71	1.08
1:B:90:LEU:H	1:B:90:LEU:HD12	1.17	1.07
1:A:238:LYS:CD	1:A:251:PHE:HE1	1.66	1.06
1:A:158:LYS:HD2	1:A:209:ILE:HD11	1.12	1.04
1:C:250:LEU:HD23	2:C:402:JUT:CAN	1.90	1.01
1:C:238:LYS:CD	1:C:251:PHE:HE1	1.72	1.01
1:A:158:LYS:HD3	1:A:209:ILE:HD11	1.37	1.00
1:C:81:ARG:NH1	1:C:87:ARG:CG	2.27	0.98
1:B:3:LYS:HE2	1:B:29:ILE:HD11	1.47	0.96
1:A:158:LYS:CD	1:A:209:ILE:CD1	2.44	0.95
1:A:238:LYS:HD3	1:A:251:PHE:HE1	1.28	0.94
1:C:81:ARG:NH1	1:C:87:ARG:HG3	1.84	0.93
1:B:313:LYS:O	5:B:401:HOH:O	1.85	0.93
1:C:238:LYS:CE	1:C:251:PHE:CE1	2.54	0.91
1:C:238:LYS:CE	1:C:251:PHE:HE1	1.84	0.90
1:A:238:LYS:CE	1:A:251:PHE:HE1	1.84	0.90
1:A:238:LYS:HD3	1:A:251:PHE:CE1	2.08	0.89
1:A:158:LYS:HD2	1:A:209:ILE:CD1	2.01	0.88
1:B:88:GLU:O	1:B:91:ILE:CG2	2.22	0.87
1:A:158:LYS:HD3	1:A:209:ILE:CD1	2.04	0.87
1:D:79:VAL:H	1:D:94:ASN:HD21	1.21	0.87
1:C:81:ARG:NH1	1:C:87:ARG:HG2	1.89	0.86
1:B:90:LEU:CD1	1:B:90:LEU:H	1.89	0.85
1:C:81:ARG:HH11	1:C:87:ARG:HG2	1.37	0.84
1:A:158:LYS:CE	1:A:205:GLU:HB3	2.06	0.84
1:A:238:LYS:CE	1:A:251:PHE:CE1	2.60	0.84
1:A:238:LYS:HE3	1:A:251:PHE:CE1	2.12	0.84
1:A:123:ILE:HD11	1:A:304:ASN:HB3	1.59	0.82
1:A:238:LYS:CD	1:A:251:PHE:CE1	2.59	0.82
1:A:121:LEU:HD12	5:A:556:HOH:O	1.78	0.81
1:C:3:LYS:HE2	1:C:68:LYS:O	1.80	0.81
1:C:238:LYS:HD3	1:C:251:PHE:HE1	1.44	0.81
1:C:81:ARG:HH12	1:C:87:ARG:HG3	1.46	0.80

Continued on next page...

Continued from previous page...

Atom-1	Atom-2	Interatomic distance (Å)	Clash overlap (Å)
1:A:312:ILE:C	5:A:510:HOH:O	2.20	0.80
1:A:65:GLU:OE2	5:A:503:HOH:O	2.00	0.79
1:D:121:LEU:HD22	5:D:571:HOH:O	1.84	0.78
1:B:90:LEU:CD2	1:B:94:ASN:ND2	2.48	0.77
1:B:183:ARG:HH12	1:B:290:GLU:CD	1.88	0.75
1:C:238:LYS:CD	1:C:251:PHE:CE1	2.64	0.75
1:A:177:LEU:HD21	1:A:300:SER:OG	1.88	0.74
1:A:79:VAL:HG23	5:A:506:HOH:O	1.87	0.74
1:A:307:LYS:C	5:A:502:HOH:O	2.26	0.73
1:C:250:LEU:CD2	2:C:402:JUT:CAG	2.67	0.73
1:B:90:LEU:N	1:B:90:LEU:HD12	2.00	0.73
1:B:183:ARG:NH1	1:B:290:GLU:OE2	2.23	0.72
1:B:305:THR:OG1	5:B:402:HOH:O	2.04	0.72
1:B:121:LEU:HD12	5:B:451:HOH:O	1.91	0.71
1:B:88:GLU:O	1:B:91:ILE:HG21	1.90	0.69
1:C:250:LEU:HD23	2:C:402:JUT:CAG	2.22	0.69
1:C:250:LEU:CD2	2:C:402:JUT:CAN	2.68	0.68
1:A:306:GLN:NE2	5:A:505:HOH:O	2.17	0.68
4:C:401:NAD:O2B	5:C:501:HOH:O	2.12	0.67
1:C:121:LEU:HD22	1:C:143:ALA:HB2	1.77	0.66
1:A:90:LEU:N	5:A:509:HOH:O	2.28	0.66
1:A:87:ARG:NH1	5:A:504:HOH:O	2.15	0.66
1:A:5:ALA:HB3	1:A:73:ILE:HD13	1.77	0.66
1:C:238:LYS:HD3	1:C:251:PHE:CE1	2.30	0.65
1:A:307:LYS:O	5:A:502:HOH:O	2.15	0.65
1:D:83:GLU:C	5:D:501:HOH:O	2.35	0.65
1:C:183:ARG:NH1	1:C:290:GLU:OE2	2.30	0.65
1:C:81:ARG:HH12	1:C:87:ARG:CG	2.02	0.64
1:A:134:LEU:HB2	1:A:139:ILE:HD11	1.80	0.63
1:A:91:ILE:N	5:A:509:HOH:O	2.32	0.63
1:D:83:GLU:O	5:D:501:HOH:O	2.16	0.63
1:A:121:LEU:CD2	1:A:143:ALA:HB2	2.29	0.62
1:A:106:LYS:HE2	1:A:132:SER:O	1.99	0.62
1:C:158:LYS:HG3	1:C:209:ILE:HD11	1.81	0.62
1:B:121:LEU:CD2	1:B:143:ALA:HB2	2.30	0.62
1:B:5:ALA:HB3	1:B:73:ILE:HD13	1.81	0.62
1:C:250:LEU:HD23	2:C:402:JUT:CAL	2.29	0.61
1:D:121:LEU:C	1:D:121:LEU:HD13	2.19	0.61
1:B:90:LEU:HD22	1:B:94:ASN:ND2	2.13	0.61
1:C:183:ARG:HH12	1:C:290:GLU:CD	2.04	0.61
1:D:193:SER:O	1:D:197:LYS:HG3	2.01	0.61

Continued on next page...

Continued from previous page...

Atom-1	Atom-2	Interatomic distance (Å)	Clash overlap (Å)
1:C:120:PRO:HG2	1:C:123:ILE:HD12	1.83	0.60
1:B:301:VAL:HG13	5:B:402:HOH:O	2.02	0.59
1:A:120:PRO:HG2	1:A:123:ILE:HD12	1.85	0.59
1:C:238:LYS:HG2	1:C:251:PHE:CZ	2.37	0.59
1:B:123:ILE:HD11	1:B:304:ASN:HB3	1.85	0.58
1:D:123:ILE:HD11	1:D:304:ASN:HB3	1.86	0.57
1:B:90:LEU:HB2	1:B:91:ILE:HA	1.87	0.57
1:B:90:LEU:HD23	1:B:94:ASN:ND2	2.18	0.57
1:C:5:ALA:HB3	1:C:73:ILE:HD13	1.85	0.56
1:A:121:LEU:HD22	1:A:142:MET:O	2.05	0.56
1:A:121:LEU:HD22	1:A:143:ALA:HB2	1.87	0.56
1:C:238:LYS:HG2	1:C:251:PHE:CE1	2.40	0.56
1:B:22:LEU:HD21	1:B:52:LEU:HD13	1.88	0.56
1:D:5:ALA:HB3	1:D:73:ILE:HD13	1.87	0.56
1:B:121:LEU:HD22	1:B:142:MET:O	2.06	0.55
1:D:121:LEU:HD13	1:D:121:LEU:O	2.07	0.55
1:B:87:ARG:O	1:B:90:LEU:CD1	2.54	0.55
1:B:121:LEU:HD22	1:B:143:ALA:HB2	1.89	0.54
1:A:87:ARG:NH1	5:A:512:HOH:O	2.41	0.54
1:B:145:ILE:HD11	1:B:251:PHE:CD1	2.43	0.53
1:C:145:ILE:HD11	1:C:251:PHE:CD1	2.43	0.53
1:C:312:ILE:O	1:C:312:ILE:HG22	2.08	0.53
1:B:95:GLY:HA2	1:B:124:MET:CE	2.39	0.53
1:C:250:LEU:HG	2:C:402:JUT:CAH	2.39	0.53
1:A:120:PRO:HD3	5:A:509:HOH:O	2.09	0.53
1:A:303:SER:O	1:A:307:LYS:HG2	2.09	0.52
1:A:120:PRO:CG	1:A:123:ILE:HD12	2.39	0.52
1:A:94:ASN:OD1	5:A:506:HOH:O	2.19	0.52
1:B:48:PHE:CZ	1:B:52:LEU:HD12	2.45	0.52
1:D:252:THR:HA	1:D:270:SER:O	2.10	0.51
1:B:116:CYS:HB2	1:B:125:VAL:HG22	1.91	0.51
1:A:145:ILE:HD11	1:A:251:PHE:CD1	2.45	0.51
1:D:145:ILE:HD11	1:D:251:PHE:CD1	2.46	0.51
1:C:123:ILE:HD11	1:C:304:ASN:HB3	1.93	0.50
1:B:302:GLN:HG3	5:B:434:HOH:O	2.12	0.49
1:A:110:SER:HA	1:A:134:LEU:HD21	1.93	0.49
1:B:252:THR:HA	1:B:270:SER:O	2.13	0.49
1:C:121:LEU:O	1:C:121:LEU:HG	2.11	0.48
1:A:252:THR:HA	1:A:270:SER:O	2.13	0.48
1:D:83:GLU:CA	5:D:501:HOH:O	2.61	0.48
1:A:91:ILE:HG21	5:A:560:HOH:O	2.12	0.48

Continued on next page...

Continued from previous page...

Atom-1	Atom-2	Interatomic distance (Å)	Clash overlap (Å)
1:A:121:LEU:HD23	1:A:143:ALA:HB2	1.96	0.48
1:C:238:LYS:HE2	1:C:251:PHE:CZ	2.41	0.48
1:D:282:VAL:HG11	2:D:402:JUT:NAC	2.29	0.48
1:B:119:ASN:ND2	5:B:405:HOH:O	2.46	0.48
1:A:286:LEU:HD12	1:A:291:GLN:NE2	2.29	0.47
1:B:87:ARG:O	1:B:90:LEU:HD11	2.14	0.47
1:C:238:LYS:CG	1:C:251:PHE:CE1	2.97	0.47
1:C:252:THR:HA	1:C:270:SER:O	2.14	0.47
1:B:88:GLU:O	1:B:91:ILE:HG22	2.14	0.46
1:C:81:ARG:HH11	1:C:87:ARG:CG	2.02	0.46
1:A:116:CYS:HB2	1:A:125:VAL:HG22	1.97	0.46
1:B:121:LEU:HD23	1:B:143:ALA:HB2	1.95	0.46
1:A:309:PHE:CD1	5:A:536:HOH:O	2.56	0.46
1:C:46:LYS:NZ	5:C:505:HOH:O	2.43	0.46
1:B:95:GLY:HA2	1:B:124:MET:HE1	1.98	0.46
1:A:307:LYS:HB2	5:A:560:HOH:O	2.16	0.45
1:A:123:ILE:CD1	1:A:304:ASN:HB3	2.40	0.45
1:C:130:LYS:HD3	1:C:309:PHE:CE1	2.52	0.45
1:A:87:ARG:O	1:A:90:LEU:HD12	2.17	0.44
1:B:48:PHE:CZ	1:B:52:LEU:CD1	3.01	0.44
1:D:82:LYS:HG2	1:D:85:MET:CE	2.48	0.44
1:C:3:LYS:CE	1:C:68:LYS:O	2.59	0.44
1:D:121:LEU:C	1:D:121:LEU:CD1	2.85	0.44
1:D:303:SER:O	1:D:307:LYS:HG2	2.17	0.43
1:C:194:GLU:OE1	1:C:194:GLU:HA	2.19	0.43
1:D:23:GLU:HB3	1:D:25:LEU:HG	2.00	0.43
1:B:48:PHE:CE1	1:B:52:LEU:HD12	2.53	0.43
1:A:130:LYS:HD3	1:A:309:PHE:CE1	2.54	0.42
1:A:308:ALA:HB3	5:A:536:HOH:O	2.19	0.42
1:B:130:LYS:HD3	1:B:309:PHE:CE1	2.55	0.42
1:B:91:ILE:HG13	5:B:419:HOH:O	2.18	0.42
1:C:308:ALA:O	1:C:312:ILE:HG13	2.19	0.42
1:C:250:LEU:HD21	2:C:402:JUT:CAG	2.47	0.42
1:D:257:LEU:HG	1:D:268:VAL:HG22	2.02	0.41
1:D:266:LEU:HD23	1:D:268:VAL:CG1	2.50	0.41
1:A:287:THR:CG2	1:A:289:GLU:HG2	2.51	0.41
1:D:82:LYS:HA	1:D:82:LYS:HD3	1.92	0.41
1:A:37:ILE:HD11	1:D:225:LYS:HD2	2.01	0.41
1:B:3:LYS:HE2	1:B:29:ILE:CD1	2.34	0.41
1:A:256:TYR:CE2	1:A:258:ASN:HB2	2.56	0.41
1:A:90:LEU:CA	5:A:509:HOH:O	2.68	0.41

Continued on next page...

Continued from previous page...

Atom-1	Atom-2	Interatomic distance (Å)	Clash overlap (Å)
1:B:285:PRO:HG2	1:D:194:GLU:OE1	2.20	0.41
1:A:287:THR:HG22	1:A:289:GLU:HG2	2.02	0.40

All (2) symmetry-related close contacts are listed below. The label for Atom-2 includes the symmetry operator and encoded unit-cell translations to be applied.

Atom-1	Atom-2	Interatomic distance (Å)	Clash overlap (Å)
1:B:259:GLY:O	1:D:84:GLY:O[3_554]	1.73	0.47
4:C:401:NAD:N7A	5:A:503:HOH:O[2_565]	2.04	0.16

5.3 Torsion angles (i)

5.3.1 Protein backbone (i)

In the following table, the Percentiles column shows the percent Ramachandran outliers of the chain as a percentile score with respect to all X-ray entries followed by that with respect to entries of similar resolution.

The Analysed column shows the number of residues for which the backbone conformation was analysed, and the total number of residues.

Mol	Chain	Analysed	Favoured	Allowed	Outliers	Percentiles	
1	A	309/324 (95%)	303 (98%)	6 (2%)	0	100	100
1	B	310/324 (96%)	302 (97%)	8 (3%)	0	100	100
1	C	310/324 (96%)	305 (98%)	5 (2%)	0	100	100
1	D	309/324 (95%)	304 (98%)	5 (2%)	0	100	100
All	All	1238/1296 (96%)	1214 (98%)	24 (2%)	0	100	100

There are no Ramachandran outliers to report.

5.3.2 Protein sidechains (i)

In the following table, the Percentiles column shows the percent sidechain outliers of the chain as a percentile score with respect to all X-ray entries followed by that with respect to entries of similar resolution.

The Analysed column shows the number of residues for which the sidechain conformation was analysed, and the total number of residues.

Mol	Chain	Analysed	Rotameric	Outliers	Percentiles	
1	A	263/273 (96%)	263 (100%)	0	100	100
1	B	264/273 (97%)	261 (99%)	3 (1%)	73	78
1	C	264/273 (97%)	263 (100%)	1 (0%)	91	93
1	D	263/273 (96%)	262 (100%)	1 (0%)	91	93
All	All	1054/1092 (96%)	1049 (100%)	5 (0%)	88	92

All (5) residues with a non-rotameric sidechain are listed below:

Mol	Chain	Res	Type
1	B	90	LEU
1	B	91	ILE
1	B	158	LYS
1	C	24	ASN
1	D	158	LYS

Some sidechains can be flipped to improve hydrogen bonding and reduce clashes. All (4) such sidechains are listed below:

Mol	Chain	Res	Type
1	A	291	GLN
1	B	11	GLN
1	C	11	GLN
1	D	94	ASN

5.3.3 RNA (i)

There are no RNA molecules in this entry.

5.4 Non-standard residues in protein, DNA, RNA chains (i)

There are no non-standard protein/DNA/RNA residues in this entry.

5.5 Carbohydrates (i)

There are no carbohydrates in this entry.

5.6 Ligand geometry (i)

Of 6 ligands modelled in this entry, 1 is monoatomic - leaving 5 for Mogul analysis.

In the following table, the Counts columns list the number of bonds (or angles) for which Mogul statistics could be retrieved, the number of bonds (or angles) that are observed in the model and the number of bonds (or angles) that are defined in the Chemical Component Dictionary. The Link column lists molecule types, if any, to which the group is linked. The Z score for a bond length (or angle) is the number of standard deviations the observed value is removed from the expected value. A bond length (or angle) with $|Z| > 2$ is considered an outlier worth inspection. RMSZ is the root-mean-square of all Z scores of the bond lengths (or angles).

Mol	Type	Chain	Res	Link	Bond lengths			Bond angles		
					Counts	RMSZ	#|Z| > 2	Counts	RMSZ	#|Z| > 2
2	JUT	C	402	-	12,15,15	3.56	6 (50%)	16,21,21	4.04	11 (68%)
4	NAD	C	401	-	42,48,48	1.14	2 (4%)	50,73,73	0.99	3 (6%)
2	JUT	A	401	-	12,15,15	3.86	4 (33%)	16,21,21	3.00	8 (50%)
2	JUT	D	401	-	12,15,15	2.95	4 (33%)	16,21,21	4.61	11 (68%)
2	JUT	D	402	-	12,15,15	5.43	6 (50%)	16,21,21	7.21	13 (81%)

In the following table, the Chirals column lists the number of chiral outliers, the number of chiral centers analysed, the number of these observed in the model and the number defined in the Chemical Component Dictionary. Similar counts are reported in the Torsion and Rings columns. '-' means no outliers of that kind were identified.

Mol	Type	Chain	Res	Link	Chirals	Torsions	Rings
2	JUT	C	402	-	-	0/4/4/4	0/2/2/2
4	NAD	C	401	-	-	5/26/62/62	0/5/5/5
2	JUT	A	401	-	-	0/4/4/4	0/2/2/2
2	JUT	D	401	-	-	0/4/4/4	0/2/2/2
2	JUT	D	402	-	-	0/4/4/4	0/2/2/2

All (22) bond length outliers are listed below:

Mol	Chain	Res	Type	Atoms	Z	Observed(Å)	Ideal(Å)
2	D	402	JUT	CAE-SAF	15.13	1.94	1.70
2	C	402	JUT	CAG-CAD	-10.37	1.32	1.48
2	A	401	JUT	CAG-CAD	-9.74	1.33	1.48
2	D	401	JUT	CAG-CAD	-8.10	1.36	1.48
2	A	401	JUT	FAM-CAL	-6.73	1.18	1.35
2	D	402	JUT	CAN-CAL	6.61	1.49	1.37
2	D	402	JUT	CAG-CAD	-5.80	1.39	1.48
2	A	401	JUT	CAE-SAF	4.80	1.78	1.70
2	D	402	JUT	CAN-CAG	4.52	1.47	1.39
4	C	401	NAD	O4D-C1D	4.14	1.46	1.41
2	D	401	JUT	FAM-CAL	3.79	1.45	1.35
2	D	402	JUT	FAM-CAL	3.55	1.44	1.35

Continued on next page...

Continued from previous page...

Mol	Chain	Res	Type	Atoms	Z	Observed(Å)	Ideal(Å)
2	C	402	JUT	CAN-CAL	3.47	1.43	1.37
4	C	401	NAD	C2N-N1N	3.21	1.38	1.35
2	C	402	JUT	FAM-CAL	-3.07	1.27	1.35
2	A	401	JUT	CAN-CAL	2.57	1.42	1.37
2	D	401	JUT	CAH-CAI	-2.32	1.34	1.38
2	D	402	JUT	CAI-CAJ	-2.32	1.33	1.37
2	C	402	JUT	FAK-CAJ	-2.28	1.29	1.35
2	C	402	JUT	CAE-SAF	2.26	1.74	1.70
2	C	402	JUT	CAI-CAJ	-2.14	1.33	1.37
2	D	401	JUT	CAB-NAA	-2.01	1.29	1.35

All (46) bond angle outliers are listed below:

Mol	Chain	Res	Type	Atoms	Z	Observed(^{o})	Ideal(^{o})
2	D	402	JUT	CAN-CAG-CAD	16.54	144.54	120.59
2	D	402	JUT	CAH-CAG-CAD	-13.30	100.27	121.28
2	C	402	JUT	CAH-CAG-CAD	-8.71	107.53	121.28
2	D	401	JUT	CAH-CAG-CAD	-8.65	107.63	121.28
2	D	402	JUT	FAM-CAL-CAN	8.57	135.66	118.61
2	D	402	JUT	FAM-CAL-CAJ	-7.87	99.30	118.43
2	D	401	JUT	CAN-CAG-CAD	7.61	131.61	120.59
2	D	402	JUT	CAG-CAN-CAL	-7.50	113.20	119.59
2	D	401	JUT	NAA-CAB-NAC	7.24	132.56	123.19
2	C	402	JUT	CAN-CAG-CAD	7.22	131.04	120.59
2	D	401	JUT	CAE-CAD-CAG	-7.04	119.66	129.44
2	D	402	JUT	CAG-CAD-NAC	-6.25	110.29	120.78
2	D	402	JUT	CAD-CAE-SAF	-5.98	104.45	111.79
2	D	402	JUT	CAE-CAD-CAG	5.95	137.71	129.44
2	D	402	JUT	CAI-CAH-CAG	5.79	129.46	121.13
2	A	401	JUT	CAH-CAG-CAD	-5.76	112.19	121.28
2	D	401	JUT	CAG-CAN-CAL	-5.46	114.94	119.59
2	C	402	JUT	CAG-CAN-CAL	-5.37	115.01	119.59
2	C	402	JUT	CAD-CAE-SAF	-5.28	105.31	111.79
2	A	401	JUT	CAD-CAE-SAF	-5.26	105.33	111.79
2	A	401	JUT	CAN-CAG-CAD	4.92	127.71	120.59
2	D	401	JUT	FAM-CAL-CAN	4.66	127.88	118.61
2	D	402	JUT	NAA-CAB-NAC	4.19	128.61	123.19
2	C	402	JUT	CAE-CAD-CAG	-4.12	123.72	129.44
2	D	401	JUT	CAD-CAE-SAF	-4.02	106.86	111.79
2	A	401	JUT	CAE-CAD-CAG	-3.99	123.89	129.44
2	C	402	JUT	FAK-CAJ-CAL	3.92	127.96	118.43
2	C	402	JUT	NAA-CAB-NAC	3.59	127.84	123.19

Continued on next page...

Continued from previous page...

Mol	Chain	Res	Type	Atoms	Z	Observed(°)	Ideal(°)
2	D	402	JUT	CAN-CAL-CAJ	3.52	125.04	121.03
2	A	401	JUT	FAK-CAJ-CAL	3.34	126.54	118.43
2	C	402	JUT	FAM-CAL-CAN	3.31	125.20	118.61
2	D	401	JUT	FAM-CAL-CAJ	-3.21	110.64	118.43
2	D	401	JUT	FAK-CAJ-CAL	3.10	125.96	118.43
2	A	401	JUT	CAG-CAN-CAL	-2.95	117.08	119.59
4	C	401	NAD	C6N-N1N-C2N	-2.84	119.38	121.97
4	C	401	NAD	O2A-PA-O1A	2.83	126.21	112.24
2	A	401	JUT	NAA-CAB-NAC	2.80	126.82	123.19
2	D	402	JUT	FAK-CAJ-CAL	2.79	125.20	118.43
2	C	402	JUT	FAK-CAJ-CAI	-2.70	112.57	118.59
2	D	401	JUT	FAK-CAJ-CAI	-2.55	112.92	118.59
2	D	401	JUT	CAG-CAD-NAC	2.45	124.89	120.78
2	D	402	JUT	CAH-CAG-CAN	-2.29	114.92	118.16
4	C	401	NAD	C3N-C2N-N1N	2.16	122.54	120.43
2	C	402	JUT	CAH-CAG-CAN	2.15	121.20	118.16
2	C	402	JUT	FAM-CAL-CAJ	-2.04	113.48	118.43
2	A	401	JUT	FAM-CAL-CAN	2.01	122.61	118.61

There are no chirality outliers.

All (5) torsion outliers are listed below:

Mol	Chain	Res	Type	Atoms
4	C	401	NAD	O4D-C1D-N1N-C2N
4	C	401	NAD	O4D-C1D-N1N-C6N
4	C	401	NAD	C2D-C1D-N1N-C2N
4	C	401	NAD	C2D-C1D-N1N-C6N
4	C	401	NAD	O4B-C4B-C5B-O5B

There are no ring outliers.

3 monomers are involved in 10 short contacts:

Mol	Chain	Res	Type	Clashes	Symm-Clashes
2	C	402	JUT	7	0
4	C	401	NAD	1	1
2	D	402	JUT	1	0

The following is a two-dimensional graphical depiction of Mogul quality analysis of bond lengths, bond angles, torsion angles, and ring geometry for all instances of the Ligand of Interest. In addition, ligands with molecular weight > 250 and outliers as shown on the validation Tables will also be included. For torsion angles, if less then 5% of the Mogul distribution of torsion angles is within 10 degrees of the torsion angle in question, then that torsion angle is considered an outlier.

Any bond that is central to one or more torsion angles identified as an outlier by Mogul will be highlighted in the graph. For rings, the root-mean-square deviation (RMSD) between the ring in question and similar rings identified by Mogul is calculated over all ring torsion angles. If the average RMSD is greater than 60 degrees and the minimal RMSD between the ring in question and any Mogul-identified rings is also greater than 60 degrees, then that ring is considered an outlier. The outliers are highlighted in purple. The color gray indicates Mogul did not find sufficient equivalents in the CSD to analyse the geometry.

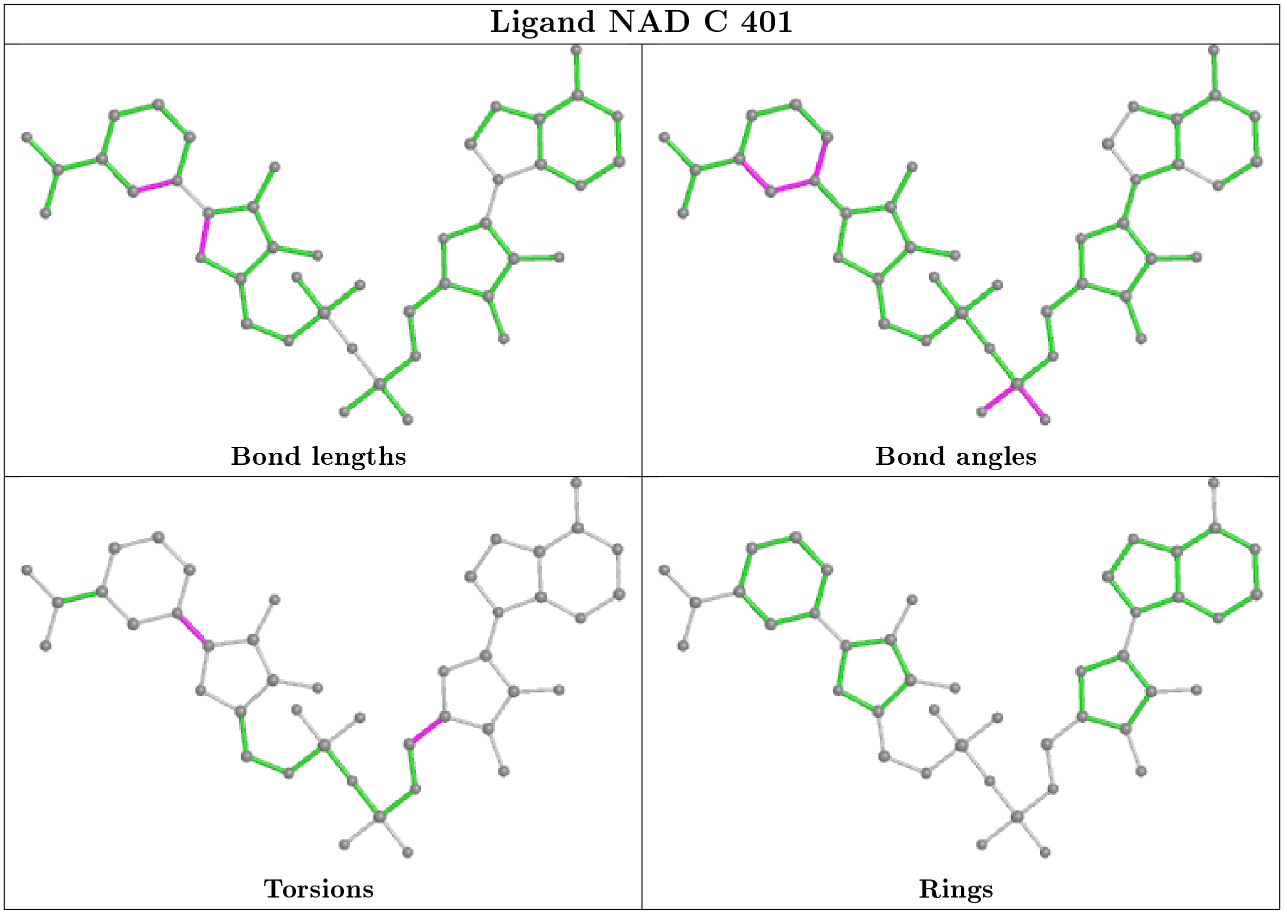

5.7 Other polymers (i)

There are no such residues in this entry.

5.8 Polymer linkage issues (i)

The following chains have linkage breaks:

Mol	Chain	Number of breaks
1	C	2

All chain breaks are listed below:

Model	Chain	Residue-1	Atom-1	Residue-2	Atom-2	Distance (Å)
1	C	23:GLU	C	24:ASN	N	1.19
1	C	22:LEU	C	23:GLU	N	0.99

6 Fit of model and data

6.1 Protein, DNA and RNA chains

In the following table, the column labelled '#RSRZ> 2' contains the number (and percentage) of RSRZ outliers, followed by percent RSRZ outliers for the chain as percentile scores relative to all X-ray entries and entries of similar resolution. The OWAB column contains the minimum, median, 95th percentile and maximum values of the occupancy-weighted average B-factor per residue. The column labelled 'Q< 0.9' lists the number of (and percentage) of residues with an average occupancy less than 0.9.

Mol	Chain	Analysed	<RSRZ>	#RSRZ>2			OWAB(Å^2)	Q<0.9
1	A	311/324 (95%)	0.43	30 (9%)	8	7	21, 35, 66, 97	0
1	B	312/324 (96%)	0.40	21 (6%)	17	17	21, 35, 59, 142	0
1	C	312/324 (96%)	0.04	6 (1%)	66	65	19, 30, 56, 76	0
1	D	311/324 (95%)	-0.09	3 (0%)	82	81	19, 29, 44, 83	0
All	All	1246/1296 (96%)	0.20	60 (4%)	30	29	19, 32, 58, 142	0

All (60) RSRZ outliers are listed below:

Mol	Chain	Res	Type	RSRZ
1	B	85	MET	10.5
1	B	84	GLY	7.9
1	B	90	LEU	7.5
1	B	88	GLU	7.4
1	B	82	LYS	7.2
1	D	83	GLU	7.2
1	B	86	THR	6.9
1	A	87	ARG	5.9
1	B	89	ASP	5.9
1	B	119	ASN	5.6
1	A	84	GLY	5.6
1	B	87	ARG	5.6
1	A	89	ASP	5.4
1	A	90	LEU	5.4
1	A	88	GLU	5.1
1	D	84	GLY	5.0
1	A	86	THR	4.6
1	A	85	MET	4.5
1	B	80	GLN	4.4
1	A	81	ARG	4.2
1	A	82	LYS	4.1

Continued on next page...

Continued from previous page...

Mol	Chain	Res	Type	RSRZ
1	A	80	GLN	3.9
1	A	177	LEU	3.8
1	B	81	ARG	3.8
1	A	83	GLU	3.7
1	B	83	GLU	3.6
1	B	93	VAL	3.5
1	A	158	LYS	3.5
1	A	93	VAL	3.2
1	A	175	GLY	3.2
1	A	22	LEU	3.2
1	D	82	LYS	3.2
1	A	96	LYS	2.9
1	A	309	PHE	2.8
1	A	133	ASN	2.7
1	B	176	ASP	2.7
1	A	306	GLN	2.7
1	B	79	VAL	2.7
1	C	238	LYS	2.6
1	A	91	ILE	2.6
1	C	175	GLY	2.6
1	B	52	LEU	2.6
1	C	289	GLU	2.5
1	A	19	LEU	2.4
1	B	91	ILE	2.4
1	C	87	ARG	2.3
1	A	176	ASP	2.3
1	A	174	HIS	2.2
1	B	289	GLU	2.2
1	C	83	GLU	2.2
1	A	20	CYS	2.2
1	A	16	VAL	2.2
1	A	312	ILE	2.1
1	A	289	GLU	2.1
1	A	238	LYS	2.1
1	C	313	LYS	2.1
1	A	302	GLN	2.1
1	B	54	VAL	2.0
1	B	92	GLY	2.0
1	B	22	LEU	2.0

6.2 Non-standard residues in protein, DNA, RNA chains (i)

There are no non-standard protein/DNA/RNA residues in this entry.

6.3 Carbohydrates (i)

There are no carbohydrates in this entry.

6.4 Ligands (i)

In the following table, the Atoms column lists the number of modelled atoms in the group and the number defined in the chemical component dictionary. The B-factors column lists the minimum, median, 95th percentile and maximum values of B factors of atoms in the group. The column labelled 'Q< 0.9' lists the number of atoms with occupancy less than 0.9.

Mol	Type	Chain	Res	Atoms	RSCC	RSR	B-factors(Å^2)	Q<0.9
2	JUT	A	401	14/14	0.85	0.23	53,55,61,71	0
2	JUT	C	402	14/14	0.85	0.27	53,55,63,70	0
2	JUT	D	401	14/14	0.90	0.14	39,42,50,57	0
2	JUT	D	402	14/14	0.91	0.23	38,39,49,51	0
3	NA	A	402	1/1	0.92	0.14	46,46,46,46	0
4	NAD	C	401	44/44	0.94	0.13	30,38,49,55	0

The following is a graphical depiction of the model fit to experimental electron density of all instances of the Ligand of Interest. In addition, ligands with molecular weight > 250 and outliers as shown on the geometry validation Tables will also be included. Each fit is shown from different orientation to approximate a three-dimensional view.

Electron density around NAD C 401:

$2mF_o$-DF_c (at 0.7 rmsd) in gray
mF_o-DF_c (at 3 rmsd) in purple (negative)
and green (positive)

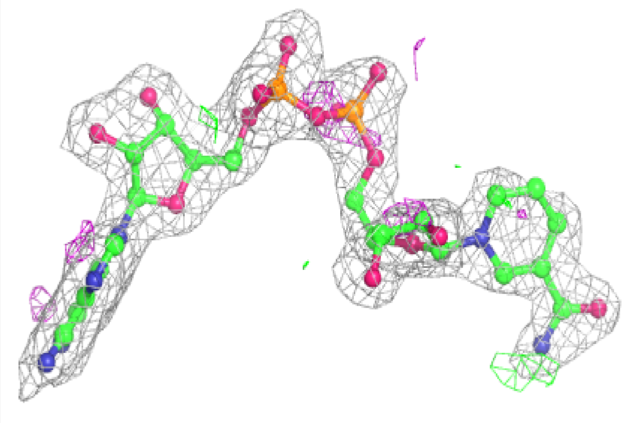

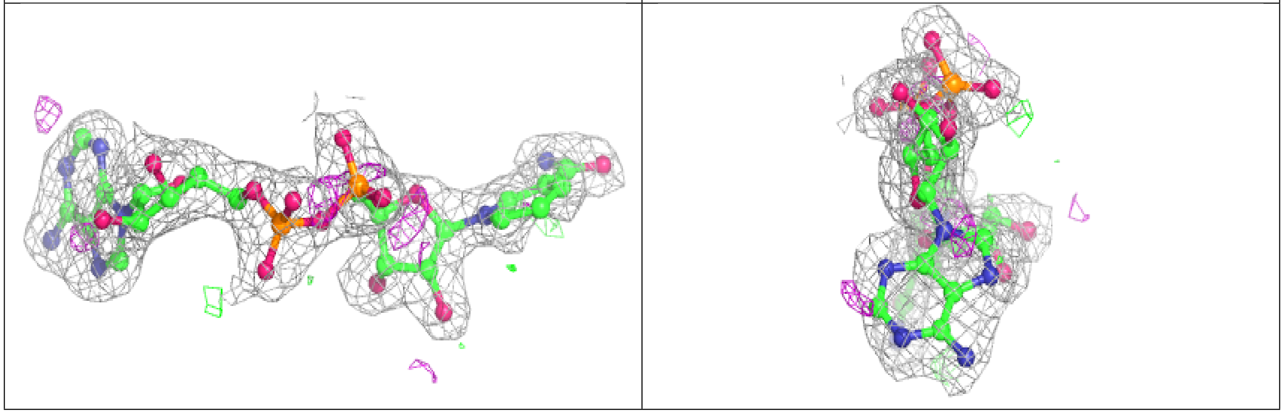

6.5 Other polymers ⓘ

There are no such residues in this entry.
