# Supplementary material for: A fragment-based approach identifies an allosteric pocket that impacts malate dehydrogenase activity
Source: Commun Biol. 2021 Aug 10;4:949. doi: 10.1038/s42003-021-02442-1 (PMC8355244; doi:10.1038/s42003-021-02442-1)
Supplement: Supplementary file 9 — Supplementary Data 6 [file 42003_2021_2442_MOESM9_ESM.pdf]

Full wwPDB X-ray Structure Validation Report ⓘ

CONFIDENTIAL VALIDATION REPORT

Mar 6, 2020 – 01:54 PM GMT

PDB ID	:	6Y91
Title	:	Crystal structure of malate dehydrogenase from Plasmodium Falciparum in complex with 4-(3,4-difluorophenyl)thiazol-2-amine
Deposited on	:	2020-03-06
Resolution	:	2.50 Å(reported)

This is a Full wwPDB X-ray Structure Validation Report.

This report is produced by the wwPDB biocuration pipeline after annotation of the structure.

We welcome your comments at *validation@mail.wwpdb.org*
A user guide is available at
https://www.wwpdb.org/validation/2017/XrayValidationReportHelp
with specific help available everywhere you see the ⓘ symbol.

The following versions of software and data (see references ⓘ) were used in the production of this report:

MolProbity	:	4.02b-467
Mogul	:	1.8.0 (224370), CSD as540be (2019)
Xtriage (Phenix)	:	1.13
EDS	:	2.9
buster-report	:	1.1.7 (2018)
Percentile statistics	:	20171227.v01 (using entries in the PDB archive December 27th 2017)
Refmac	:	5.8.0158
CCP4	:	7.0.044 (Gargrove)
Ideal geometry (proteins)	:	Engh & Huber (2001)
Ideal geometry (DNA, RNA)	:	Parkinson et al. (1996)
Validation Pipeline (wwPDB-VP)	:	2.9

1 Overall quality at a glance (i)

The following experimental techniques were used to determine the structure:
X-RAY DIFFRACTION

The reported resolution of this entry is 2.50 Å.

Percentile scores (ranging between 0-100) for global validation metrics of the entry are shown in the following graphic. The table shows the number of entries on which the scores are based.

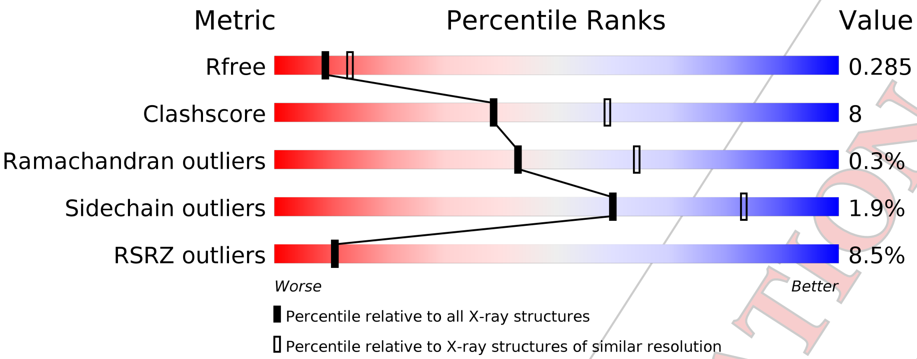

Metric	Whole archive (#Entries)	Similar resolution (#Entries, resolution range(Å))
R_{free}	111664	4155 (2.50-2.50)
Clashscore	122126	4827 (2.50-2.50)
Ramachandran outliers	120053	4735 (2.50-2.50)
Sidechain outliers	120020	4737 (2.50-2.50)
RSRZ outliers	108989	4058 (2.50-2.50)

The table below summarises the geometric issues observed across the polymeric chains and their fit to the electron density. The red, orange, yellow and green segments on the lower bar indicate the fraction of residues that contain outliers for >=3, 2, 1 and 0 types of geometric quality criteria respectively. A grey segment represents the fraction of residues that are not modelled. The numeric value for each fraction is indicated below the corresponding segment, with a dot representing fractions <=5% The upper red bar (where present) indicates the fraction of residues that have poor fit to the electron density. The numeric value is given above the bar.

Mol	Chain	Length	Quality of chain
1	A	324	11% / 78% 14% • 7%
1	B	324	4% / 79% 13% • 7%
1	C	324	6% / 76% 14% • 7%
1	D	324	10% / 76% 15% • 7%

CONFIDENTIAL VALIDATION REPORT

CONFIDENTIAL VALIDATION REPORT

2 Entry composition (i)

There are 3 unique types of molecules in this entry. The entry contains 9452 atoms, of which 0 are hydrogens and 0 are deuteriums.

In the tables below, the ZeroOcc column contains the number of atoms modelled with zero occupancy, the AltConf column contains the number of residues with at least one atom in alternate conformation and the Trace column contains the number of residues modelled with at most 2 atoms.

- Molecule 1 is a protein called Malate dehydrogenase.

Mol	Chain	Residues	Atoms					ZeroOcc	AltConf	Trace
1	A	302	Total 2287	C 1459	N 384	O 429	S 15	0	0	0
1	B	302	Total 2290	C 1461	N 384	O 431	S 14	0	0	0
1	C	300	Total 2274	C 1450	N 380	O 429	S 15	0	0	0
1	D	301	Total 2282	C 1455	N 383	O 430	S 14	0	0	0

There are 44 discrepancies between the modelled and reference sequences:

Chain	Residue	Modelled	Actual	Comment	Reference
A	314	ALA	-	expression tag	UNP Q6VVP7
A	315	ALA	-	expression tag	UNP Q6VVP7
A	316	ALA	-	expression tag	UNP Q6VVP7
A	317	LEU	-	expression tag	UNP Q6VVP7
A	318	GLU	-	expression tag	UNP Q6VVP7
A	319	HIS	-	expression tag	UNP Q6VVP7
A	320	HIS	-	expression tag	UNP Q6VVP7
A	321	HIS	-	expression tag	UNP Q6VVP7
A	322	HIS	-	expression tag	UNP Q6VVP7
A	323	HIS	-	expression tag	UNP Q6VVP7
A	324	HIS	-	expression tag	UNP Q6VVP7
B	314	ALA	-	expression tag	UNP Q6VVP7
B	315	ALA	-	expression tag	UNP Q6VVP7
B	316	ALA	-	expression tag	UNP Q6VVP7
B	317	LEU	-	expression tag	UNP Q6VVP7
B	318	GLU	-	expression tag	UNP Q6VVP7
B	319	HIS	-	expression tag	UNP Q6VVP7
B	320	HIS	-	expression tag	UNP Q6VVP7
B	321	HIS	-	expression tag	UNP Q6VVP7
B	322	HIS	-	expression tag	UNP Q6VVP7
B	323	HIS	-	expression tag	UNP Q6VVP7

Continued on next page...

Continued from previous page...

Chain	Residue	Modelled	Actual	Comment	Reference
B	324	HIS	-	expression tag	UNP Q6VVP7
C	314	ALA	-	expression tag	UNP Q6VVP7
C	315	ALA	-	expression tag	UNP Q6VVP7
C	316	ALA	-	expression tag	UNP Q6VVP7
C	317	LEU	-	expression tag	UNP Q6VVP7
C	318	GLU	-	expression tag	UNP Q6VVP7
C	319	HIS	-	expression tag	UNP Q6VVP7
C	320	HIS	-	expression tag	UNP Q6VVP7
C	321	HIS	-	expression tag	UNP Q6VVP7
C	322	HIS	-	expression tag	UNP Q6VVP7
C	323	HIS	-	expression tag	UNP Q6VVP7
C	324	HIS	-	expression tag	UNP Q6VVP7
D	314	ALA	-	expression tag	UNP Q6VVP7
D	315	ALA	-	expression tag	UNP Q6VVP7
D	316	ALA	-	expression tag	UNP Q6VVP7
D	317	LEU	-	expression tag	UNP Q6VVP7
D	318	GLU	-	expression tag	UNP Q6VVP7
D	319	HIS	-	expression tag	UNP Q6VVP7
D	320	HIS	-	expression tag	UNP Q6VVP7
D	321	HIS	-	expression tag	UNP Q6VVP7
D	322	HIS	-	expression tag	UNP Q6VVP7
D	323	HIS	-	expression tag	UNP Q6VVP7
D	324	HIS	-	expression tag	UNP Q6VVP7

- Molecule 2 is NICOTINAMIDE-ADENINE-DINUCLEOTIDE (three-letter code: NAD) (formula: $C_{21}H_{27}N_7O_{14}P_2$) (labeled as "Ligand of Interest" by author).

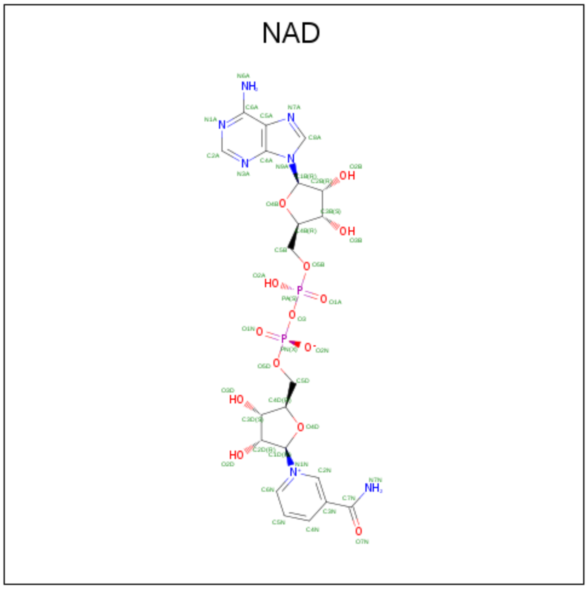

Mol	Chain	Residues	Atoms					ZeroOcc	AltConf
2	A	1	Total 44	C 21	N 7	O 14	P 2	0	0
2	B	1	Total 44	C 21	N 7	O 14	P 2	0	0
2	C	1	Total 44	C 21	N 7	O 14	P 2	0	0
2	D	1	Total 44	C 21	N 7	O 14	P 2	0	0

- Molecule 3 is water.

Mol	Chain	Residues	Atoms		ZeroOcc	AltConf
3	A	33	Total 33	O 33	0	0
3	B	35	Total 35	O 35	0	0
3	C	46	Total 46	O 46	0	0
3	D	29	Total 29	O 29	0	0

CONFIDENTIAL VALIDATION REPORT

3 Residue-property plots (i)

These plots are drawn for all protein, RNA and DNA chains in the entry. The first graphic for a chain summarises the proportions of the various outlier classes displayed in the second graphic. The second graphic shows the sequence view annotated by issues in geometry and electron density. Residues are color-coded according to the number of geometric quality criteria for which they contain at least one outlier: green = 0, yellow = 1, orange = 2 and red = 3 or more. A red dot above a residue indicates a poor fit to the electron density (RSRZ > 2). Stretches of 2 or more consecutive residues without any outlier are shown as a green connector. Residues present in the sample, but not in the model, are shown in grey.

• Molecule 1: Malate dehydrogenase

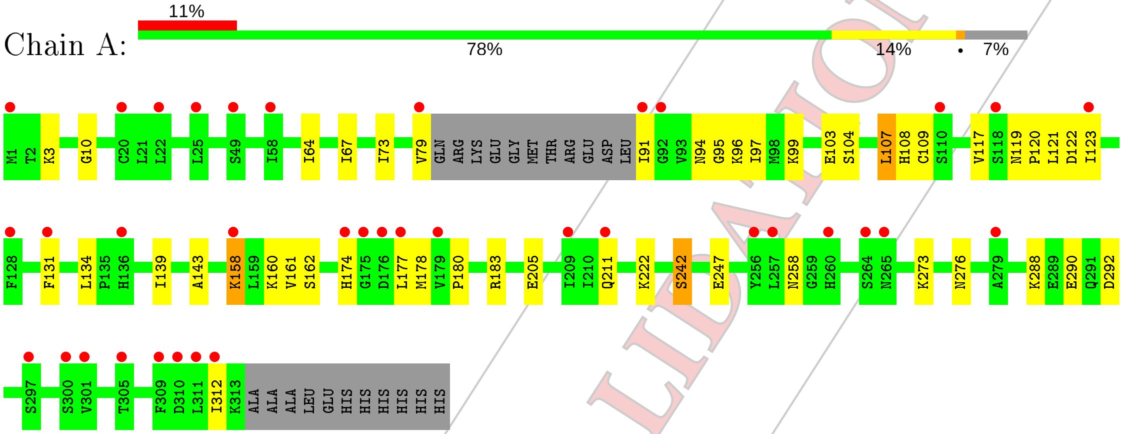

• Molecule 1: Malate dehydrogenase

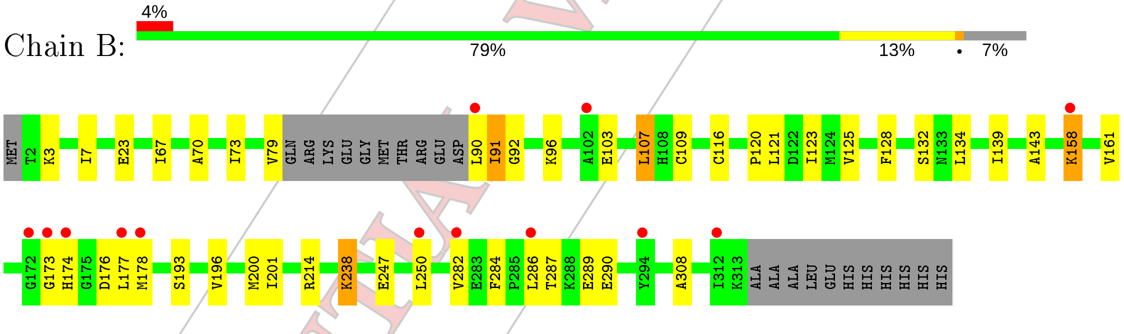

• Molecule 1: Malate dehydrogenase

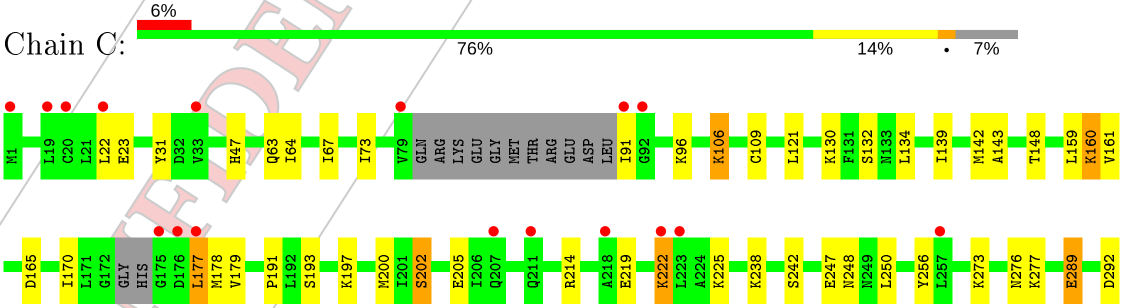

CONFIDENTIAL VALIDATION REPORT

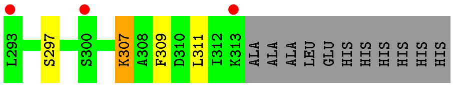

• Molecule 1: Malate dehydrogenase

Chain D:

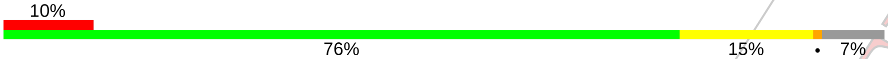

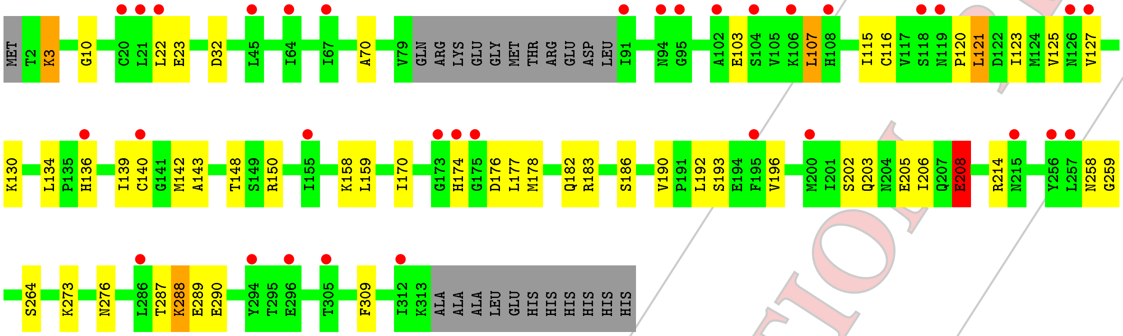

CONFIDENTIAL VALIDATION REPORT

4 Data and refinement statistics (i)

Property	Value	Source
Space group	P 21 21 21	Depositor
Cell constants<br>a, b, c, α, β, γ	84.62Å 107.42Å 145.08Å<br>90.00° 90.00° 90.00°	Depositor
Resolution (Å)	18.27 – 2.50<br>49.01 – 2.50	Depositor<br>EDS
% Data completeness<br>(in resolution range)	99.7 (18.27-2.50)<br>99.7 (49.01-2.50)	Depositor<br>EDS
R_{merge}	0.20	Depositor
R_{sym}	(Not available)	Depositor
$< I/\sigma(I) >$ [1]	1.55 (at 2.51Å)	Xtriage
Refinement program	PHENIX (dev_3753: ???)	Depositor
R, R_{free}	0.239 , 0.286<br>0.239 , 0.285	Depositor<br>DCC
R_{free} test set	2309 reflections (5.00%)	wwPDB-VP
Wilson B-factor (Å^2)	34.3	Xtriage
Anisotropy	0.412	Xtriage
Bulk solvent k_{sol}(e/Å^3), B_{sol}(Å^2)	0.36 , 53.1	EDS
L-test for twinning[2]	$< \|L\| > = 0.40$, $< L^2 > = 0.23$	Xtriage
Estimated twinning fraction	No twinning to report.	Xtriage
F_o,F_c correlation	0.88	EDS
Total number of atoms	9452	wwPDB-VP
Average B, all atoms (Å^2)	38.0	wwPDB-VP

Xtriage's analysis on translational NCS is as follows: *The largest off-origin peak in the Patterson function is 7.15% of the height of the origin peak. No significant pseudotranslation is detected.*

[1] Intensities estimated from amplitudes.

[2] Theoretical values of $< |L| >$, $< L^2 >$ for acentric reflections are 0.5, 0.333 respectively for untwinned datasets, and 0.375, 0.2 for perfectly twinned datasets.

CONFIDENTIAL VALIDATION REPORT

5 Model quality (i)

5.1 Standard geometry (i)

Bond lengths and bond angles in the following residue types are not validated in this section: NAD

The Z score for a bond length (or angle) is the number of standard deviations the observed value is removed from the expected value. A bond length (or angle) with $|Z| > 5$ is considered an outlier worth inspection. RMSZ is the root-mean-square of all Z scores of the bond lengths (or angles).

Mol	Chain	Bond lengths		Bond angles	
		RMSZ	#$\|Z\|$ >5	RMSZ	#$\|Z\|$ >5
1	A	0.35	0/2319	0.59	2/3134 (0.1%)
1	B	0.36	2/2322 (0.1%)	0.57	3/3139 (0.1%)
1	C	0.36	1/2304 (0.0%)	0.63	4/3112 (0.1%)
1	D	0.32	0/2314	0.62	4/3128 (0.1%)
All	All	0.35	3/9259 (0.0%)	0.60	13/12513 (0.1%)

Chiral center outliers are detected by calculating the chiral volume of a chiral center and verifying if the center is modelled as a planar moiety or with the opposite hand.A planarity outlier is detected by checking planarity of atoms in a peptide group, atoms in a mainchain group or atoms of a sidechain that are expected to be planar.

Mol	Chain	#Chirality outliers	#Planarity outliers
1	D	0	1

All (3) bond length outliers are listed below:

Mol	Chain	Res	Type	Atoms	Z	Observed(Å)	Ideal(Å)
1	C	160	LYS	CD-CE	6.26	1.67	1.51
1	B	158	LYS	CE-NZ	5.53	1.62	1.49
1	B	158	LYS	CD-CE	-5.27	1.38	1.51

All (13) bond angle outliers are listed below:

Mol	Chain	Res	Type	Atoms	Z	Observed(^{o})	Ideal(^{o})
1	D	177	LEU	CB-CG-CD2	-13.24	88.49	111.00
1	B	158	LYS	CD-CE-NZ	-9.44	89.99	111.70
1	D	177	LEU	CB-CG-CD1	9.03	126.36	111.00
1	A	177	LEU	CA-CB-CG	-7.13	98.91	115.30
1	A	158	LYS	CA-CB-CG	-6.64	98.79	113.40
1	D	208	GLU	CA-CB-CG	6.21	127.06	113.40

Continued on next page...

CONFIDENTIAL VALIDATION REPORT

Continued from previous page...

Mol	Chain	Res	Type	Atoms	Z	Observed(°)	Ideal(°)
1	B	158	LYS	CA-CB-CG	5.71	125.96	113.40
1	C	307	LYS	CB-CG-CD	5.51	125.94	111.60
1	B	158	LYS	CB-CA-C	5.43	121.26	110.40
1	D	208	GLU	CB-CA-C	-5.39	99.61	110.40
1	C	289	GLU	CA-CB-CG	5.16	124.74	113.40
1	C	177	LEU	CB-CG-CD1	-5.03	102.45	111.00
1	C	277	LYS	CB-CG-CD	-5.01	98.56	111.60

There are no chirality outliers.

All (1) planarity outliers are listed below:

Mol	Chain	Res	Type	Group
1	D	208	GLU	Sidechain

5.2 Too-close contacts (i)

In the following table, the Non-H and H(model) columns list the number of non-hydrogen atoms and hydrogen atoms in the chain respectively. The H(added) column lists the number of hydrogen atoms added and optimized by MolProbity. The Clashes column lists the number of clashes within the asymmetric unit, whereas Symm-Clashes lists symmetry related clashes.

Mol	Chain	Non-H	H(model)	H(added)	Clashes	Symm-Clashes
1	A	2287	0	2367	47	0
1	B	2290	0	2368	34	0
1	C	2274	0	2351	41	0
1	D	2282	0	2357	37	0
2	A	44	0	26	6	0
2	B	44	0	26	0	0
2	C	44	0	26	0	0
2	D	44	0	26	3	0
3	A	33	0	0	9	0
3	B	35	0	0	3	0
3	C	46	0	0	6	0
3	D	29	0	0	8	0
All	All	9452	0	9547	153	0

The all-atom clashscore is defined as the number of clashes found per 1000 atoms (including hydrogen atoms). The all-atom clashscore for this structure is 8.

All (153) close contacts within the same asymmetric unit are listed below, sorted by their clash magnitude.

CONFIDENTIAL VALIDATION REPORT

Atom-1	Atom-2	Interatomic distance (Å)	Clash overlap (Å)
1:A:158:LYS:NZ	1:A:205:GLU:OE1	1.86	1.07
1:A:211:GLN:OE1	3:A:501:HOH:O	1.86	0.93
1:A:119:ASN:OD1	1:A:121:LEU:N	2.00	0.92
1:C:307:LYS:HD2	1:C:311:LEU:HD11	1.52	0.92
1:A:158:LYS:NZ	1:A:205:GLU:CD	2.22	0.92
1:D:259:GLY:O	3:D:501:HOH:O	1.86	0.91
1:D:159:LEU:O	3:D:502:HOH:O	1.88	0.90
1:D:136:HIS:O	3:D:503:HOH:O	1.90	0.90
1:A:122:ASP:OD2	3:A:502:HOH:O	1.92	0.86
1:C:165:ASP:OD1	3:C:501:HOH:O	1.94	0.84
1:D:3:LYS:HG3	1:D:70:ALA:HA	1.59	0.84
1:C:165:ASP:OD2	3:C:502:HOH:O	1.94	0.84
1:A:121:LEU:HD13	2:A:401:NAD:N7N	1.95	0.82
1:A:121:LEU:HD13	2:A:401:NAD:H72N	1.47	0.80
1:D:158:LYS:HE3	1:D:205:GLU:OE2	1.82	0.79
1:C:177:LEU:HD12	1:C:297:SER:HA	1.66	0.78
1:D:23:GLU:OE2	3:D:504:HOH:O	2.02	0.77
1:B:23:GLU:OE2	3:B:501:HOH:O	2.03	0.76
1:D:121:LEU:HD13	2:D:401:NAD:H72N	1.50	0.76
1:D:148:THR:OG1	3:D:505:HOH:O	2.03	0.75
1:C:121:LEU:HD22	1:C:143:ALA:HB2	1.69	0.74
1:A:158:LYS:NZ	1:A:205:GLU:OE2	2.21	0.73
1:D:289:GLU:OE2	3:D:506:HOH:O	2.06	0.73
1:A:158:LYS:HZ1	1:A:205:GLU:CD	1.92	0.73
1:A:158:LYS:HE3	1:A:205:GLU:OE2	1.89	0.72
1:B:3:LYS:NZ	3:B:502:HOH:O	2.20	0.71
1:A:160:LYS:O	1:C:248:ASN:ND2	2.25	0.70
1:B:238:LYS:NZ	1:B:247:GLU:OE1	2.24	0.70
1:C:23:GLU:O	3:C:504:HOH:O	2.08	0.69
1:C:307:LYS:HD2	1:C:311:LEU:CD1	2.22	0.69
1:A:134:LEU:HB2	1:A:139:ILE:HD11	1.75	0.69
1:A:158:LYS:CE	1:A:205:GLU:OE2	2.41	0.68
1:A:162:SER:O	3:A:504:HOH:O	2.11	0.68
1:A:120:PRO:HB2	1:A:123:ILE:HD13	1.77	0.67
1:A:273:LYS:NZ	3:A:508:HOH:O	2.28	0.66
1:C:63:GLN:NE2	3:C:508:HOH:O	2.27	0.66
1:D:186:SER:OG	3:D:507:HOH:O	2.14	0.65
1:C:256:TYR:O	3:C:505:HOH:O	2.14	0.65
1:B:3:LYS:HG3	1:B:70:ALA:HA	1.78	0.65
1:B:121:LEU:HD22	1:B:143:ALA:HB2	1.78	0.65
1:C:178:MET:HE3	1:C:214:ARG:HA	1.80	0.64
1:A:158:LYS:HZ2	1:A:205:GLU:CD	1.93	0.63

Continued on next page...

CONFIDENTIAL VALIDATION REPORT

Continued from previous page...

Atom-1	Atom-2	Interatomic distance (Å)	Clash overlap (Å)
1:B:158:LYS:HD2	1:B:158:LYS:C	2.17	0.63
1:C:159:LEU:O	1:C:160:LYS:HB2	1.99	0.63
1:D:134:LEU:HB2	1:D:139:ILE:HD11	1.80	0.62
1:C:289:GLU:HA	1:C:292:ASP:HB2	1.82	0.62
1:C:219:GLU:OE1	1:C:222:LYS:NZ	2.32	0.62
1:B:250:LEU:HD21	1:B:282:VAL:HG21	1.82	0.61
1:D:143:ALA:HB1	1:D:174:HIS:HB2	1.83	0.60
1:B:134:LEU:HB2	1:B:139:ILE:HD11	1.85	0.59
1:C:73:ILE:HD11	1:C:109:CYS:SG	2.43	0.59
1:B:7:ILE:HD11	1:B:67:ILE:HD11	1.83	0.59
1:A:103:GLU:O	1:A:107:LEU:HD23	2.02	0.59
1:A:121:LEU:HD22	1:A:143:ALA:HB2	1.83	0.59
1:B:103:GLU:O	1:B:107:LEU:HD23	2.03	0.58
1:A:108:HIS:ND1	3:A:509:HOH:O	2.28	0.58
1:D:182:GLN:HE22	1:D:203:GLN:HE22	1.52	0.58
1:C:148:THR:HA	1:C:170:ILE:HD12	1.86	0.58
1:D:121:LEU:HD13	1:D:142:MET:O	2.03	0.58
1:D:178:MET:HE3	1:D:214:ARG:HA	1.86	0.58
1:C:219:GLU:O	1:C:219:GLU:HG3	2.05	0.57
1:C:134:LEU:HB2	1:C:139:ILE:HD11	1.86	0.57
1:D:121:LEU:HD13	2:D:401:NAD:N7N	2.20	0.56
1:C:238:LYS:NZ	3:C:511:HOH:O	2.27	0.55
1:B:143:ALA:HB1	1:B:174:HIS:HB2	1.90	0.54
1:B:176:ASP:OD1	1:B:214:ARG:HD3	2.08	0.54
1:A:161:VAL:HA	1:C:248:ASN:HD22	1.70	0.54
1:A:64:ILE:O	1:A:67:ILE:HG12	2.06	0.54
1:C:64:ILE:O	1:C:67:ILE:HG12	2.07	0.54
1:D:158:LYS:HE2	1:D:205:GLU:HB3	1.88	0.54
1:B:173:GLY:HA3	1:B:177:LEU:CD1	2.37	0.53
1:B:178:MET:HE3	1:B:214:ARG:HA	1.91	0.53
1:D:176:ASP:HA	1:D:214:ARG:HD3	1.91	0.52
1:A:79:VAL:H	1:A:94:ASN:HD21	1.57	0.52
1:A:97:ILE:HG12	2:A:401:NAD:N6A	2.25	0.52
1:A:273:LYS:HE2	1:C:161:VAL:HG12	1.91	0.52
1:B:196:VAL:HG12	1:B:201:ILE:HG23	1.91	0.52
1:A:143:ALA:HB1	1:A:174:HIS:HB2	1.91	0.52
1:D:103:GLU:O	1:D:107:LEU:HD23	2.10	0.51
1:B:116:CYS:HB2	1:B:125:VAL:HG22	1.92	0.51
1:B:158:LYS:NZ	1:B:200:MET:O	2.40	0.51
3:A:508:HOH:O	1:C:200:MET:HE1	2.10	0.51
1:B:173:GLY:HA3	1:B:177:LEU:HD11	1.92	0.51

Continued on next page...

CONFIDENTIAL VALIDATION REPORT

Continued from previous page...

Atom-1	Atom-2	Interatomic distance (Å)	Clash overlap (Å)
1:B:73:ILE:HD11	1:B:109:CYS:SG	2.51	0.50
1:D:258:ASN:OD1	1:D:264:SER:OG	2.28	0.50
1:A:292:ASP:OD2	3:A:505:HOH:O	2.20	0.50
1:A:73:ILE:HD11	1:A:109:CYS:SG	2.52	0.50
1:A:117:VAL:O	2:A:401:NAD:H2N	2.11	0.50
1:B:161:VAL:HG12	1:D:273:LYS:HD3	1.94	0.50
1:D:287:THR:HG22	1:D:288:LYS:N	2.27	0.50
1:C:177:LEU:HD12	1:C:297:SER:CA	2.40	0.49
1:D:148:THR:HA	1:D:170:ILE:HD12	1.94	0.49
1:C:307:LYS:O	1:C:311:LEU:HG	2.13	0.49
1:B:120:PRO:HB2	1:B:123:ILE:HD13	1.95	0.49
1:A:119:ASN:OD1	1:A:120:PRO:HA	2.12	0.48
1:D:120:PRO:HB2	1:D:123:ILE:HD13	1.97	0.47
1:B:128:PHE:O	1:B:132:SER:HB3	2.15	0.47
1:B:79:VAL:O	1:B:90:LEU:HD13	2.15	0.47
1:D:287:THR:HG22	1:D:288:LYS:H	1.79	0.47
1:B:158:LYS:HG3	1:B:201:ILE:HD12	1.96	0.47
1:B:287:THR:OG1	1:B:289:GLU:HG2	2.15	0.46
1:D:116:CYS:HB2	1:D:125:VAL:HG22	1.98	0.46
1:A:95:GLY:HA3	1:A:312:ILE:HD11	1.97	0.46
1:C:130:LYS:HD3	1:C:309:PHE:CE1	2.51	0.46
1:C:106:LYS:HB3	1:C:132:SER:O	2.16	0.46
1:B:238:LYS:HD2	1:B:238:LYS:O	2.16	0.45
1:D:143:ALA:CB	1:D:174:HIS:HB2	2.46	0.45
1:A:104:SER:OG	3:A:503:HOH:O	2.04	0.45
1:B:90:LEU:O	1:B:92:GLY:N	2.50	0.45
1:B:91:ILE:HG22	3:B:534:HOH:O	2.17	0.45
1:C:307:LYS:HD2	1:C:311:LEU:CG	2.47	0.45
1:D:127:VAL:HG22	1:D:309:PHE:HE1	1.82	0.44
1:C:47:HIS:CE1	1:D:150:ARG:HG2	2.52	0.44
1:A:96:LYS:C	1:A:96:LYS:HD3	2.38	0.44
1:C:170:ILE:HA	1:C:179:VAL:O	2.18	0.44
1:C:250:LEU:CD1	1:C:273:LYS:HG2	2.48	0.43
1:A:288:LYS:HE3	1:A:292:ASP:OD2	2.17	0.43
1:A:258:ASN:HD22	1:A:258:ASN:HA	1.67	0.43
1:C:238:LYS:HD2	1:C:238:LYS:HA	1.76	0.43
1:A:91:ILE:HG12	1:A:120:PRO:HG3	1.99	0.43
1:A:99:LYS:HG3	1:A:131:PHE:CG	2.53	0.43
1:D:193:SER:O	1:D:196:VAL:HG22	2.18	0.43
1:C:22:LEU:HD13	1:D:22:LEU:HD13	2.01	0.43
1:A:99:LYS:HG3	1:A:131:PHE:CD2	2.54	0.43

Continued on next page...

CONFIDENTIAL VALIDATION REPORT

Continued from previous page...

Atom-1	Atom-2	Interatomic distance (Å)	Clash overlap (Å)
1:D:115:ILE:HG12	1:D:140:CYS:SG	2.59	0.42
1:D:10:GLY:HA3	2:D:401:NAD:O5B	2.19	0.42
1:C:96:LYS:HE3	1:C:96:LYS:HB3	1.65	0.42
1:C:202:SER:N	1:C:205:GLU:OE2	2.52	0.42
1:A:143:ALA:HB3	3:A:527:HOH:O	2.19	0.42
1:D:192:LEU:HD21	1:D:206:ILE:HD13	2.01	0.42
1:C:250:LEU:HD13	1:C:273:LYS:HG2	2.00	0.42
1:A:242:SER:HA	1:A:247:GLU:HB2	2.02	0.42
1:A:3:LYS:HB3	1:A:3:LYS:HE3	1.80	0.42
1:B:284:PHE:CE2	1:D:190:VAL:HG21	2.55	0.42
1:D:183:ARG:HD3	1:D:290:GLU:OE2	2.19	0.42
1:B:286:LEU:HG	1:B:290:GLU:HB3	2.01	0.41
1:B:91:ILE:HD11	1:B:308:ALA:HB2	2.00	0.41
1:C:191:PRO:HB2	1:C:193:SER:OG	2.20	0.41
1:C:193:SER:O	1:C:197:LYS:HG3	2.20	0.41
1:A:158:LYS:CE	1:A:205:GLU:CD	2.85	0.41
1:A:10:GLY:HA3	2:A:401:NAD:O5B	2.21	0.41
1:B:193:SER:O	1:B:196:VAL:HG22	2.20	0.41
1:C:242:SER:HA	1:C:247:GLU:HB2	2.02	0.41
1:D:143:ALA:HB3	3:D:518:HOH:O	2.19	0.41
1:A:183:ARG:HD3	1:A:290:GLU:OE2	2.20	0.41
1:A:79:VAL:H	1:A:94:ASN:ND2	2.18	0.41
2:A:401:NAD:H6N	2:A:401:NAD:H2D	1.89	0.41
1:B:67:ILE:HG21	1:B:67:ILE:HD13	1.72	0.41
1:A:178:MET:HE3	1:A:180:PRO:HG3	2.02	0.41
1:B:158:LYS:O	1:B:158:LYS:HD2	2.21	0.41
1:C:219:GLU:CD	1:C:222:LYS:HZ1	2.23	0.41
1:A:222:LYS:HB3	1:A:222:LYS:HE3	1.90	0.40
1:C:31:TYR:CE2	1:C:64:ILE:HD12	2.57	0.40

There are no symmetry-related clashes.

5.3 Torsion angles (i)

5.3.1 Protein backbone (i)

In the following table, the Percentiles column shows the percent Ramachandran outliers of the chain as a percentile score with respect to all X-ray entries followed by that with respect to entries of similar resolution.

The Analysed column shows the number of residues for which the backbone conformation was analysed, and the total number of residues.

CONFIDENTIAL VALIDATION REPORT

Mol	Chain	Analysed	Favoured	Allowed	Outliers	Percentiles	
1	A	298/324 (92%)	286 (96%)	11 (4%)	1 (0%)	43	64
1	B	298/324 (92%)	283 (95%)	14 (5%)	1 (0%)	43	64
1	C	294/324 (91%)	282 (96%)	11 (4%)	1 (0%)	43	64
1	D	297/324 (92%)	285 (96%)	11 (4%)	1 (0%)	43	64
All	All	1187/1296 (92%)	1136 (96%)	47 (4%)	4 (0%)	43	64

All (4) Ramachandran outliers are listed below:

Mol	Chain	Res	Type
1	B	91	ILE
1	C	276	ASN
1	D	276	ASN
1	A	276	ASN

5.3.2 Protein sidechains (i)

In the following table, the Percentiles column shows the percent sidechain outliers of the chain as a percentile score with respect to all X-ray entries followed by that with respect to entries of similar resolution.

The Analysed column shows the number of residues for which the sidechain conformation was analysed, and the total number of residues.

Mol	Chain	Analysed	Rotameric	Outliers	Percentiles	
1	A	254/273 (93%)	252 (99%)	2 (1%)	83	94
1	B	255/273 (93%)	252 (99%)	3 (1%)	74	90
1	C	253/273 (93%)	247 (98%)	6 (2%)	52	78
1	D	254/273 (93%)	246 (97%)	8 (3%)	43	70
All	All	1016/1092 (93%)	997 (98%)	19 (2%)	60	83

All (19) residues with a non-rotameric sidechain are listed below:

Mol	Chain	Res	Type
1	A	107	LEU
1	A	242	SER
1	B	96	LYS
1	B	107	LEU
1	B	238	LYS
1	C	91	ILE
1	C	106	LYS

Continued on next page...

CONFIDENTIAL VALIDATION REPORT

Continued from previous page...

Mol	Chain	Res	Type
1	C	142	MET
1	C	202	SER
1	C	222	LYS
1	C	225	LYS
1	D	3	LYS
1	D	32	ASP
1	D	107	LEU
1	D	121	LEU
1	D	130	LYS
1	D	202	SER
1	D	208	GLU
1	D	288	LYS

Some sidechains can be flipped to improve hydrogen bonding and reduce clashes. All (5) such sidechains are listed below:

Mol	Chain	Res	Type
1	A	258	ASN
1	C	204	ASN
1	C	249	ASN
1	D	55	ASN
1	D	203	GLN

5.3.3 RNA (i)

There are no RNA molecules in this entry.

5.4 Non-standard residues in protein, DNA, RNA chains (i)

There are no non-standard protein/DNA/RNA residues in this entry.

5.5 Carbohydrates (i)

There are no carbohydrates in this entry.

5.6 Ligand geometry (i)

4 ligands are modelled in this entry.

CONFIDENTIAL VALIDATION REPORT

In the following table, the Counts columns list the number of bonds (or angles) for which Mogul statistics could be retrieved, the number of bonds (or angles) that are observed in the model and the number of bonds (or angles) that are defined in the Chemical Component Dictionary. The Link column lists molecule types, if any, to which the group is linked. The Z score for a bond length (or angle) is the number of standard deviations the observed value is removed from the expected value. A bond length (or angle) with $|Z| > 2$ is considered an outlier worth inspection. RMSZ is the root-mean-square of all Z scores of the bond lengths (or angles).

Mol	Type	Chain	Res	Link	Bond lengths			Bond angles		
					Counts	RMSZ	$\#\|Z\| > 2$	Counts	RMSZ	$\#\|Z\| > 2$
2	NAD	D	401	-	39,48,48	4.31	19 (48%)	44,73,73	2.21	7 (15%)
2	NAD	C	401	-	39,48,48	4.31	19 (48%)	44,73,73	2.18	7 (15%)
2	NAD	B	401	-	39,48,48	4.32	18 (46%)	44,73,73	2.21	8 (18%)
2	NAD	A	401	-	39,48,48	4.42	18 (46%)	44,73,73	2.18	8 (18%)

In the following table, the Chirals column lists the number of chiral outliers, the number of chiral centers analysed, the number of these observed in the model and the number defined in the Chemical Component Dictionary. Similar counts are reported in the Torsion and Rings columns. '-' means no outliers of that kind were identified.

Mol	Type	Chain	Res	Link	Chirals	Torsions	Rings
2	NAD	D	401	-	-	1/22/62/62	0/5/5/5
2	NAD	C	401	-	-	2/22/62/62	0/5/5/5
2	NAD	B	401	-	-	1/22/62/62	0/5/5/5
2	NAD	A	401	-	-	1/22/62/62	0/5/5/5

All (74) bond length outliers are listed below:

Mol	Chain	Res	Type	Atoms	Z	Observed(Å)	Ideal(Å)
2	A	401	NAD	C2N-C3N	12.63	1.58	1.39
2	C	401	NAD	C2N-C3N	12.27	1.58	1.39
2	B	401	NAD	C2N-C3N	12.27	1.58	1.39
2	A	401	NAD	C4A-N3A	-12.20	1.18	1.35
2	B	401	NAD	C4A-N3A	-12.15	1.18	1.35
2	D	401	NAD	C4A-N3A	-12.10	1.19	1.35
2	C	401	NAD	C4A-N3A	-12.02	1.19	1.35
2	D	401	NAD	C2N-C3N	11.98	1.57	1.39
2	D	401	NAD	C8A-N7A	-9.86	1.17	1.34
2	C	401	NAD	C8A-N7A	-9.85	1.17	1.34
2	A	401	NAD	C8A-N7A	-9.84	1.17	1.34
2	B	401	NAD	C8A-N7A	-9.80	1.17	1.34
2	A	401	NAD	C6N-C5N	8.27	1.57	1.38
2	A	401	NAD	O4D-C1D	7.93	1.52	1.41

Continued on next page...

CONFIDENTIAL VALIDATION REPORT

Continued from previous page...

Mol	Chain	Res	Type	Atoms	Z	Observed(Å)	Ideal(Å)
2	B	401	NAD	C7N-N7N	7.93	1.47	1.33
2	B	401	NAD	C6N-C5N	7.90	1.56	1.38
2	C	401	NAD	C7N-N7N	7.89	1.47	1.33
2	D	401	NAD	C7N-N7N	7.87	1.47	1.33
2	C	401	NAD	C6N-C5N	7.86	1.56	1.38
2	D	401	NAD	C6N-C5N	7.84	1.56	1.38
2	A	401	NAD	C7N-N7N	7.81	1.47	1.33
2	D	401	NAD	O4D-C1D	6.92	1.50	1.41
2	B	401	NAD	O4D-C1D	6.72	1.50	1.41
2	C	401	NAD	O4D-C1D	6.68	1.50	1.41
2	D	401	NAD	O4B-C1B	6.39	1.50	1.41
2	B	401	NAD	O4B-C1B	6.34	1.50	1.41
2	C	401	NAD	O4B-C1B	6.34	1.50	1.41
2	A	401	NAD	O4B-C1B	6.25	1.50	1.41
2	C	401	NAD	C5A-C4A	4.33	1.50	1.40
2	D	401	NAD	C5A-C4A	4.32	1.50	1.40
2	A	401	NAD	C5A-C4A	4.29	1.50	1.40
2	B	401	NAD	C5A-C4A	4.23	1.50	1.40
2	A	401	NAD	C2D-C3D	-3.62	1.44	1.53
2	B	401	NAD	C2A-N3A	-3.58	1.26	1.32
2	A	401	NAD	C2A-N3A	-3.53	1.26	1.32
2	D	401	NAD	C2A-N3A	-3.51	1.26	1.32
2	C	401	NAD	C2A-N3A	-3.46	1.26	1.32
2	C	401	NAD	C2D-C3D	-3.46	1.44	1.53
2	D	401	NAD	C2D-C3D	-3.43	1.44	1.53
2	D	401	NAD	C2B-C3B	-3.30	1.44	1.53
2	B	401	NAD	C2D-C3D	-3.28	1.44	1.53
2	A	401	NAD	C3N-C7N	3.25	1.55	1.50
2	B	401	NAD	C2B-C3B	-3.24	1.45	1.53
2	D	401	NAD	C6A-N6A	3.23	1.46	1.34
2	C	401	NAD	C2B-C3B	-3.21	1.45	1.53
2	A	401	NAD	C6A-N6A	3.20	1.46	1.34
2	A	401	NAD	C2B-C3B	-3.20	1.45	1.53
2	C	401	NAD	C6A-N6A	3.17	1.46	1.34
2	B	401	NAD	C6A-N6A	3.16	1.46	1.34
2	C	401	NAD	C3N-C7N	3.09	1.55	1.50
2	B	401	NAD	C3N-C7N	3.08	1.55	1.50
2	D	401	NAD	C3N-C7N	2.87	1.54	1.50
2	D	401	NAD	O7N-C7N	-2.65	1.19	1.24
2	B	401	NAD	O7N-C7N	-2.62	1.19	1.24
2	C	401	NAD	O7N-C7N	-2.52	1.19	1.24
2	D	401	NAD	O4D-C4D	2.40	1.50	1.45

Continued on next page...

CONFIDENTIAL VALIDATION REPORT

Continued from previous page...

Mol	Chain	Res	Type	Atoms	Z	Observed(Å)	Ideal(Å)
2	A	401	NAD	O4D-C4D	2.40	1.50	1.45
2	A	401	NAD	O7N-C7N	-2.39	1.19	1.24
2	C	401	NAD	O4B-C4B	2.29	1.50	1.45
2	C	401	NAD	O4D-C4D	2.17	1.49	1.45
2	D	401	NAD	C6A-N1A	2.16	1.46	1.37
2	C	401	NAD	PA-O2A	-2.13	1.45	1.55
2	B	401	NAD	C6A-N1A	2.12	1.46	1.37
2	B	401	NAD	PA-O2A	-2.12	1.45	1.55
2	D	401	NAD	PN-O5D	2.11	1.67	1.59
2	A	401	NAD	PA-O2A	-2.11	1.45	1.55
2	A	401	NAD	C6A-N1A	2.11	1.46	1.37
2	C	401	NAD	C6A-N1A	2.10	1.46	1.37
2	D	401	NAD	PA-O2A	-2.09	1.45	1.55
2	D	401	NAD	O4B-C4B	2.08	1.49	1.45
2	C	401	NAD	PN-O5D	2.07	1.67	1.59
2	A	401	NAD	O4B-C4B	2.05	1.49	1.45
2	B	401	NAD	O4D-C4D	2.05	1.49	1.45
2	B	401	NAD	O4B-C4B	2.03	1.49	1.45

All (30) bond angle outliers are listed below:

Mol	Chain	Res	Type	Atoms	Z	Observed(^{o})	Ideal(^{o})
2	C	401	NAD	C4B-O4B-C1B	-9.22	100.22	109.83
2	B	401	NAD	C4B-O4B-C1B	-9.17	100.27	109.83
2	D	401	NAD	C4B-O4B-C1B	-8.78	100.68	109.83
2	A	401	NAD	C4B-O4B-C1B	-8.62	100.84	109.83
2	D	401	NAD	C4D-O4D-C1D	-7.53	101.97	109.83
2	C	401	NAD	C4D-O4D-C1D	-6.81	102.72	109.83
2	A	401	NAD	C4D-O4D-C1D	-6.19	103.37	109.83
2	B	401	NAD	C4D-O4D-C1D	-5.97	103.60	109.83
2	B	401	NAD	C1B-N9A-C4A	-4.79	118.37	126.64
2	A	401	NAD	C1B-N9A-C4A	-4.61	118.67	126.64
2	D	401	NAD	C1B-N9A-C4A	-4.53	118.81	126.64
2	C	401	NAD	C1B-N9A-C4A	-3.87	119.95	126.64
2	D	401	NAD	C4A-C5A-N7A	3.61	113.17	109.40
2	C	401	NAD	C4A-C5A-N7A	3.52	113.07	109.40
2	A	401	NAD	C4A-C5A-N7A	3.46	113.01	109.40
2	B	401	NAD	C4A-C5A-N7A	3.42	112.97	109.40
2	B	401	NAD	PN-O3-PA	-2.87	123.45	132.57
2	A	401	NAD	C5D-C4D-C3D	-2.61	105.38	115.21
2	C	401	NAD	PN-O3-PA	-2.34	125.12	132.57
2	A	401	NAD	C2B-C3B-C4B	2.32	107.04	102.60

Continued on next page...

CONFIDENTIAL VALIDATION REPORT

Continued from previous page...

Mol	Chain	Res	Type	Atoms	Z	Observed(^{o})	Ideal(^{o})
2	A	401	NAD	PN-O3-PA	-2.20	125.57	132.57
2	B	401	NAD	C2B-C3B-C4B	2.20	106.82	102.60
2	B	401	NAD	N3A-C2A-N1A	-2.16	125.19	128.68
2	D	401	NAD	N3A-C2A-N1A	-2.15	125.22	128.68
2	D	401	NAD	PN-O3-PA	-2.13	125.80	132.57
2	A	401	NAD	N3A-C2A-N1A	-2.12	125.26	128.68
2	D	401	NAD	C2B-C3B-C4B	2.10	106.62	102.60
2	B	401	NAD	O2A-PA-O1A	-2.06	101.87	112.21
2	C	401	NAD	O2A-PA-O1A	-2.04	101.97	112.21
2	C	401	NAD	N3A-C2A-N1A	-2.01	125.43	128.68

There are no chirality outliers.

All (5) torsion outliers are listed below:

Mol	Chain	Res	Type	Atoms
2	A	401	NAD	O4B-C4B-C5B-O5B
2	B	401	NAD	O4B-C4B-C5B-O5B
2	C	401	NAD	O4B-C4B-C5B-O5B
2	D	401	NAD	O4B-C4B-C5B-O5B
2	C	401	NAD	C5D-O5D-PN-O1N

There are no ring outliers.

2 monomers are involved in 9 short contacts:

Mol	Chain	Res	Type	Clashes	Symm-Clashes
2	D	401	NAD	3	0
2	A	401	NAD	6	0

The following is a two-dimensional graphical depiction of Mogul quality analysis of bond lengths, bond angles, torsion angles, and ring geometry for all instances of the Ligand of Interest. In addition, ligands with molecular weight > 250 and outliers as shown on the validation Tables will also be included. For torsion angles, if less then 5% of the Mogul distribution of torsion angles is within 10 degrees of the torsion angle in question, then that torsion angle is considered an outlier. Any bond that is central to one or more torsion angles identified as an outlier by Mogul will be highlighted in the graph. For rings, the root-mean-square deviation (RMSD) between the ring in question and similar rings identified by Mogul is calculated over all ring torsion angles. If the average RMSD is greater than 60 degrees and the minimal RMSD between the ring in question and any Mogul-identified rings is also greater than 60 degrees, then that ring is considered an outlier. The outliers are highlighted in purple. The color gray indicates Mogul did not find sufficient equivalents in the CSD to analyse the geometry.

CONFIDENTIAL VALIDATION REPORT

Ligand NAD D 401

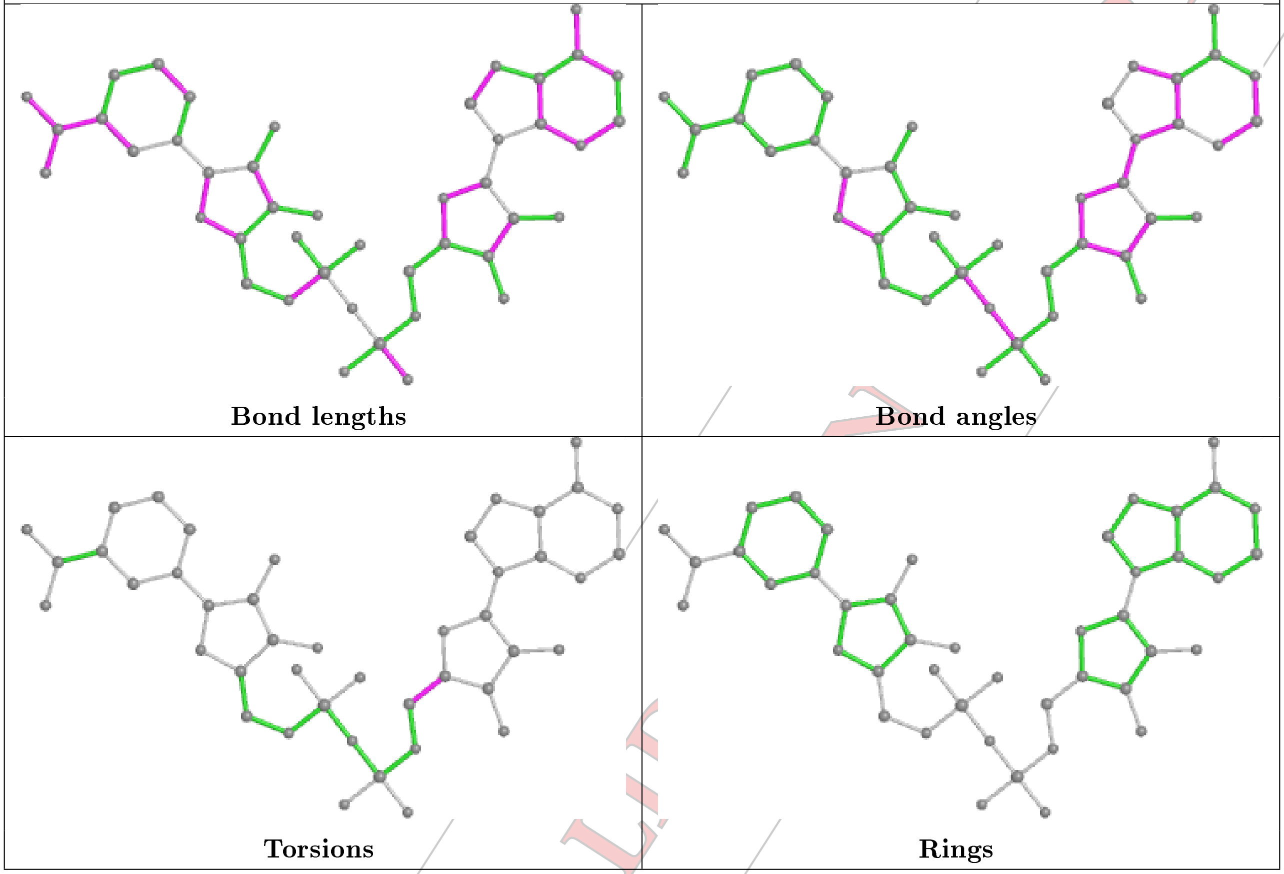

CONFIDENTIAL VALIDATION REPORT

Ligand NAD C 401

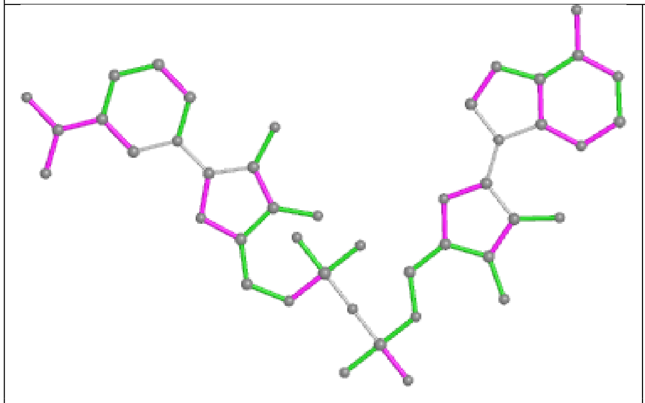

Bond lengths

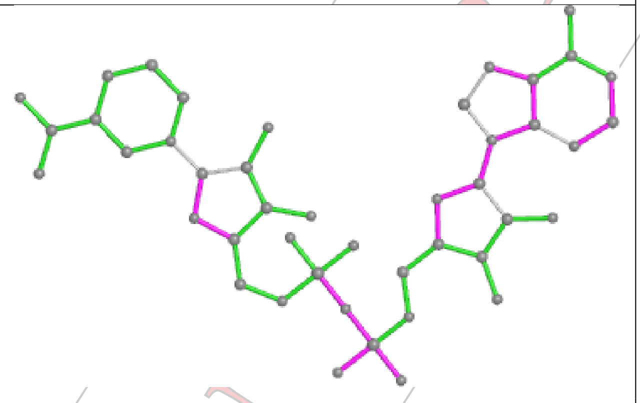

Bond angles

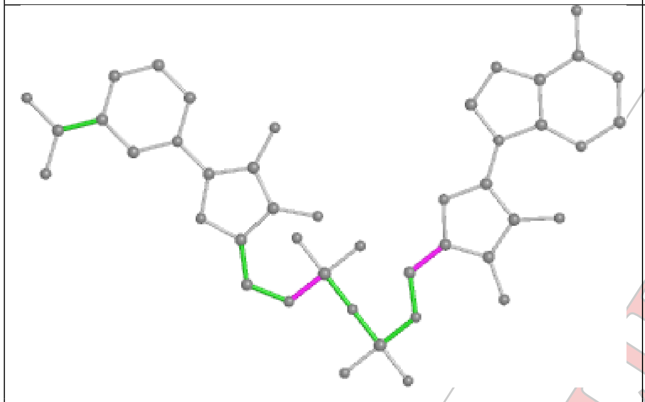

Torsions

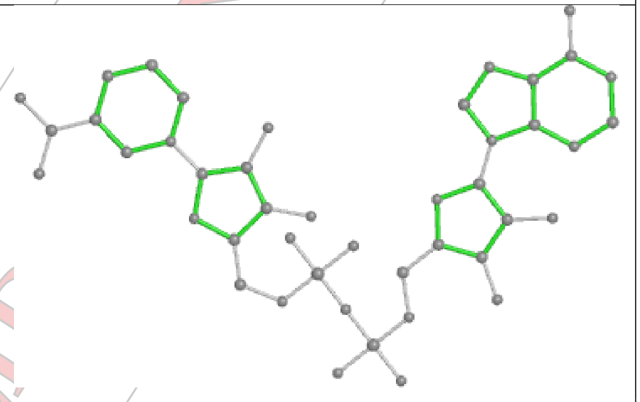

Rings

CONFIDENTIAL VAL

Ligand NAD B 401

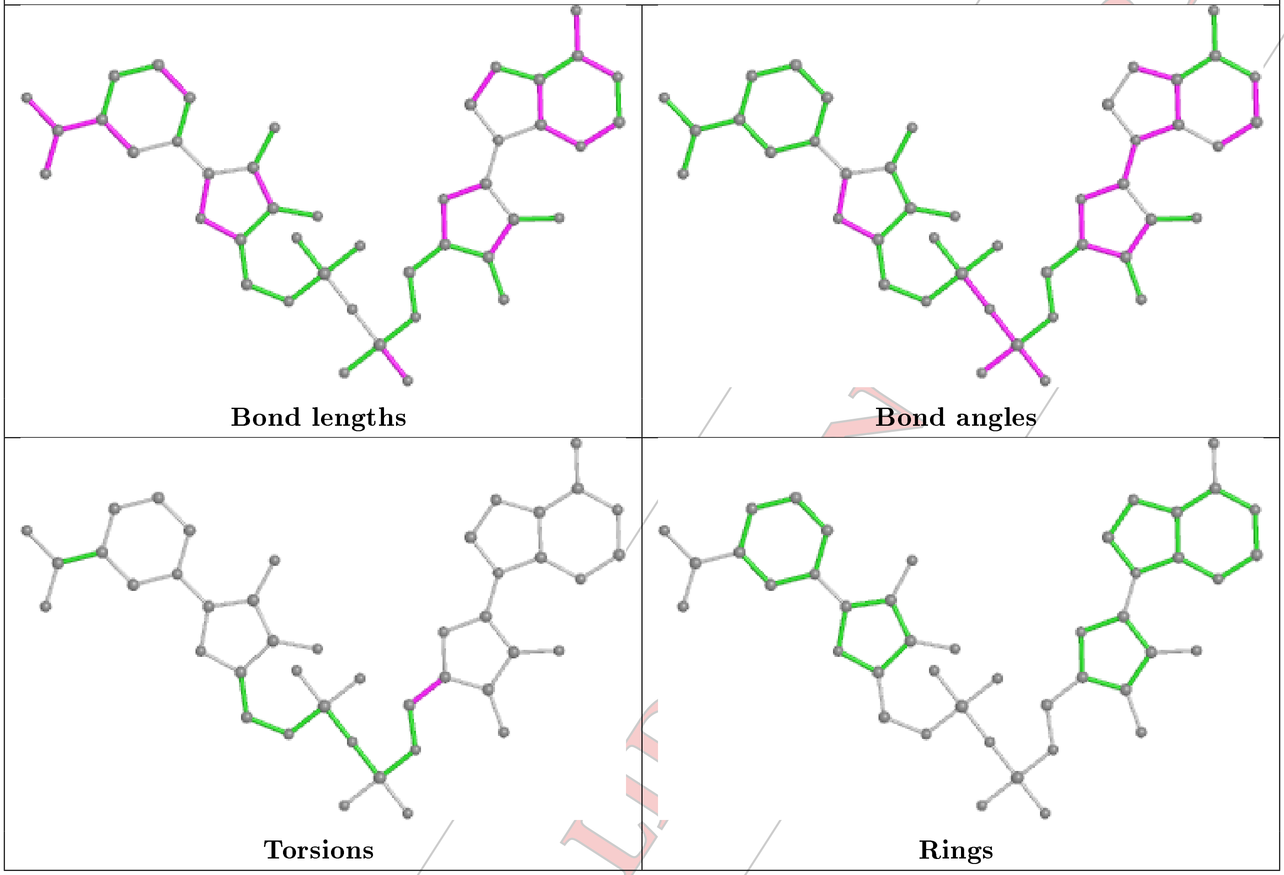

Ligand NAD A 401

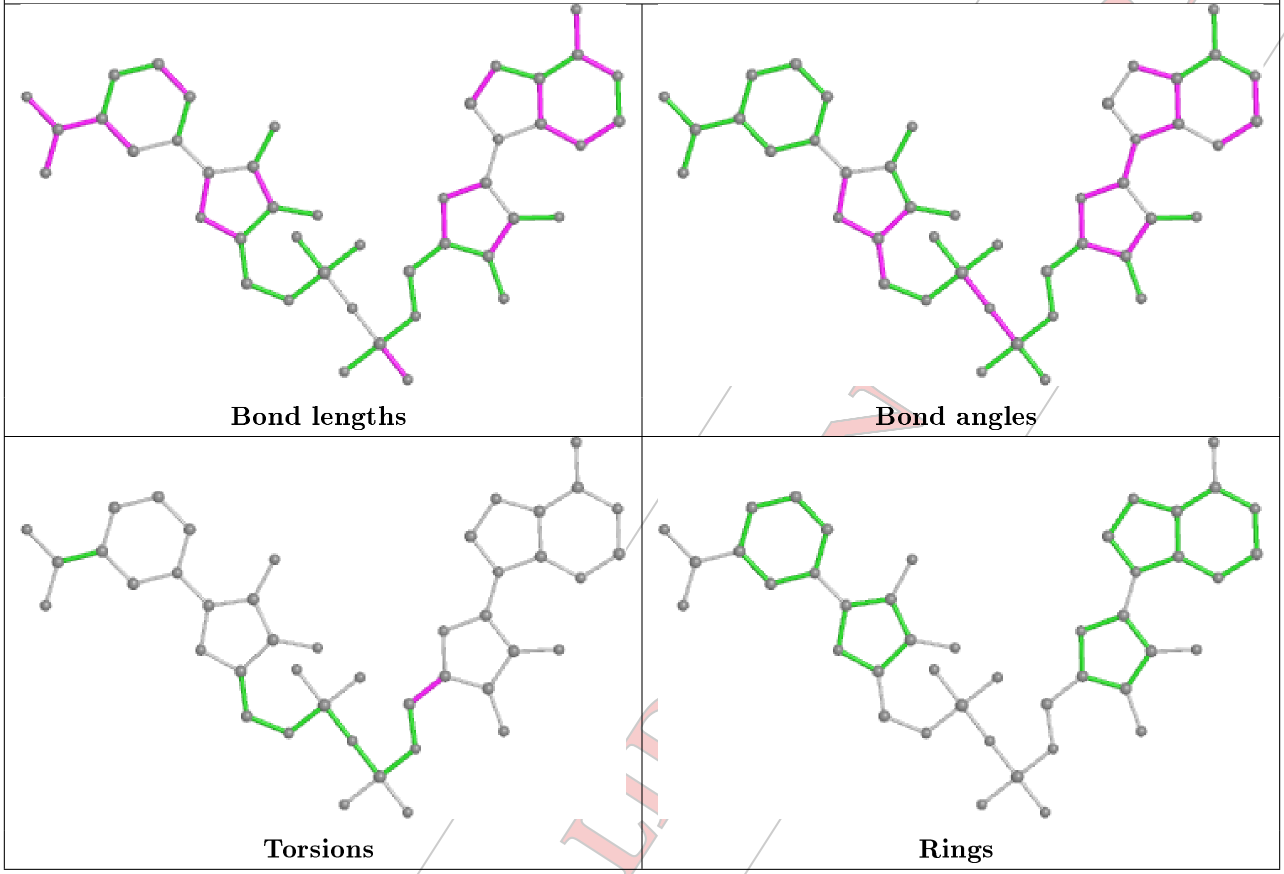

5.7 Other polymers (i)

There are no such residues in this entry.

5.8 Polymer linkage issues (i)

There are no chain breaks in this entry.

6 Fit of model and data (i)

6.1 Protein, DNA and RNA chains (i)

In the following table, the column labelled '#RSRZ> 2' contains the number (and percentage) of RSRZ outliers, followed by percent RSRZ outliers for the chain as percentile scores relative to all X-ray entries and entries of similar resolution. The OWAB column contains the minimum, median, 95th percentile and maximum values of the occupancy-weighted average B-factor per residue. The column labelled 'Q< 0.9' lists the number of (and percentage) of residues with an average occupancy less than 0.9.

Mol	Chain	Analysed	<RSRZ>	#RSRZ>2			OWAB(Å^2)	Q<0.9
1	A	302/324 (93%)	0.86	37 (12%)	4	3	19, 39, 67, 86	0
1	B	302/324 (93%)	0.48	13 (4%)	35	38	16, 31, 57, 91	0
1	C	300/324 (92%)	0.65	20 (6%)	18	18	15, 34, 63, 96	0
1	D	301/324 (92%)	0.76	33 (10%)	5	5	17, 36, 58, 78	0
All	All	1205/1296 (92%)	0.69	103 (8%)	11	11	15, 35, 63, 96	0

All (103) RSRZ outliers are listed below:

Mol	Chain	Res	Type	RSRZ
1	A	312	ILE	7.5
1	A	91	ILE	6.3
1	D	20	CYS	5.9
1	A	158	LYS	5.6
1	C	91	ILE	4.6
1	A	310	ASP	4.2
1	D	102	ALA	4.2
1	D	108	HIS	4.1
1	C	1	MET	4.0
1	B	173	GLY	3.9
1	A	20	CYS	3.9
1	A	174	HIS	3.7
1	C	175	GLY	3.7
1	D	67	ILE	3.7
1	C	22	LEU	3.6
1	D	296	GLU	3.5
1	D	175	GLY	3.5
1	B	90	LEU	3.5
1	A	260	HIS	3.5
1	D	305	THR	3.4
1	A	265	ASN	3.4

Continued on next page...

CONFIDENTIAL VALIDATION REPORT

Continued from previous page...

Mol	Chain	Res	Type	RSRZ
1	B	177	LEU	3.4
1	A	22	LEU	3.2
1	D	106	LYS	3.2
1	A	79	VAL	3.1
1	C	218	ALA	3.1
1	A	256	TYR	3.1
1	C	257	LEU	3.0
1	C	207	GLN	3.0
1	D	91	ILE	3.0
1	B	174	HIS	3.0
1	A	136	HIS	3.0
1	A	176	ASP	2.9
1	D	174	HIS	2.9
1	B	178	MET	2.8
1	D	173	GLY	2.8
1	A	92	GLY	2.8
1	C	313	LYS	2.8
1	A	177	LEU	2.8
1	D	94	ASN	2.8
1	A	123	ILE	2.8
1	D	22	LEU	2.8
1	A	297	SER	2.8
1	D	127	VAL	2.7
1	D	312	ILE	2.7
1	A	49	SER	2.7
1	A	264	SER	2.7
1	C	79	VAL	2.6
1	A	110	SER	2.6
1	A	301	VAL	2.6
1	B	172	GLY	2.6
1	D	286	LEU	2.6
1	B	158	LYS	2.6
1	A	211	GLN	2.6
1	D	257	LEU	2.6
1	B	102	ALA	2.5
1	A	305	THR	2.5
1	A	131	PHE	2.5
1	D	136	HIS	2.5
1	C	222	LYS	2.5
1	A	257	LEU	2.5
1	C	293	LEU	2.5
1	A	309	PHE	2.5

Continued on next page...

CONFIDENTIAL VALIDATION REPORT

Continued from previous page...

Mol	Chain	Res	Type	RSRZ
1	D	294	TYR	2.5
1	D	64	ILE	2.4
1	A	58	ILE	2.4
1	D	200	MET	2.4
1	C	20	CYS	2.4
1	A	25	LEU	2.4
1	C	223	LEU	2.4
1	D	119	ASN	2.3
1	D	95	GLY	2.3
1	D	126	ASN	2.3
1	A	311	LEU	2.3
1	D	45	LEU	2.3
1	B	294	TYR	2.3
1	A	175	GLY	2.2
1	A	300	SER	2.2
1	D	104	SER	2.2
1	C	19	LEU	2.2
1	A	179	VAL	2.2
1	C	211	GLN	2.2
1	C	92	GLY	2.2
1	A	279	ALA	2.2
1	B	286	LEU	2.2
1	C	300	SER	2.1
1	D	256	TYR	2.1
1	D	155	ILE	2.1
1	A	128	PHE	2.1
1	B	250	LEU	2.1
1	D	195	PHE	2.1
1	A	118	SER	2.1
1	B	282	VAL	2.1
1	D	21	LEU	2.1
1	D	118	SER	2.1
1	D	215	ASN	2.1
1	A	209	ILE	2.0
1	C	177	LEU	2.0
1	A	1	MET	2.0
1	D	140	CYS	2.0
1	C	33	VAL	2.0
1	B	312	ILE	2.0
1	C	176	ASP	2.0

CONFIDENTIAL VALIDATION REPORT

6.2 Non-standard residues in protein, DNA, RNA chains (i)

There are no non-standard protein/DNA/RNA residues in this entry.

6.3 Carbohydrates (i)

There are no carbohydrates in this entry.

6.4 Ligands (i)

In the following table, the Atoms column lists the number of modelled atoms in the group and the number defined in the chemical component dictionary. The B-factors column lists the minimum, median, 95th percentile and maximum values of B factors of atoms in the group. The column labelled 'Q< 0.9' lists the number of atoms with occupancy less than 0.9.

Mol	Type	Chain	Res	Atoms	RSCC	RSR	B-factors(Å^2)	Q<0.9
2	NAD	D	401	44/44	0.85	0.28	36,52,61,65	0
2	NAD	C	401	44/44	0.90	0.18	15,33,43,49	0
2	NAD	B	401	44/44	0.92	0.18	22,43,54,57	0
2	NAD	A	401	44/44	0.92	0.20	20,45,56,58	0

The following is a graphical depiction of the model fit to experimental electron density of all instances of the Ligand of Interest. In addition, ligands with molecular weight > 250 and outliers as shown on the geometry validation Tables will also be included. Each fit is shown from different orientation to approximate a three-dimensional view.

CONFIDENTIAL VALIDATION REPORT

Electron density around NAD D 401:

$2mF_o$-DF_c (at 0.7 rmsd) in gray
mF_o-DF_c (at 3 rmsd) in purple (negative)
and green (positive)

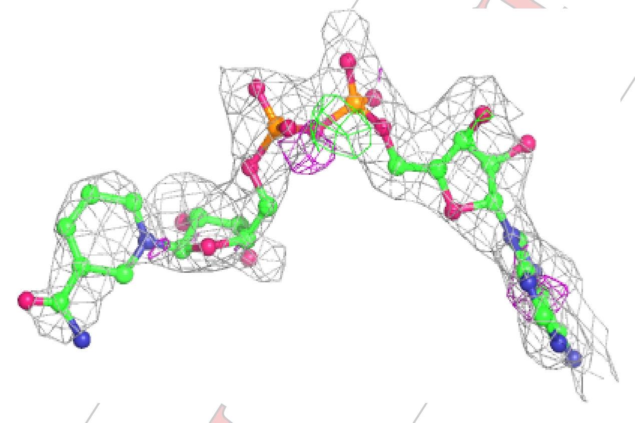

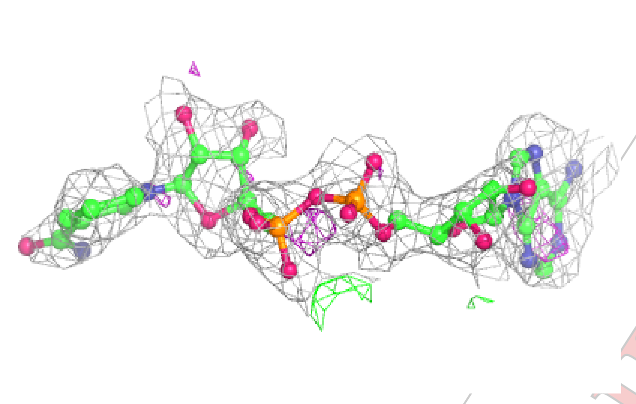

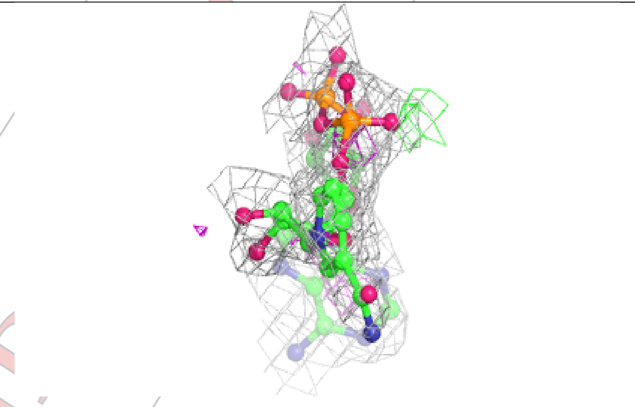

Electron density around NAD C 401:

$2mF_o$-DF_c (at 0.7 rmsd) in gray
mF_o-DF_c (at 3 rmsd) in purple (negative)
and green (positive)

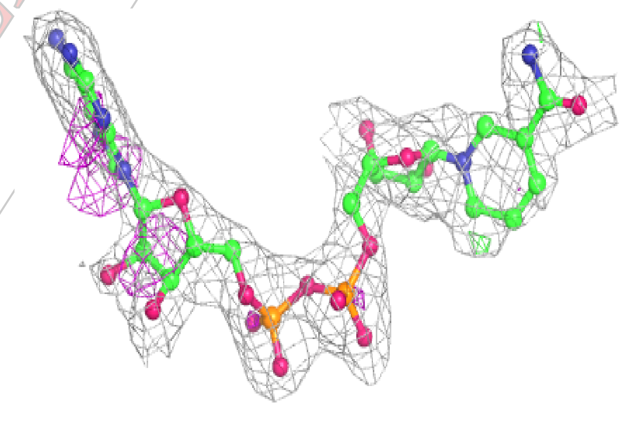

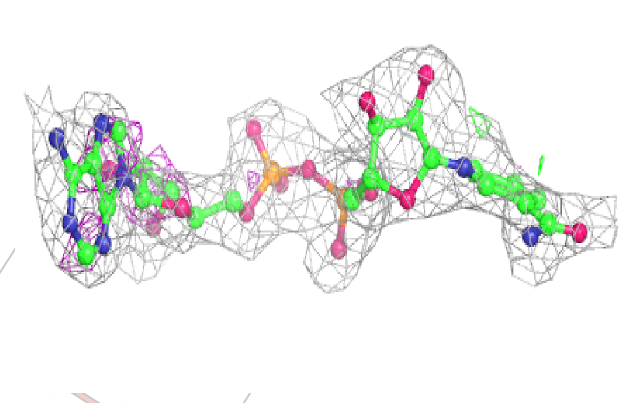

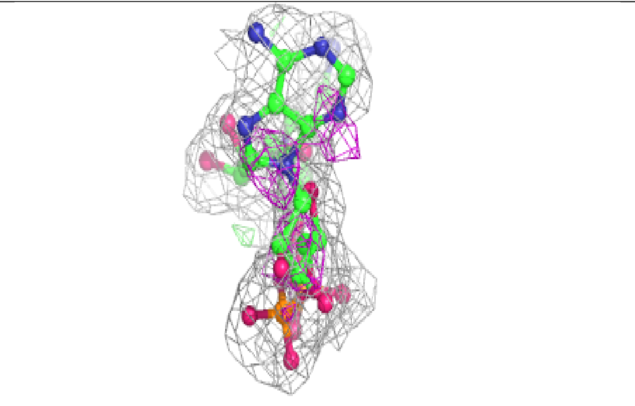

Electron density around NAD B 401:

$2mF_o$-DF_c (at 0.7 rmsd) in gray
mF_o-DF_c (at 3 rmsd) in purple (negative) and green (positive)

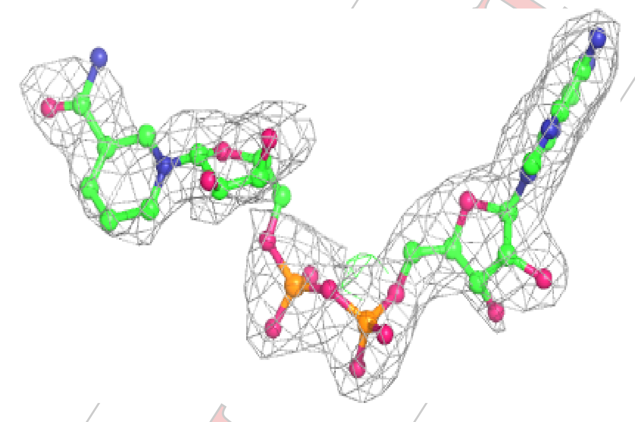

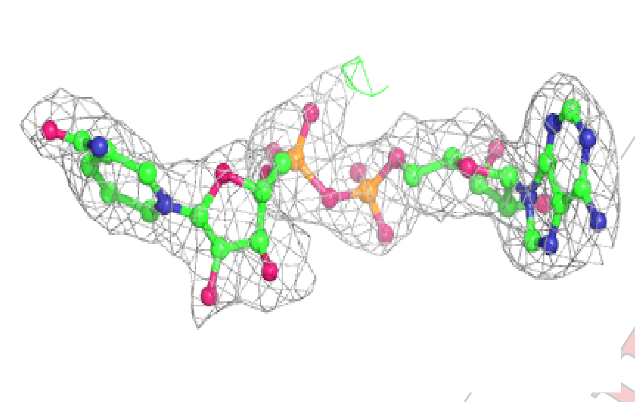

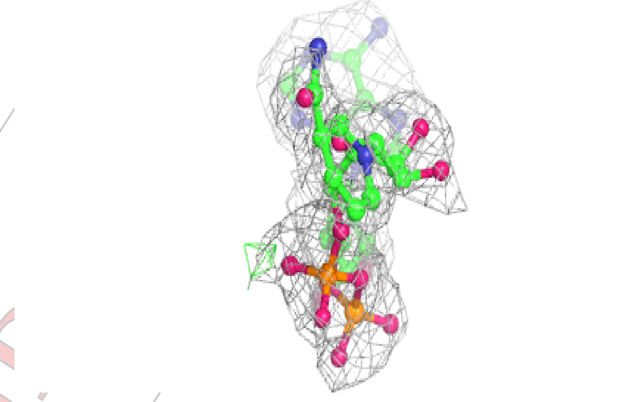

Electron density around NAD A 401:

$2mF_o$-DF_c (at 0.7 rmsd) in gray
mF_o-DF_c (at 3 rmsd) in purple (negative) and green (positive)

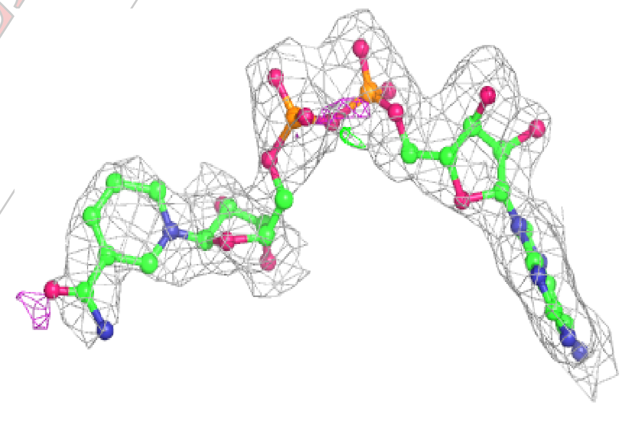

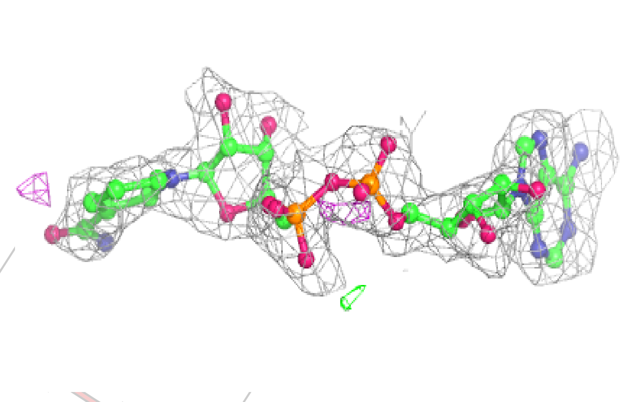

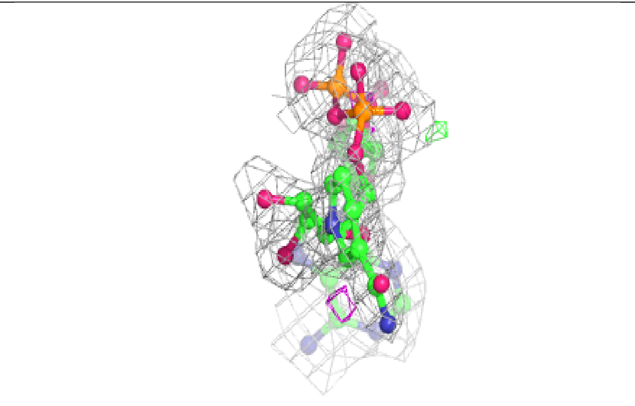

6.5 Other polymers (i)

There are no such residues in this entry.

CONFIDENTIAL VALIDATION REPORT
