# Supplementary material for: A fragment-based approach identifies an allosteric pocket that impacts malate dehydrogenase activity
Source: Commun Biol. 2021 Aug 10;4:949. doi: 10.1038/s42003-021-02442-1 (PMC8355244; doi:10.1038/s42003-021-02442-1)
Supplement: Supplementary file 13 — Supplementary Data 10 [file 42003_2021_2442_MOESM13_ESM.doc]

**MDH WT sequence**

>35250534.seq - ID: 13MW1-T7 on 2017/10/18-8:26:12 automatically edited with PhredPhrap, start with base no.: 35 Internal Params: Windowsize: 20, Goodqual: 19, Badqual: 10, Minseqlength: 50, nbadelimit: 1

TTTtGTTTAACTTtAAGAAGGAGATATACCATGACTAAAATTGCCTTAATAGGTAGTGGTCAAATCGGAGCAATTGTTGGAGAATTGTGTTTGCTGGAAAATCTTGGAGACCTTATTTTATATGATGTAGTCCCAGGTATACCACAAGGAAAGGCTTTAGATTTAAAACATTTTAGTACCATATTAGGAGTAAATAGAAACATTCTTGGTACTAATCAGATCGAAGATATTAAGGATGCAGATATAATAGTTATTACAGCAGGTGTACAAAGAAAAGAAGGAATGACTAGAGAAGATTTGATAGGGGTCAATGGGAAAATAATGAAAAGTGTAGCTGAATCAGTTAAATTACATTGTTCCAAAGCTTTTGTTATTTGTGTTAGTAACCCACTTGATATTATGGTAAATGTTTTTCATAAGTTTAGTAATTTACCTCATGAAAAAATTTGTGGTATGGCAGGTATATTAGATACTTCTAGATATTGTTCATTAATTGCTGATAAATTAAAAGTATCAGCTGAAGATGTTAATGCTGTTATTTTAGGAGGACATGGGGACTTAATGGTACCCTTACAAAGATATACATCGGTAAATGGTGTTCCTTTATCTGAATTTGTCAAGAAAAATATGATTAGCCAAAATGAAATACAAGAAATAATTCAAAAAaCTAGAAATATGGGTGCTGAAATTATTAAACTAGCTAAAGCATCTGCAGCATTTGCCCCAGCTGCTGCTATTACAAAAATGATTAAATCATATTTGTATAATGAAAnTAATTTATTTACATGTGCTgTTtATTTAAATGGACATTATAACTGTTCTAATttATTTGTTGGATCTACTGCTAAAATTAATAATAAGGGGgCACATCCAGTTGAATTCCCTTTAACAAAGGAGGAACAAGATCTTTACACggAaTCGATAGCTAGCGTTCAAAGCAACACAcAAAAAaGCTTtCGaCTTAa

>35250537.seq - ID: 13MW1-pET-RP on 2017/10/18-8:26:12 automatically edited with PhredPhrap, start with base no.: 32 Internal Params: Windowsize: 20, Goodqual: 19, Badqual: 10, Minseqlength: 50, nbadelimit: 1

gttAncaGCCGGATCTCAGTGGTGGTGGTGGTGGTGCTCGAGTGCGGccGCTTTAATTAAGTCGAAAGCTTTTTGTGTGTTGCTTTGAACGCTAGCTATCGATTCCGTGTAAAGATCTTGTTCCTCCTTTGTTAAAGGGAATTCAACTGGATGTGCCCCCTTATTATTAATTTTAGCAGTAGATCCAACAAATAAATTAGAACAGTTATAATGTCCATTTAAATAAACAGCACATGTAAATAAATTATTTTCATTATACAAATATGATTTAATCATTTTTGTAATAGCAGCAGCTGGGGCAAATGCTGCAGATGCTTTAGCTAGTTTAATAATTTCAGCACCCATATTTCTAGTTTTTTGAATTATTTCTTGTATTTCATTTTGGCTAATCATATTTTTCTTGACAAATTCAGATAAAGGAACACCATTTACCGATGTATATCTTTGTAAGGGTACCATTAAGTCCCCATGTCCTCCTAAAATAACAGCATTAACATCTTCAGCTGATACTTTTAATTTATCAGCAATTAATGAACAATATCTAGAAGTATCTAATATACCTGCCATACCACAAATTTTTTCATGAGGTAAATTACTAAACTTATGAAAAACATTTACCATAATATCAAGTGGGTTACTAACACAAATAACAAAAGCTTTGGAACAATGTAATTTAACTGATTCAGCTACACTTTTCATTATTTTCCCATTGACCCcTATCAAATCTTCTCTAGTCATTCCTTCTTTTCTTTGTACACCTGCTGTAATAACTATTATATCTGCATCCTTAATATCTTCGATCTGATTAGTACCAAGAATGTTTCTATTTACTCCTAATATGGTACTAAAATGTtttAanTCTAAAGCCTTTCCTTGTGGTATACCTGGGgACTACATCATAtaAAanTAAGGTCTccAaGaTTTTCCAGCAAnCACAaTTCtCcAACAATTgctcccAaTTTGACCACTACcTATTAaaGgCAATtttaaGtcanggtaannTCnccnTtcttaa

ttaagaAnggnGAnnttaccntgaCttaaaATTGcCttTAATAgGTAGTGGTCAAAtTgggagcAATTGTTgGaGAAtTGTGnTTGCTGGAAAAtCtTggAGACCTTAntTTtaTATGATGTAGTcCCCAGGTATACCACAAGGAAAGGCTTTAGAntTaaaACATTTTAGTACCATATTAGGAGTAAATAGAAACATTCTTGGTACTAATCAGATCGAAGATATTAAGGATGCAGATATAATAGTTATTACAGCAGGTGTACAAAGAAAAGAAGGAATGACTAGAGAAGATTTGATAgGGGTCAATGGGAAAATAATGAAAAGTGTAGCTGAATCAGTTAAATTACATTGTTCCAAAGCTTTTGTTATTTGTGTTAGTAACCCACTTGATATTATGGTAAATGTTTTTCATAAGTTTAGTAATTTACCTCATGAAAAAATTTGTGGTATGGCAGGTATATTAGATACTTCTAGATATTGTTCATTAATTGCTGATAAATTAAAAGTATCAGCTGAAGATGTTAATGCTGTTATTTTAGGAGGACATGGGGACTTAATGGTACCCTTACAAAGATATACATCGGTAAATGGTGTTCCTTTATCTGAATTTGTCAAGAAAAATATGATTAGCCAAAATGAAATACAAGAAATAATTCAAAAAACTAGAAATATGGGTGCTGAAATTATTAAACTAGCTAAAGCATCTGCAGCATTTGCCCCAGCTGCTGCTATTACAAAAATGATTAAATCATATTTGTATAATGAAAATAATTTATTTACATGTGCTGTTTATTTAAATGGACATTATAACTGTTCTAATTTATTTGTTGGATCTACTGCTAAAATTAATAATAAGGGGGCACATCCAGTTGAATTCCCTTTAACAAAGGAGGAACAAGATCTTTACACGGAATCGATAGCTAGCGTTCAAAGCAACACACAAAAAGCTTTCGACTTAATTAAAGCggCCGCACTCGAGCACCACCACCACCACCACTGAGATCCGGCtgnTaac

C-terminal His-tagged pfMDH WT

ATGACTAAAATTGCCTTAATAGGTAGTGGTCAAATCGGAGCAATTGTTGGAGAATTGTGTTTGCTGGAAAATCTTGGAGACCTTATTTTATATGATGTAGTCCCAGGTATACCACAAGGAAAGGCTTTAGATTTAAAACATTTTAGTACCATATTAGGAGTAAATAGAAACATTCTTGGTACTAATCAGATCGAAGATATTAAGGATGCAGATATAATAGTTATTACAGCAGGTGTACAAAGAAAAGAAGGAATGACTAGAGAAGATTTGATAGGGGTCAATGGGAAAATAATGAAAAGTGTAGCTGAATCAGTTAAATTACATTGTTCCAAAGCTTTTGTTATTTGTGTTAGTAACCCACTTGATATTATGGTAAATGTTTTTCATAAGTTTAGTAATTTACCTCATGAAAAAATTTGTGGTATGGCAGGTATATTAGATACTTCTAGATATTGTTCATTAATTGCTGATAAATTAAAAGTATCAGCTGAAGATGTTAATGCTGTTATTTTAGGAGGACATGGGGACTTAATGGTACCCTTACAAAGATATACATCGGTAAATGGTGTTCCTTTATCTGAATTTGTCAAGAAAAATATGATTAGCCAAAATGAAATACAAGAAATAATTCAAAAAACTAGAAATATGGGTGCTGAAATTATTAAACTAGCTAAAGCATCTGCAGCATTTGCCCCAGCTGCTGCTATTACAAAAATGATTAAATCATATTTGTATAATGAAAATAATTTATTTACATGTGCTGTTTATTTAAATGGACATTATAACTGTTCTAATTTATTTGTTGGATCTACTGCTAAAATTAATAATAAGGGGGCACATCCAGTTGAATTCCCTTTAACAAAGGAGGAACAAGATCTTTACACGGAATCGATAGCTAGCGTTCAAAGCAACACACAAAAAGCTTTCGACTTAATTAAAGCggCCGCACTCGAGCACCACCACCACCACCACTGA

**M**TKIALIGSGQIGAIVGELCLLENLGDLILYDVVPGIPQGKALDLKHFSTILGVNRNILGTNQIEDIKDADIIVITAGVQRKEGMTREDLIGVNGKIMKSVAESVKLHCSKAFVICVSNPLDIMVNVFHKFSNLPHEKICGMAGILDTSRYCSLIADKLKVSAEDVNAVILGGHGDLMVPLQRYTSVNGVPLSEFVKKNMISQNEIQEIIQKTRNMGAEIIKLAKASAAFAPAAAITKMIKSYLYNENNLFTCAVYLNGHYNCSNLFVGSTAKINNKGAHPVEFPLTKEEQDLYTESIASVQSNTQKAFDLIKAAALEHHHHHH
