# Supplementary material for: A fragment-based approach identifies an allosteric pocket that impacts malate dehydrogenase activity
Source: Commun Biol. 2021 Aug 10;4:949. doi: 10.1038/s42003-021-02442-1 (PMC8355244; doi:10.1038/s42003-021-02442-1)
Supplement: Supplementary file 14 — Reporting Summary [file 42003_2021_2442_MOESM14_ESM.pdf]

nature research

Corresponding author(s): Prof.Matthew Groves

Last updated by author(s): 2021-5-29

Reporting Summary

Nature Research wishes to improve the reproducibility of the work that we publish. This form provides structure for consistency and transparency in reporting. For further information on Nature Research policies, see our Editorial Policies and the Editorial Policy Checklist.

Statistics

For all statistical analyses, confirm that the following items are present in the figure legend, table legend, main text, or Methods section.

n/a	Confirmed	
☐	☒	The exact sample size (*n*) for each experimental group/condition, given as a discrete number and unit of measurement
☐	☒	A statement on whether measurements were taken from distinct samples or whether the same sample was measured repeatedly
☐	☒	The statistical test(s) used AND whether they are one- or two-sided<br>*Only common tests should be described solely by name; describe more complex techniques in the Methods section.*
☒	☐	A description of all covariates tested
☐	☒	A description of any assumptions or corrections, such as tests of normality and adjustment for multiple comparisons
☐	☒	A full description of the statistical parameters including central tendency (e.g. means) or other basic estimates (e.g. regression coefficient) AND variation (e.g. standard deviation) or associated estimates of uncertainty (e.g. confidence intervals)
☐	☒	For null hypothesis testing, the test statistic (e.g. *F*, *t*, *r*) with confidence intervals, effect sizes, degrees of freedom and *P* value noted<br>*Give P values as exact values whenever suitable.*
☒	☐	For Bayesian analysis, information on the choice of priors and Markov chain Monte Carlo settings
☒	☐	For hierarchical and complex designs, identification of the appropriate level for tests and full reporting of outcomes
☒	☐	Estimates of effect sizes (e.g. Cohen's *d*, Pearson's *r*), indicating how they were calculated

Our web collection on statistics for biologists contains articles on many of the points above.

Software and code

Policy information about availability of computer code

Data collection	STD-NMR: TopSpin® software, 4.0. MST: MO.Control 2 (Nano-temper Technologies, GmbH); TSA: CFX96™ real-time PCR collection machine; Crystallization: XDS (data processing), XSCALE (data reduction and scaling), CTRUNCATE (amplitude calculation); REFMAC5 (data refinment); Activity assay: Microsoft Excel 2019 and Tecan Spark control software; Small-angle X-ray scattering experiments: Foxtrot package (data reduction);
Data analysis	STD-NMR: TopSpin® software, 4.0. MST: MO.Affinity Analysis 3 (Nano-temper Technologies, GmbH); TSA: CFX96™ control software; Crystallization: MOLPROBITY (model quality analysis); Activity assay: Prism 8.0 (Graphpad); Computational analysis: Scorpion™ (Desertsci) and Pymol (The PyMOL Molecular Graphics System, Version 2.0 Schrödinger, LLC), AutoDock Vina (docking); SWISS-MODEL (homology modeling); Small-angle X-ray scattering experiments: Primus (ATSAS package, structural parameters calculation)

For manuscripts utilizing custom algorithms or software that are central to the research but not yet described in published literature, software must be made available to editors and reviewers. We strongly encourage code deposition in a community repository (e.g. GitHub). See the Nature Research guidelines for submitting code & software for further information.

Data

Policy information about availability of data

All manuscripts must include a data availability statement. This statement should provide the following information, where applicable:

- Accession codes, unique identifiers, or web links for publicly available datasets
- A list of figures that have associated raw data
- A description of any restrictions on data availability

Crystal structures in the presence of the ligand or cofactor were deposited in the Protein Data Bank (https://www.rcsb.org/) under accession codes 6R8G and 6Y91. The SAXS data have been deposited in the SASBDB59 under the access codes SASDLQ2 (PfMDH L-lactate dehydrogenase, apo), SASDLR2 (PfMDH L-lactate

dehydrogenase bound to inhibitor 2a) and SASDLS2 (PfMDH L-lactate dehydrogenase bound to inhibitor 6a). The data that support the findings of this study are available from the authors on reasonable request, see author contributions for specific data sets.

Field-specific reporting

Please select the one below that is the best fit for your research. If you are not sure, read the appropriate sections before making your selection.

[x] Life sciences [ ] Behavioural & social sciences [ ] Ecological, evolutionary & environmental sciences

For a reference copy of the document with all sections, see nature.com/documents/nr-reporting-summary-flat.pdf

Life sciences study design

All studies must disclose on these points even when the disclosure is negative.

Sample size	Thermal shift assay: triplicate.<br>Microscale thermophoresis: triplicate.<br>Kinetics experiments: triplicate.<br>Chemical compounds screening: duplicate. To maximize signal intensity, we chose 250 µl reaction volume.
Data exclusions	The 8mM dose of 4DT in MST experiments was excluded because of the photobleaching effect at high ligand concentrations.<br>A precipitation of compound was observed for the para-isopropyl (compound 10a), para-nitro (compound 2a), meta-trifluoro (compound 8a) and para-bromo (compound 6a) derivatives, due to their solubility in the assay buffer. Thereby, the effect of these compound on protein activity has been excluded. Dose response of 4DT is not presented as well due to its low solubility at 4 mM concentration in water.
Replication	To verify the reproducibility of the results, we repeated the experiments at least in duplicate (4DT derivatives only). The remainder was done in triplicate.
Randomization	Randomization was not used in this paper as this is an early-stage drug discovery study.
Blinding	We did not opt for the blinding process because this study did not involve clinical trials (i.e. control vs intervention)

Reporting for specific materials, systems and methods

We require information from authors about some types of materials, experimental systems and methods used in many studies. Here, indicate whether each material, system or method listed is relevant to your study. If you are not sure if a list item applies to your research, read the appropriate section before selecting a response.

Materials & experimental systems

n/a	Involved in the study
[x]	[ ] Antibodies
[x]	[ ] Eukaryotic cell lines
[x]	[ ] Palaeontology and archaeology
[x]	[ ] Animals and other organisms
[x]	[ ] Human research participants
[x]	[ ] Clinical data
[x]	[ ] Dual use research of concern

Methods

n/a	Involved in the study
[x]	[ ] ChIP-seq
[x]	[ ] Flow cytometry
[x]	[ ] MRI-based neuroimaging
